# A Rahman Syndrome mutation in histone H1.4 disrupts chromatin compaction and phase separation

**DOI:** 10.1038/s41467-026-73046-8

**Published:** 2026-05-22

**Authors:** Ramachandran Boopathi, Isabel Garcia-Saez, Serhan Turunç, Imtiaz Nisar Lone, Ashok Kumar, Abed Alkarem Abu Alhaija, Jeffrey J. Hayes, Jan Bednar, Muhammed Kasim Diril, Dimitar Iliev, Anastas Gospodinov, Aline Le Roy, Dimitrios Skoufias, Dimitar Angelov, Ali Hamiche, Seyit Kale, Stefan Dimitrov, Carlo Petosa

**Affiliations:** 1 Univ. Grenoble Alpes, CNRS, CEA, Institut de Biologie Structurale (IBS), Grenoble, France; 2 Izmir Biomedicine and Genome Center, Dokuz Eylül University Health Campus, Izmir, Turkey; 3 Izmir International Biomedicine and Genome Institute, Dokuz Eylül University, Izmir, Turkey; 4 University of Rochester Medical Center, Rochester, NY USA; 5 Univ. Grenoble Alpes, Inserm, CNRS, Institute for Advanced Biosciences (IAB), Grenoble, France; 6 Roumen Tsanev Institute of Molecular Biology, Bulgarian Academy of Sciences, Sofia, Bulgaria; 7 Institut de Génétique et Biologie Moléculaire et Cellulaire (IGBMC), Université de Strasbourg, CNRS, INSERM, Illkirch, France; 8 Faculty of Medicine, Izmir Katip Çelebi University, Izmir, Turkey

**Keywords:** Molecular modelling, SAXS, Biochemical assays, Fluorescence imaging, Nucleosomes

## Abstract

Rahman syndrome is a rare developmental disorder caused by frameshift mutations in linker histone H1.4 that produce a truncated carboxy-terminal domain with reduced positive charge. We investigated the effects of a disease-associated mutation on chromatin structure and dynamics, focusing on H1.4-bound nucleosomes and hexanucleosomal arrays. We report that this mutation induces a more extended and flexible array conformation, characterized by enhanced linker DNA accessibility and an inability to form compact, regularly stacked nucleosome structures. Notably, mutant H1.4-bound arrays show a reduced capacity to undergo liquid-liquid and liquid-solid phase separation, closely resembling linker histone-free arrays. Molecular dynamics simulations corroborated by fluorescence resonance energy transfer measurements indicate that the mutated carboxy-terminal domain interacts with a shorter linker DNA segment, resulting in a more open nucleosome conformation. Consistent with these structural changes, the mutation significantly enhances H1.4 mobility within cell nuclei, reflecting a weaker chromatin association. The combined data suggest that Rahman syndrome-associated mutations promote an aberrantly relaxed chromatin state, potentially leading to the dysregulation of gene expression that may drive disease pathology. These findings underscore the essential role of the carboxy-terminal domain in chromatin compaction and provide mechanistic insights into the molecular etiology of Rahman syndrome.

## Abstract (web summary)

A mutation linked to Rahman syndrome disrupts the ability of linker histone H1.4 to compact nucleosome arrays, producing a more open and dynamic chromatin state with impaired phase separation and providing insight into disease mechanisms.

## Introduction

Rahman syndrome (RS), also known as HISTH1E syndrome, is a rare, recently identified autosomal-dominant disorder characterized by intellectual disability and distinctive facial features^1–3^. Typical symptoms include neonatal hypotonia, premature aging appearance, cryptorchidism, skeletal and cardiac anomalies, abnormal dentition, visual disturbances, behavioral issues and brain magnetic resonance imaging (MRI) anomalies, particularly in the corpus callosum. RS is attributed to germline monoallelic frameshift mutations in the *H1-4* (formerly *HIST1H1E*) gene, which encodes histone H1.4, a member of the H1 linker histone family. Histones are structural components of the nucleosome, the basic organizing unit of chromatin, comprising a nucleosome core particle (NCP), linker DNA, and a linker histone. The NCP contains ~147 DNA base pairs (bp) wrapped around an octamer of core (H2A, H2B, H3 and H4) histones^4^ and connects to adjacent NCPs by a variable length of linker DNA. Linker histone H1 binds to the NCP between the entry and exit DNA linker arms, rigidifying and drawing together the two DNA linkers into an apposed linker stem motif within a structure dubbed the chromatosome, comprising 160 bp of DNA, the core histone octamer and H1^5–10^.

Mammals possess 11 different H1 isoforms believed to have distinct functions, with 4 expressed in the germline and 7 expressed in somatic cells, the latter including 5 DNA replication-dependent (H1.1 through H1.5) and 2 replication-independent (H1.0, H1x) subtypes^11–13^. While early studies identified H1 as a general transcriptional repressor^14,15^, certain H1 variants were later also linked to active gene expression^16–18^. Linker histones share a tripartite structure composed of an N-terminal tail (~20 residues), a conserved central globular domain (GH1 domain, ~80 residues) and an unstructured, lysine-rich carboxy-terminal domain (CTD, ~100 residues)^19–22^. The globular domain adopts a winged-helix fold and is sufficient for nucleosome binding^5,19^. The CTD is required for linker stem formation^7^ and for stabilizing secondary chromatin structures^19,20^.

**Fig. 1:**
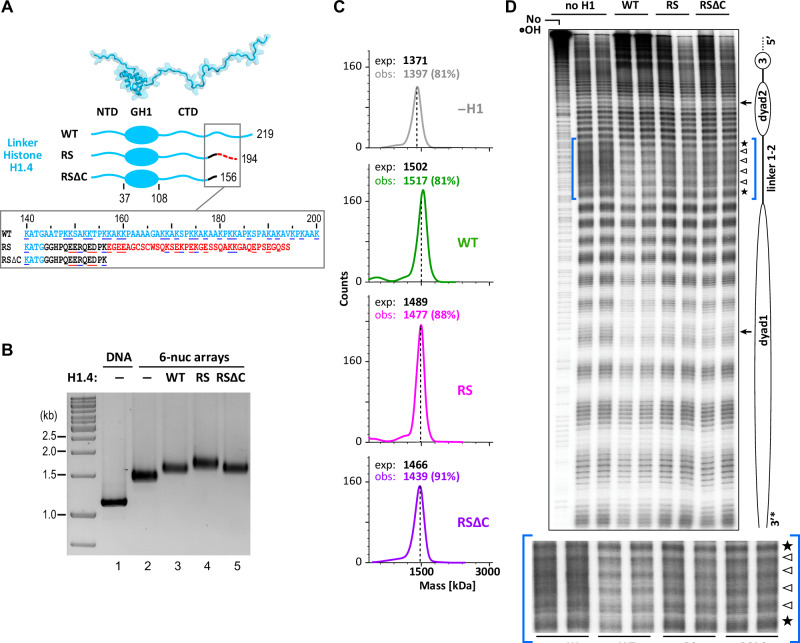
The RS mutation enhances linker DNA accessibility. **A** H1.4 domain organization and mutants. Basic and acidic residues are underlined in blue and red, respectively. The AlphaFold2 model of WT H1.4 is shown. **B** Native agarose gel of 6 × 187 bp hexanucleosome arrays reconstituted in the absence (lane 2) or presence (lanes 3–5) of the WT, RS mutant or RSΔC mutant forms of H1.4. Naked DNA was included as a control (lane 1). The single band in lane 2 and its shift from lane 1 confirm hexanucleosome reconstitution. The single shifted bands in lanes 3–5 indicate efficient H1.4 incorporation and high sample homogeneity. Despite its reduced net positive charge, the RS variant results in arrays that migrate more slowly than WT, consistent with a more extended array conformation and possibly increased hydrodynamic drag from its disordered, acidic C-terminal tail. RSΔC-bound arrays migrate faster than those with RS, consistent with loss of the 38-residue (~4 kDa) acidic tail, despite the higher net positive charge of the RSΔC variant (+9 vs. +6). Experiments shown in (**B**) and (**D**) were each repeated independently three times with similar results. **C** Mass photometry analysis of 6 × 187 bp hexanucleosome arrays reconstituted in the absence (gray) or presence of WT (green), RS mutant (magenta) or RSΔC mutant (purple) H1.4. The expected (exp) mass of the array and the observed (obs) mass determined from the main peak are indicated. The percentage of particles within the main peak is in parentheses. Experiments were performed in a buffer containing 10 mM NaCl. **D** Hydroxyl radical footprinting analysis of 6 × 197 bp hexanucleosome arrays reconstituted in the absence or presence of the indicated H1.4. Arrows indicate the dyad in nucleosomes N1 and N2. Open arrowheads indicate cleavage products within the central part of the linker DNA. Asterisks indicate the 10 bp at either end of the linker DNA adjacent to the core region. No •OH, free DNA. *Lower panel*. Magnified view of the N1-N2 linker region. Note the more smeared appearance of the RS and RSΔC lanes. Source data are available in the source data file.

Histone H1.4 is a ubiquitously expressed 219-residue protein that shares >70% identity with the other replication-dependent H1 isoforms. H1.4 regulates chromatin compaction during mitosis by modulating the binding of HP1 to chromatin^23,24^, and may function as either a transcriptional repressor or activator depending on its post-translational modifications (reviewed in ref. ^25^). The mutations identified in RS patients all cluster within a 145-bp stretch in the CTD-encoding region of *H1-4* (Supplementary Fig. 1). These involve small insertions or deletions that yield the same frameshift, generating truncated proteins with a shared 38-residue divergent C-terminal motif that is enriched in acidic amino acids (Fig. 1A)^2,3^. Accordingly, these H1.4 mutants contain a CTD with a dramatically reduced positive, and in some cases even slightly negative, overall charge (Supplementary Fig. 1). Since the positively charged nature of the H1 CTD is critical for chromatin condensation^22^, nuclear domains associated with RS-associated H1.4 variants are expected to exhibit less compact structures, potentially resembling those associated with embryonic H1 subtypes^26–29^. Functional studies have shown that RS-associated mutations result in stable proteins that localize to the nucleus, bind to chromatin, disrupt DNA compaction and induce altered histone H3 and DNA methylation patterns, leading to dramatically reduced cell proliferation rates, limited S phase entry, and accelerated cellular senescence^2,30^. These findings suggest that the cellular pathophysiology in RS arises from gain-of-function or dominant negative effects of the mutant protein^2^. Moreover, exogenous expression of an RS-associated H1.4 variant in primary rat neurons revealed disruptions in synaptic gene expression and in action potential frequency and synchrony, which may underlie the neurological deficits seen in RS patients^31^. Despite these advances, however, the detailed molecular basis of RS remains poorly understood.

To shed light on this question, we investigated the impact of an RS-associated mutation on the conformational and dynamic properties of chromatin. We focused our efforts on H1.4-bound nucleosomes and hexanucleosomes, which model key properties of extended nucleosome arrays^32–34^. Our findings reveal that an RS-associated H1.4 variant induces a more extended and flexible hexanucleosome conformation, increasing linker DNA accessibility and impairing chromatin condensation and phase separation. The mutation also enhances the mobility of H1.4 within cell nuclei, reflecting a weaker chromatin association. Comparison with a C-terminal truncation mutant highlights the functional significance of the C-terminal acidic motif. Our findings suggest that RS-associated variants promote a relaxed chromatin conformation by weakening CTD-linker interactions and increasing the angular divergence between nucleosomal DNA linkers. We speculate that such alterations likely contribute to RS pathogenesis in vivo.

## Results

### The RS mutation enhances linker DNA accessibility

We began by examining how an RS-associated mutation affects the ability of histone H1.4 to interact with a hexanucleosome array. As a representative mutation for use throughout this study, we focused on the c.430dupG variant, the most frequently identified variant among all RS patients^3^. This variant arises from a frameshift mutation located midway within the spectrum of known RS-associated mutations, which shortens the CTD by 25 residues and reduces its net charge from +43 to +6 (Supplementary Fig. 1). We reconstituted arrays using DNA that consisted of six tandem repeats of the Widom 601 positioning element, in combination with recombinant human core histones, either with or without recombinant H1.4 (Supplementary Fig. 2A). We used 6 × 187 base-pair (bp) arrays for all array-based experiments except hydroxyl radical footprinting, which used 6 × 197 bp arrays, as the longer linker DNA improves resolution of the 10-bp periodicity in the linker stem region. Except for the linker histone, these arrays are identical to those we previously characterized in complex with *Xenopus laevis* histone H1.0b (H1.0b^*X.lae*^)^34^.

We reconstituted arrays with one of three forms of H1.4: the wild type, the RS-associated c.430dupG variant, or a truncated version of the latter lacking the negatively charged 38-residue C-terminal motif (hereafter WT, RS and RSΔC, respectively**;** Fig. 1A). We included the RSΔC mutant—an artificial variant not observed in patients—as an investigative tool to assess whether the behavior of the RS variant arises from the loss of C-terminal WT residues or from the acquisition of the acidic C-terminal motif. Electrophoretic mobility shift assays (EMSAs) of mono- and hexanucleosomes reconstituted with increasing amounts of each H1.4 variant confirmed comparable incorporation efficiencies of the WT, RS, and RSΔC forms (Supplementary Fig. 3). All hexanucleosome preparations used in subsequent experiments displayed a single band on a native agarose gel, indicating high sample homogeneity (Fig. 1B and Supplementary Fig. 2B). Consistent with these results, mass photometry analysis, which reports on the molecular mass of particles in solution^35,36^, revealed a predominant peak at the expected unbound or H1.4-bound hexanucleosome mass and no detectable aggregates (Fig. 1C). Modeling of the mass distribution data further indicated similar H1 occupancies across the three H1-bound arrays (Supplementary Fig. 4). Together, these results confirm that all three H1.4 variants bind efficiently to nucleosome arrays, yielding homogeneous samples suitable for comparative structural and functional analyses.

To analyze how the above linker histones bind specifically to the nucleosome array, we mapped the H1.4-DNA interactions at single nucleotide resolution by hydroxyl radical footprinting (Fig. 1D). The analysis, performed under ionic conditions that induce chromatin folding, revealed an H1.4-dependent footprinting pattern resembling that previously observed with H1.0b^*X.lae*^ on isolated mono- and hexanucleosomes^7,9,34^. In agreement with the previous studies, all three H1.4 variants displayed pronounced protection at the nucleosomal dyads, consistent with an on-dyad binding mode of their GH1 domains, as observed in the cryo-EM structure of an H1.4-containing chromatosome^10^. However, compared to WT H1.4, the RS and RSΔC mutants altered the characteristic 10-bp footprinting pattern of the linker DNA, resulting in a more smeared appearance of gel bands, approaching that seen in the absence of H1.4 and indicating a greater degree of hydroxyl radical cleavage of the linker DNA (Fig. 1D). A footprinting experiment performed on dinucleosomes revealed a similar enhanced cleavage of the linker region in the presence of mutant forms of H1.4 (Supplementary Fig. 2C, D). These observations suggest that the DNA linkers within the RS- and RSΔC-bound di- and hexanucleosomes are more solvent accessible than those bound to WT H1.4.

### The RS mutation induces a more extended and flexible nucleosome array

**Fig. 2:**
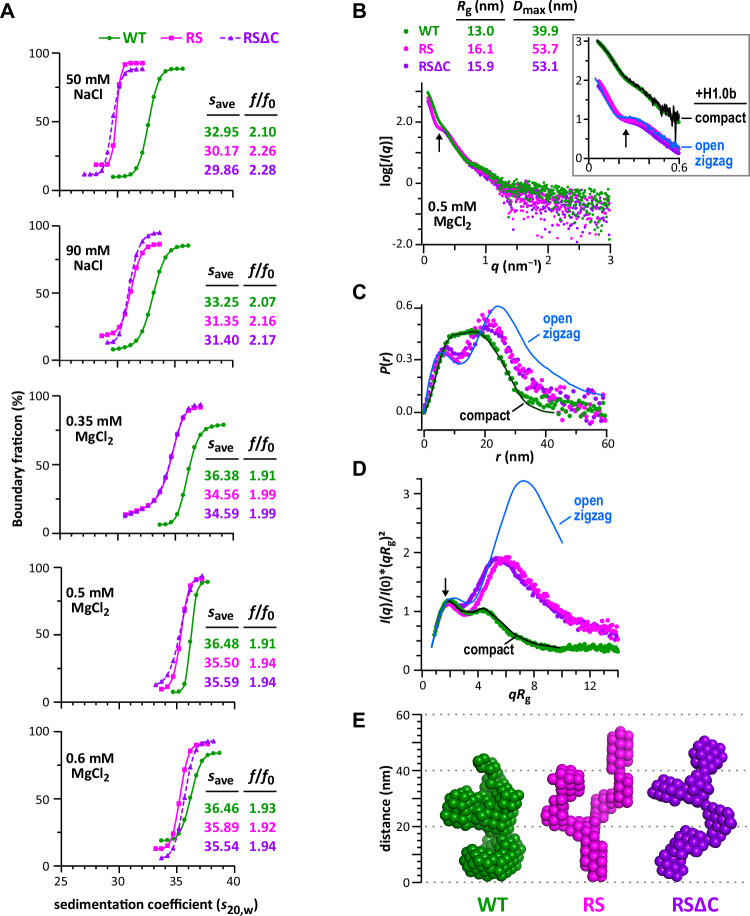
The RS mutation induces a more extended and flexible nucleosome array. Data for 6 × 187 bp hexanucleosome arrays bound to WT H1.4 or to the RS or RSΔC mutants are in green, magenta and purple, respectively. **A** Sedimentation velocity analysis of hexanucleosome arrays prepared in buffers containing NaCl or MgCl_2_, as indicated. The average sedimentation coefficient (*s*_ave_) and frictional ratio (*f*/*f*_0_) are indicated for the principal species sedimenting in the 20- to 40-S range. **B–E** SAXS analysis of H1.4-bound hexanucleosome arrays in a buffer containing 0.5 mM MgCl_2_. Previously reported data^34^ from H1.0b^*Xlae*^-bound arrays prepared in buffers containing 1 mM (blue) or 90 mM (black) NaCl are included for comparison and labeled “open zigzag” and “compact”, respectively. **B** Scattering curves and summary of model-independent parameters. The arrow indicates the shoulder observed in the mutant curves at *q* = 0.25 nm^−1^. *Inset*. Close-up view of low-resolution data. Curves are arbitrarily displaced along the vertical axis for clarity. **C** Distance probability distribution. The vertical axis is in arbitrary units. **D** Normalized Kratky plot. The arrow indicates the peak at *qR*_g_ = 1.7 characteristic of globular structures. **E** Ab initio bead models. Source data are available in the source data file.

Enhanced DNA linker accessibility suggests that the RS- and RSΔC-bound hexanucleosome arrays may adopt a more open structure compared to WT arrays. To test this hypothesis, we used analytical ultracentrifugation (AUC) to measure the sedimentation velocity of H1.4-bound hexanucleosomes under various ionic conditions. In low millimolar (50 and 90 mM) NaCl, hexanucleosomes bound to WT H1.4 displayed an average sedimentation coefficient (*s*_ave_) of ~33 S, while the presence of submillimolar (0.35–0.6 mM) MgCl_2_ greatly increased their sedimentation rate (*s*_ave_ ≈ 36.5 S) (Fig. 2A), consistent with previous AUC analyses of H1.0b^*X.lae*^-bound hexanucleosomes^34^. Across all ionic conditions tested, arrays containing the RS and RSΔC forms of H1.4 behaved very similarly, consistently sedimenting more slowly than WT arrays (Fig. 2A and Supplementary Fig. 5A). This difference in sedimentation behavior was especially pronounced at 50–90 mM NaCl and 0.35 mM MgCl_2_. Since the sedimentation rate reflects the degree of particle compaction, this finding suggests that RS- and RSΔC-bound arrays adopt a much more extended conformation than WT arrays, and that increasing the MgCl_2_ concentration mitigates this effect.

To further study these conformational differences, we analyzed H1.4-bound hexanucleosomes by small-angle X-ray scattering (SAXS), which provides three-dimensional (3D) structural information on macromolecules in solution (Supplementary Table 1). Across the range of ionic conditions tested, the RS- and RSΔC-bound arrays showed similar scattering profiles that differed consistently from those of WT H1.4-bound arrays (Supplementary Fig. 5B). In 0.5 mM MgCl_2_, WT arrays yielded a scattering curve closely resembling that previously observed for H1.0b^*Xlae*^-bound hexanucleosomes under conditions favoring array compaction^34^ (Fig. 2B, green vs. black curves). In contrast, the RS- and RSΔC-bound arrays exhibited a distinct shoulder in the scattering curve (Fig. 2B, arrow), similar to that observed for H1.0b^*Xlae*^-bound arrays under conditions inducing a completely extended (“open zigzag”) conformation^34^ (Fig. 2B, magenta and purple vs. blue curves). Accordingly, the radius of gyration (*R*_g_) and maximal diameter (*D*_max_) were markedly higher for the mutant arrays compared to the WT.

In the distance probability distribution, WT arrays showed a single broad peak centered at ~15 nm, resembling that previously reported for compact H1.0b^*Xlae*^-bound arrays (Fig. 2C and Supplementary Fig. 5B). In contrast, the mutant arrays yielded a bimodal distribution resembling that reported for the open zigzag conformation of H1.0b^*Xlae*^-bound arrays, except that the larger peak was shifted towards shorter distances (with a maximum at 21 instead of 24 nm). This confirms that the mutant arrays are more extended than WT arrays, although less so than a fully open zigzag structure. In the normalized Kratky plot, the WT and mutant arrays share a common peak (at *qR*_g_ = 1.7; Fig. 2D, arrow) characteristic of globular structures^37^, consistent with the presence of well-folded nucleosomal units. The increase at higher *qR*_g_ values indicates that the mutant arrays are more flexible than WT arrays^38^, although less flexible than a fully open zigzag. Ab initio modeling suggests that, compared to WT, mutant arrays adopt a more relaxed conformation characterized by loosely connected nucleosome-sized units, which is maintained even in the most compacting conditions (0.6 mM MgCl_2_ or 90 mM NaCl) (Fig. 2E, Supplementary Fig. 5C and Supplementary Table 2). Overall, these data indicate that the RS and RSΔC mutants are less efficient than WT H1.4 at condensing hexanucleosomes, resulting in more extended and flexible arrays.

### The RS mutation hampers the proper folding of nucleosome arrays

**Fig. 3:**
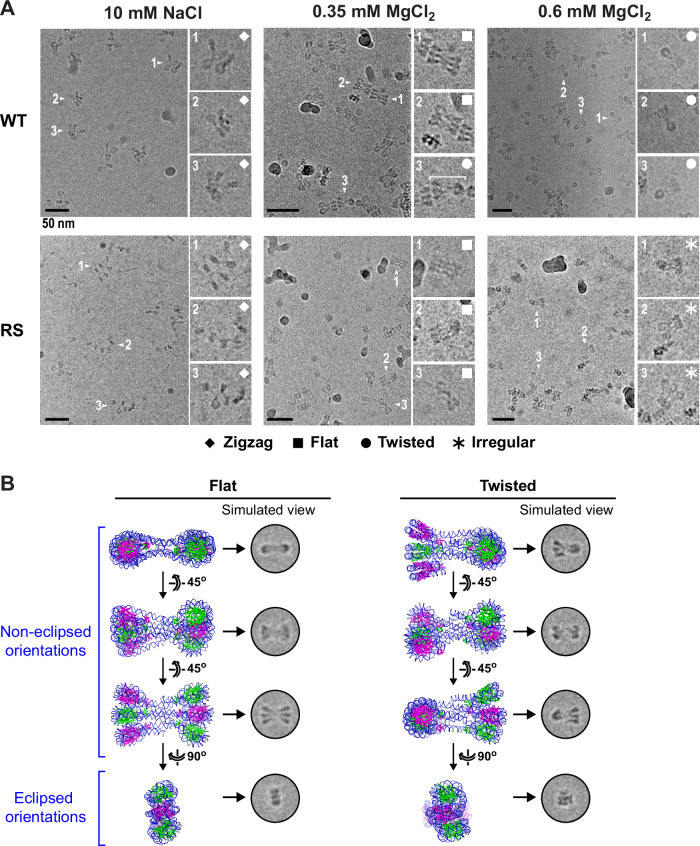
Cryo-EM analysis of H1.4-bound nucleosome arrays in different states of compaction. **A** Field views of 6 × 187 bp hexanucleosomes bound to the WT or RS mutant forms of H1.4 under the indicated ionic conditions. The scale bar is 50 nm. *Insets*. Closeup view of particles indicated by numbered arrowheads. The zigzag, flat, twisted and irregular conformations are indicated by a diamond, square, circle and asterisk, respectively. The experiment was independently repeated twice with similar results. **B** Flat and twisted hexanucleosome structures with their corresponding simulated views, as previously described^34^.

We next investigated how the RS mutation affects the structure of the hexanucleosome using cryo-electron microscopy (cryo-EM). Although strong aggregation of H1.4-bound arrays during grid preparation precluded 3D reconstruction, the few isolated particles that could be imaged nevertheless yielded valuable insights (Fig. 3A). Previous work on H1.0b^*X.lae*^-bound hexanucleosomes showed that arrays imaged at low (5–10 mM) NaCl concentration display a zigzag configuration, whereas those in 0.35 or 0.6 mM MgCl_2_ exhibit a two-start helical arrangement of stacked nucleosomes, adopting either a flat or twisted conformation, with the twisted form predominating at the higher Mg^2+^ concentration^34^ (Fig. 3B). Arrays bound to WT H1.4 recapitulated this conformational behavior (Fig. 3A). Arrays bound to the RS mutant also behaved similarly, but appeared qualitatively more extended at low NaCl and less well folded in Mg^2+^, particularly at the higher concentration, suggesting that the RS mutation hinders arrays from adopting the twisted state.

**Fig. 4:**
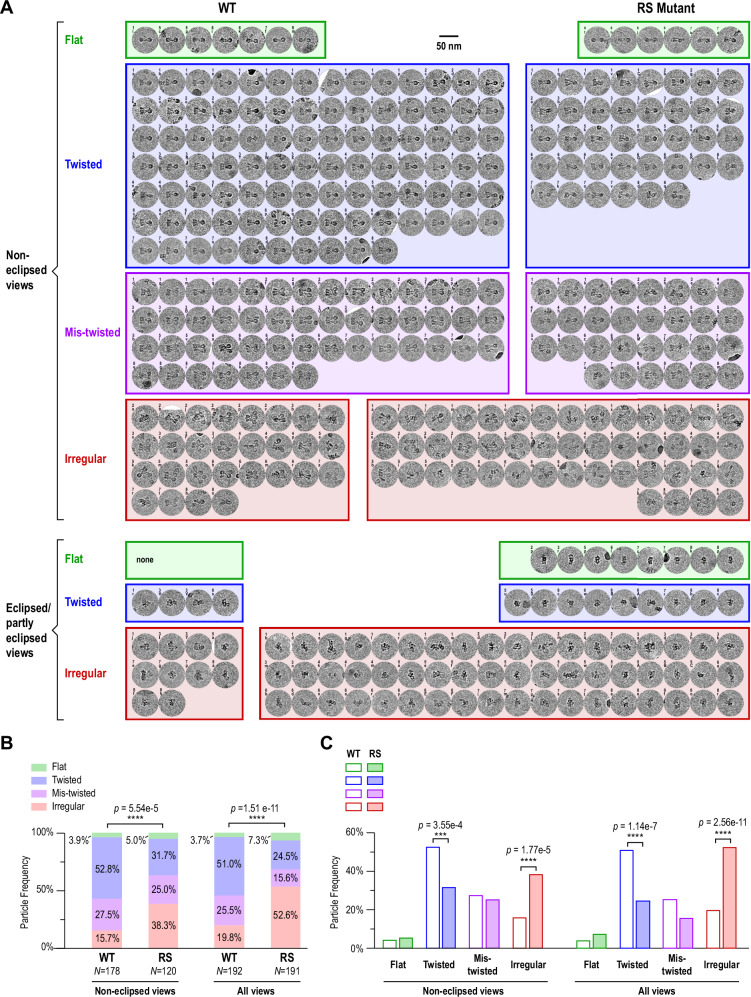
The RS mutation hampers proper folding of nucleosome arrays. **A** Cryo-EM images of hexanucleosomes bound to WT H1.4 or the RS mutant in 0.6 mM MgCl_2_. Images correspond to particles from the micrographs in Supplementary Fig. 6, rotated for approximate alignment. The number and letter in the upper left indicate the micrograph and particle index, respectively, as labeled in Supplementary Fig. 6. **B** Stacked bar plots showing the distribution of particle conformations for non-eclipsed views (left) and all orientations (right). WT and RS mutant distributions differed significantly (chi-square test). **C** Grouped bar plots comparing conformational class frequencies of WT (open) and RS mutant (filled) arrays. WT arrays were enriched in twisted conformations, whereas RS mutant arrays were enriched in irregular forms. Both differences remained significant after Bonferroni correction (adjusted cutoff, *p* < 0.0125; two-sided Fisher’s exact test).

To confirm this hypothesis, we analyzed arrays at 0.6 mM MgCl_2_ in greater detail, considering both aggregated and isolated particles. Under this condition, both WT and RS mutant samples displayed mixtures of well- and poorly folded particles (Supplementary Fig. 6). However, WT aggregates frequently contained several closely packed, properly twisted particles—most evident in clusters of two or three arrays—whereas RS clusters rarely included more than one such particle and were typically composed entirely of irregular or poorly folded arrays (Supplementary Fig. 7). To assess the mutation’s effect on isolated arrays, we extracted all clearly discernible, well separated particles (~190 per sample) and classified them by comparison to simulated views of flat and twisted hexanucleosomes (Fig. 4A). A technical point is that most WT particles (93%) adopted non-eclipsed orientations, where both nucleosomal stacks of the two-start helix were visible, whereas ~37% of mutant particles adopted eclipsed or partly eclipsed orientations, in which one stack occluded the other (as illustrated in Fig. 3B). We classified non-eclipsed particles as well-folded (flat or twisted) or poorly folded, with the latter including irregular arrays and a recurrent “mis-twisted” conformation in which one nucleosome is displaced from its stack (Fig. 4A). We separately scored eclipsed particles as twisted, flat, or irregular, since mis-twisted forms could not be reliably identified in this orientation. Whether considering all particles or only the non-eclipsed subset, the WT and RS samples showed significantly different distributions (Fig. 4B), with WT arrays containing more twisted conformations and RS arrays enriched in irregular forms (Fig. 4C). Together, these findings indicate that the RS mutant hinders arrays from adopting the compact twisted state, in both isolated and clustered particles.

### The RS mutation impairs chromatin phase separation

In vitro studies have established that nucleosome arrays adopt an extended conformation under low-salt conditions and fold into compact fibers with increased cation concentration^34,39,40^. Above a certain threshold, elevated ionic concentrations induce chromatin self-association, leading to chromatin phase separation into solid-like aggregates (liquid-solid phase separation, LSPS) that can be recovered in the pellet after centrifugation^41^. Recent studies have revealed that chromatin can also undergo liquid-liquid phase separation (LLPS) under specific solution conditions^42–45^. These distinct phase separation phenomena are induced by cation-mediated charge neutralization of DNA^44^ and are dependent on the presence of intrinsically disordered histone tail residues^42^. Notably, the presence of the linker histone reduces the ionic concentration required for chromatin condensate formation^42^.

**Fig. 5:**
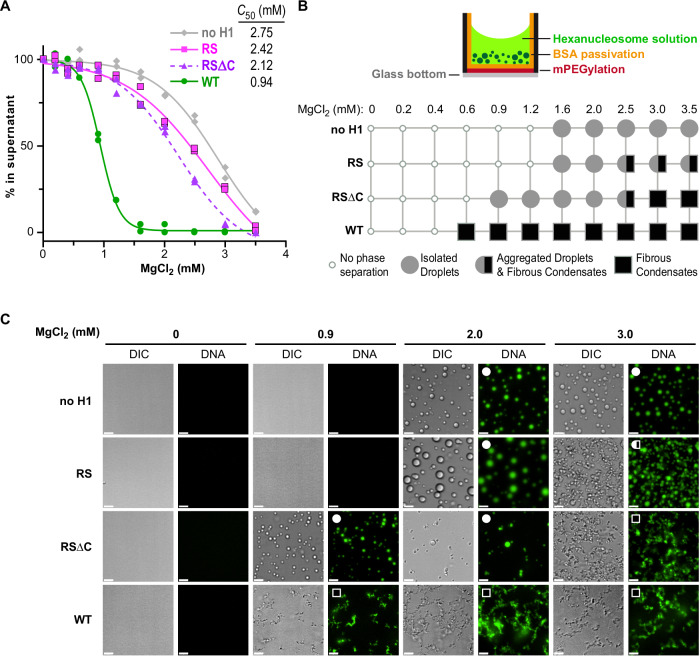
The RS mutation impairs chromatin phase separation. **A** Differential centrifugation analysis of 6 × 187 bp hexanucleosomes reconstituted in the absence or presence of WT or mutant forms of H1.4. The fraction of hexanucleosomes remaining in the supernatant, as determined by A_260nm_ measurements, is shown as a function of MgCl_2_ concentration. *C*_50_ is the Mg^2+^ concentration needed to induce 50% precipitation. Data represent measurements from two biological replicates. **B** *Top*. Schematic illustration of 384-well plate used for fluorescence microscopy imaging. *Bottom*. Phase diagram of hexanucleosome as a function of MgCl_2_ concentration. **C** Selected DIC and fluorescence microscopy images of hexanucleosomes stained with Vybrant™ DyeCycle™ Green Stain. Scale bar, 10 μm. Source data are available in the source data file.

We evaluated the impact of the RS mutation on the LSPS properties of H1.4-bound hexanucleosome arrays using differential centrifugation assays at varying MgCl_2_ concentrations. As expected, the presence of the linker histone lowered the MgCl_2_ concentration needed to pellet arrays (Fig. 5A). With WT H1.4, half of the arrays precipitated at 0.9-1 mM MgCl_2_, reaching complete precipitation at 1.6 mM. In contrast, arrays lacking the linker histone required approximately 2.7 mM MgCl_2_ to achieve 50% precipitation. Strikingly, RSΔC- and RS-bound arrays behaved like those lacking the linker histone, with half precipitation occurring at 2.1 and 2.4 mM MgCl_2_, respectively. This suggests that the loss of positive charge in the CTD of the RSΔC and RS mutants increases the MgCl_2_ concentration needed for DNA charge neutralization. Mass photometry confirmed these findings, showing that WT H1.4-bound arrays began to aggregate at 0.6 mM MgCl_2_, whereas arrays lacking H1.4 aggregated only at 2 mM MgCl_2_ (Supplementary Fig. 8). Arrays bound to the RS and RSΔC mutants behaved like linker histone-free arrays, with aggregation beginning at 1.6 mM MgCl_2_. Notably, the mass distribution profiles confirmed that arrays remained associated with H1.4 at these MgCl_2_ concentrations (Supplementary Fig. 8).

Next, we used fluorescence and differential interference contrast (DIC) microscopy to assess the impact of the RS mutation on the LLPS properties of nucleosome arrays as a function of MgCl_2_ concentration. As observed above, WT H1.4 promoted phase separation at low MgCl_2_ concentrations, initiating at 0.6 mM (Fig. 5B, C and Supplementary Fig. 9). Notably, only fibrous structures were observed across all MgCl_2_ concentrations tested. In contrast, H1.4-free arrays underwent phase separation at higher MgCl_2_ concentrations, starting at 1.6 mM, and formed distinct droplets that fused over time, confirming bona fide LLPS^42^ (Fig. 5B, C and Supplementary Fig. 9). Arrays bound to RSΔC initiated phase separation at higher MgCl_2_ concentrations than WT H1.4-bound arrays, but lower than H1.4-free arrays. RS-bound arrays underwent LLPS at a similar MgCl_2_ concentration as H1.4-free arrays. Both RS and RSΔC mutant arrays initially formed droplets that gradually transitioned into fibrous structures with increasing MgCl_2_ concentration.

Collectively, our LSPS and LLPS experiments reveal that replacement of WT H1.4 by the RSΔC and RS mutants shifts the phase-separation behavior of arrays towards that seen in the absence of linker histone, both by raising the MgCl_2_ concentration threshold required for a phase transition and by favoring LLPS over LSPS.

### The RS mutation induces a more open linker DNA conformation

**Fig. 6:**
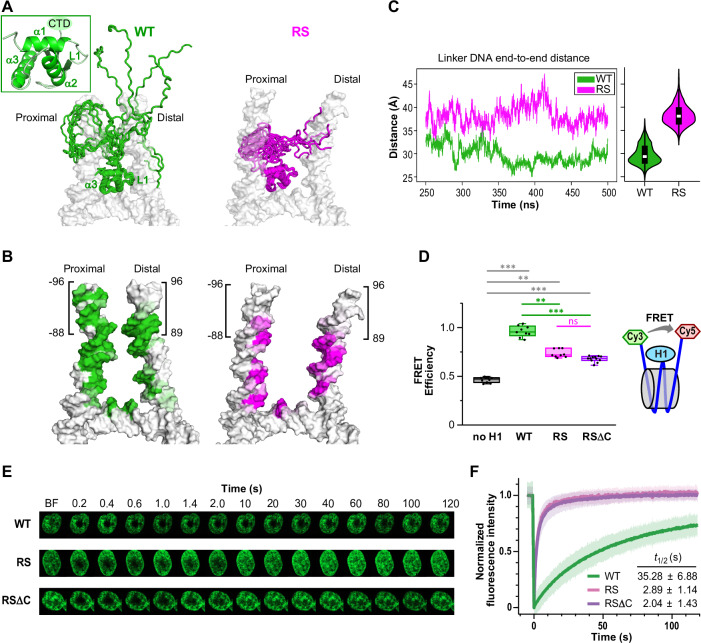
The RS mutation induces a more open nucleosome conformation and enhances H1.4 mobility in vivo. **A** MD simulation showing that the RS mutant H1.4 loses its interaction with the terminal regions of DNA linkers. Overlay of every 50^th^ ns configuration of WT (left) and RS mutant (right) H1.4 as extracted from the MD trajectories. Inset: H1.4 globular domain. **B** Contact imprints of WT (left) and RS mutant (right) H1.4 on nucleosomal and linker DNA. Darker colors indicate stronger interactions. Pronounced local contact differences are labeled by brackets and base indices. **C** *Left*. Time evolution of linker DNA end-to-end distances. *Right*. Violin plots displaying the distribution of end-to-end distances over the indicated time interval. Data were obtained from a single independent MD simulation (*n* = 1 trajectory) per setting (WT or RS). Distributions were calculated from the last 2500 frames of each trajectory (250–500 ns), collected at 100 ps intervals. Mean ± S.D. values are 29.72 ± 2.06 (WT) and 38.26 ± 2.17 (RS). Box plots display the median and the lower and upper quartiles, while violin plots display the minimum and maximum values. **D** Normalized FRET efficiency for Cy3-/Cy5-labeled nucleosomes in the absence or presence of the indicated H1.4 variant. Points represent individual FRET measurements (*n* = 3 biological replicates per condition, with 3–4 technical replicates per biological replicate). Box plots display the median, lower and upper quartiles, and minimum and maximum values. Two-sided Student’s *t* tests with Bonferroni correction yielded the following *p* values: WT vs no H1, 3.38 × 10⁻⁵; RS vs no H1, 0.00170; RSΔC vs no H1, 1.88 × 10⁻⁴; RS vs WT, 0.00921; RSΔC vs WT, 1.04 × 10⁻⁴; RS vs RSΔC, 0.201. *** *p* < 0.001; ** *p* < 0.01; ns, not significant. **E** FRAP experiment comparing the mobility of H1.4 variants. The leftmost image (BF) in each series was recorded before bleaching. **F** Time course of fluorescence in bleached area, showing mean intensity and S.D. Data were obtained from at least 40 cells per condition. Mean ± S.D. values of *t*_1/2_ are indicated. Source data are available in the source data file.

To better understand how the RS mutation affects chromatin structure and dynamics, we performed equilibrium molecular dynamics (MD) simulations of an H1.4-bound nucleosome and accelerated MD simulations of H1.4-bound DNA fragments. Specifically, we conducted 0.5 μs long all-atom equilibrium MD simulations in explicit solvent of the nucleosome bound to either the WT or RS mutant form of H1.4. Following a previous naming convention^46^, we designate the DNA linkers facing the α3 helix or the L1 loop of H1.4 as “proximal” and “distal”, respectively. Starting from the dyad-bound GH1 domain and elongated CTD configurations, the CTD of WT H1.4 gradually collapsed onto the two linker DNA arms, with a slight preference toward the proximal side (Fig. 6A and Supplementary Movie 1). While the RS CTD maintained this slight preference, its overall interactions with the linker DNA were substantially reduced due to de novo contacts of this CTD with itself (Fig. 6A and Supplementary Movie 2). The RS CTD rapidly aggregated onto itself even before binding the nucleosome, thereby leading to significantly reduced interactions with the terminal regions of both linker DNA arms (Fig. 6B and Supplementary Fig. 10). This drop in linker arm contacts is most pronounced at approximately base pair 88 and beyond (numbering from the nucleosomal dyad base pair as position 0), leading to more open nucleosome configurations. To quantify this difference in openness, we computed the distances between the near-terminal DNA base pairs on each linker arm for both the WT and RS nucleosomes. Time-dependent trajectories of this distance reveal a substantial loss of compaction of the RS nucleosome, with the mean distance between linker ends increasing from approximately 30 to 38 Å when WT H1.4 is replaced by the RS mutant (Fig. 6C). In agreement with these observations, the predicted DNA-binding affinity of the WT H1.4 was approximately 2 kcal/mol more favorable than that of the RS mutant (–13.10 ± 0.70 kcal/mol vs. –11.09 ± 0.40 kcal/mol; Supplementary Fig. 11), corresponding to a 26-fold increase in the dissociation constant (*K*_D_ = 0.58 nM vs. 15.2 nM). In complementary DNA pulling simulations involving only the linker histone and nucleosomal linker arms, the WT H1.4 CTD remained stably associated with both DNA arms over a 6 ns trajectory, whereas the RS mutant detached from one of the arms, further underscoring its reduced ability to bridge and stabilize the linker DNA (Supplementary Movies 3 and 4). Taken together, these findings indicate that the RS mutation weakens the CTD-DNA interactions and induces a considerably more open linker arm configuration in H1.4-bound nucleosomes.

To test this predicted increase in linker-arm separation, we performed fluorescence resonance energy transfer (FRET) analyses of H1.4-bound mononucleosomes. We used a 207 bp Widom 601 sequence, in which one strand was labeled on the 5’- and 3’-ends with Cy5 and Cy3 fluorophores, respectively, to reconstitute nucleosomes with either the WT or mutant forms of H1.4. We then performed FRET experiments to assess the linker DNA end-to-end distance, following an established protocol^47^. The presence of WT H1.4 yielded a robust FRET signal, whose intensity dropped to approximately 45% in the absence of H1.4 (Fig. 6D and Supplementary Fig. 12), consistent with the convergent and divergent linker arm configurations expected for linker histone-bound and unbound nucleosomes, respectively^9,10,47^. The RS and RSΔC mutants yielded weak FRET signals that were approximately 65% that of the WT. These results confirm that the mutants fail to bring the DNA linkers into close proximity, resulting in a more open linker-arm conformation.

### The RS mutation enhances H1.4 mobility in vivo

H1 binding to nucleosomes is stabilized by direct contacts with the nucleosomal dyad and both DNA linkers^7,9,34^. Conversely, a more open nucleosome conformation and greater flexibility of the linkers impairs the association with H1 (ref. ^48^). Accordingly, we hypothesized that the inability of the RS and RSΔC mutants to bring the DNA linker ends into close apposition would result in reduced nucleosome binding and decreased residence time within chromatin, leading to their increased mobility within cell nuclei. To test this hypothesis, we performed fluorescence recovery after photobleaching (FRAP) experiments. We exploited mouse embryonic stem (mES) cells that we recently developed, in which CRISPR/Cas9-based gene editing was used to replace endogenous H1.4 with photoactivatable GFP-tagged versions of either the WT, RS, or RSΔC H1.4 variants^49^. A small nuclear region was photobleached in single cells expressing GFP-tagged H1.4 and fluorescence images were recorded at defined intervals. The fluorescence recovery observed with WT H1.4 exhibited a half-life (*t*_1/2_) of ∼50 s (Supplementary Fig. 13A, B), consistent with previous reports^50–52^. In contrast, the *t*_1/2_ values for the RS and RSΔC mutants were reduced by a factor of approximately 7 and 20, respectively. To determine whether the presence of endogenous WT H1.4 affects the dynamics of the mutant proteins, we performed FRAP experiments in U2OS Flp-In cells expressing GFP-tagged WT, RS, or RSΔC H1.4 variants integrated at a defined genomic locus, a system that retains endogenous H1.4. WT H1.4 exhibited a fluorescence recovery half-life of ~35 s, while the RS and RSΔC mutants displayed markedly shorter half-lives of 2–3 s (Fig. 6E, F). These values are comparable to those observed in mES cells, indicating that the mutant proteins remain highly mobile even in the presence of endogenous WT H1.4. This suggests that their reduced chromatin residence time is an intrinsic property that is not substantially affected by coexisting WT protein. Together, these results demonstrate that the RS and RSΔC mutations substantially enhance H1.4 mobility in the nucleus, in line with our MD simulations and in vitro data showing more open and flexible DNA linker conformations.

### RS and RSΔC mutants impair chromatin compaction and phase separation in the presence of WT H1.4

**Fig. 7:**
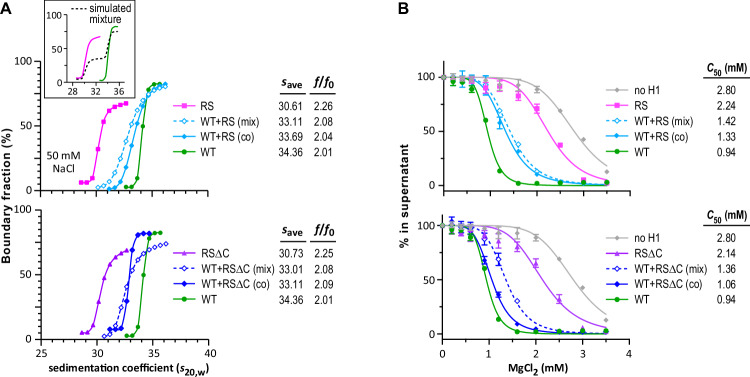
RS and RSΔC mutants perturb hexanucleosome behavior in the presence of WT H1.4. **A** Sedimentation velocity profiles of 6 × 187 bp hexanucleosome arrays in 50 mM NaCl. Arrays co-reconstituted (“co”) with an equimolar mixture of WT and either the RS or RSΔC mutant are indicated by solid cyan and blue curves, respectively. Arrays reconstituted separately with WT or mutant H1.4 and subsequently mixed in a 1:1 ratio (“mix”) are shown as dashed curves. The average sedimentation coefficient (*s*_ave_) and frictional ratio (*f*/*f*_0_) are indicated for the main species sedimenting between 20 and 40 S. The inset shows the theoretical sedimentation profile expected for a 1:1 mixture of WT and RS mutant arrays if the two species behaved as distinct, non-exchanging particles (dashed line). **B** Differential centrifugation analysis of hexanucleosomes reconstituted without H1 or in the presence of WT, mutant or mixed forms of H1.4. The fraction of hexanucleosomes remaining in the supernatant is plotted as a function of MgCl_2_ concentration. *C*_50_ denotes the Mg^2+^ concentration required for 50% precipitation. Data for WT and no-H1 samples are duplicated in the top and bottom panels to facilitate comparison. Data represent the mean and SD from three technical replicates. Source data are available in the source data file.

All RS patients identified to date are heterozygous for *H1-4*, expressing both mutant and WT H1.4 variants from different alleles. To model this heterozygous state in vitro, we reconstituted hexanucleosome arrays with an equimolar mixture of WT and either the RS or RSΔC mutant and assessed their degree of compaction by AUC (Fig. 7A). For both mutants, the mixed arrays displayed sedimentation profiles intermediate between WT-only and mutant-only arrays, indicating partial loss of compaction. Notably, the same intermediate behavior was observed when WT and mutant arrays were reconstituted separately and subsequently mixed in a 1:1 ratio. In the hypothetical case that WT-only and mutant-only arrays persisted as separate species, the post-mixed sample would be expected to exhibit a biphasic sedimentation profile (Fig. 7A, inset). Instead, a single well-defined boundary was observed, indicating a hydrodynamically homogeneous population consistent with rapid exchange of WT and mutant H1.4 between arrays. Thus, whether mixed during reconstitution or combined post-assembly, the simultaneous presence of WT and mutant H1.4 leads to arrays that exhibit reduced compaction compared to WT-only arrays.

To further characterize these mixed arrays, we examined their LSPS behavior using differential centrifugation (Fig. 7B). The mixed WT/RS arrays displayed intermediate LSPS behavior relative to the WT and RS mutant arrays, with nearly identical results for co-reconstituted and post-assembly mixed arrays. The WT/RSΔC arrays showed a similar trend, although, for reasons not clear, the co-reconstituted sample precipitated at a lower Mg^2+^ concentration than the post-assembly mixed sample. Despite this minor variation, both RS and RSΔC consistently attenuated the ability of WT H1.4 to promote phase separation of nucleosome arrays. Together, these findings demonstrate that the RS and RSΔC mutants antagonize WT H1.4 function, pointing to a dominant negative effect on chromatin compaction and phase separation.

## Discussion

Rahman syndrome is associated with C-terminal frameshift mutations in linker histone H1.4, yet the mechanisms linking these mutations to pathogenesis remain unclear. In this study, we used nucleosomes and nucleosome arrays to investigate how a prototypical RS-associated mutation, c.430dupG, affects chromatin structure and dynamics. Hydroxyl radical footprinting revealed that both WT and mutant H1.4 provide similar protection at the nucleosome dyad, confirming correct positioning of the H1.4 globular domain, but that the mutant increases linker DNA accessibility. MD simulations of H1.4-bound nucleosomes rationalized this observation by showing that the RS mutation leads to a more compact CTD, facilitated by electrostatic interactions between its basic residues and the acidic C-terminal motif. Consequently, compared to WT H1.4, the mutant CTD associates with a shorter, core-proximal segment of the distal DNA linker, making it less effective at drawing the two linker arms together. Binding-energy calculations indicated that the RS mutant interacts with nucleosomal DNA approximately 25-fold more weakly than the WT H1.4, while DNA-pulling simulations showed that it readily detaches from one linker arm, underscoring its reduced ability to bridge and stabilize the linker DNA. In agreement with the MD and DNA-pulling simulations, FRET assays confirmed that the end-to-end linker DNA distances in RS-bound nucleosomes were increased relative to WT.

The loss of CTD-mediated electrostatic neutralization and the more divergent DNA linker configuration deduced from the MD and FRET analyses are expected to promote a more extended and flexible nucleosome array conformation. Our AUC and SAXS experiments confirmed these structural changes. The convergence of WT and mutant array behavior at higher Mg^2+^ concentrations in these experiments is consistent with Mg^2+^ compensating for the reduced charge screening of the H1.4 mutants through nonspecific electrostatic shielding. Cryo-EM analysis further revealed that arrays bound to the RS mutant are impaired in their ability to form compact structures with regular nucleosome stacking. This deficiency correlates with a reduced propensity to undergo LLPS and LSPS, leading to phase separation behavior resembling that of H1-free arrays. Consistent with a more open, flexible chromatin architecture, our FRAP experiments in vivo demonstrated markedly higher mobility for the RS mutant compared to WT H1.4, indicating that RS mutations lead to a more transient and dynamic association of H1.4 with chromatin.

RS is an autosomal dominant disorder in which all affected individuals reported to date are heterozygous at the *H1-4* locus and therefore express both mutant and WT H1.4. In mice, knockout of a single H1 subtype does not cause a pronounced phenotype or significantly affect development, which only becomes compromised when multiple isoforms are inactivated^53–56^. Given this functional redundancy, the inability of the WT H1.4 allele or other H1 isoforms to compensate for the RS mutation has been interpreted as evidence for a dominant-negative or gain-of-function effect^2^. In line with this view, nucleosome arrays reconstituted with equimolar WT and mutant H1.4 exhibited sedimentation and phase-separation properties intermediate between those of WT-only and mutant-only arrays, consistent with a dominant-negative effect in which incorporation of the mutant protein compromises chromatin compaction in mixed WT/mutant assemblies^57^. A canonical example of such behavior is provided by dimeric transcription factors, where dominant-negative mutations preserve dimerization and DNA binding but disrupt the transcriptional activation domain, such that WT/mutant heterodimers bind DNA normally yet are defective in transcriptional activation^58^. Analogously, RS H1.4 retains the ability to bind nucleosomes but forms mixed WT/RS chromatin assemblies that are compromised for chromatin compaction and phase separation. While our data support a dominant-negative mode of action, we cannot exclude the possibility that RS-associated mutations may additionally exert gain-of-function effects in vivo that are not captured by the experimental approaches used in this study.

Our data show that the RS mutation contributes to a more relaxed chromatin structure in two ways. First, when bound to nucleosomes, the RS variant promotes an extended and flexible chromatin conformation with phase separation properties resembling those of H1-free chromatin. Second, the RS variant displays increased mobility within the nucleus, indicating a shorter chromatin residence time. While nucleosomal sites transiently made vacant by the dissociation of the RS variant could in principle allow WT H1.4 or other H1 isoforms to rebind and thereby mitigate the effects of the mutation, a nucleosome-bound RS variant causing chromatin to mimic the H1-free state would competitively inhibit such binding, preventing the rescue of chromatin structure and function. Thus, the adverse effect of the RS mutant on chromatin compaction and phase separation under conditions mimicking the heterozygous situation is more likely attributable to the competition between WT and mutant H1.4 for nucleosomal binding sites rather than to the reduced chromatin residence time of the mutant.

Interestingly, the RSΔC mutant that lacks the acidic C-terminal motif behaved nearly identically to the RS mutant across multiple assays. Specifically, DNA footprinting and FRET experiments showed both mutants to induce a similar increase in linker DNA accessibility and in linker arm divergence; AUC and SAXS experiments revealed the same extended and flexible nucleosome array structures; and FRAP analyses demonstrated a similar elevated mobility within cell nuclei. These findings indicate that the observed changes in chromatin structure and dynamics, at least under the in vitro conditions tested here, are primarily driven by the loss of the WT C-terminal sequence caused by the frameshift mutation, rather than by the acquisition of the acidic C-terminal motif. In contrast, LSPS and LLPS assays revealed distinct behavior between the two mutants, with the RSΔC mutant allowing phase separation at lower MgCl_2_ concentrations than the RS variant. These findings imply that, while efficient nucleosome array compaction and phase separation rely on the WT C-terminal sequence, the C-terminal acidic motif generated by the RS mutation further hinders the latter process.

Taken together, our data show that an RS-associated mutation promotes extended and open nucleosome array structures with phase separation properties resembling those of linker histone-free arrays. These findings suggest that, in vivo, the presence of an RS variant could significantly disrupt 3D chromatin organization. Given the relative abundance of H1.4 in cells^16,56,59^, such structural changes could propagate genome-wide, affecting essential nuclear processes and ultimately disturbing nuclear homeostasis. Indeed, fibroblasts isolated from RS patients showed severe alterations in nuclear morphology, mitotic defects, aneuploidy, nucleolar instability, and perturbed cell cycle progression, as well as distinct genome-wide DNA methylation profiles^2^. In conclusion, while further research is needed to fully elucidate the molecular etiology of RS, our findings provide key insights into the initial chromatin alterations that likely underlie disease development.

## Methods

### Purification of linker histone H1.4

The codon-optimized human H1.4 gene variants, including WT, the Rahman Syndrome mutant (RS) (c.430dupG), and the RS mutant lacking the negatively charged 38-amino acids (RSΔC), all carrying a C-terminal 6×His tag, were synthesized by Eurofins Genomics. These genes were subsequently cloned into a pGEX-6P-3 vector using the BamHI and XhoI sites. Rosetta™(DE3) Competent Cells (Novagen) were transformed with 200 ng of plasmid DNA and grown on an agar plate by re-plating a single colony on LB agar plates supplemented with ampicillin (100 µg/ml) and chloramphenicol (34 µg/ml) overnight at 37 °C. The bacterial lawn on the entire plate was suspended in 1 L of LB medium containing ampicillin (50 µg/mL), and chloramphenicol (34 µg/mL) and grown at 37 °C until reaching an OD600 of 0.5–0.6. Induction was performed with 1 mM Isopropyl-P-D-thiogalactopyranoside (IPTG), followed by further incubation at 16 °C for 18 h. Following induction, bacterial cells were pelleted by centrifugation at 5000 *g* for 20 min at 4 °C, suspended in buffer A (20 mM Tris-HCl pH 8.0, 1 M NaCl, 10% glycerol, 0.1% β-mercaptoethanol, 0.2% Triton X-100, 10 mM imidazole and 1 mM PMSF) and disrupted by sonication. After centrifugation at 27,000 *g* for 20 min at 4 °C, the soluble lysate was incubated with NiNTA resin (Complete His-Tag Purification Resin, Roche) equilibrated with buffer B (20 mM Tris-HCl pH 8.0, 1 M NaCl, 10% glycerol, 0.1% β-mercaptoethanol, 0.2% Triton X-100, 20 mM imidazole, and 1 mM PMSF) for 2 h with end-over-end mixing at 4 °C. The NiNTA resin was then transferred to Econo-Pac® Chromatography Columns (Bio-Rad) and washed with at least 20 column volumes of buffer B. Elution was carried out with buffer C (20 mM Tris-HCl pH 8.0, 1 M NaCl, 10% glycerol, and 0.1% β-mercaptoethanol) containing a linear gradient of imidazole from 25 mM to 500 mM. The fractions containing the recombinant protein were pooled and dialyzed against Cleavage Buffer (50 mM Tris-HCl, pH 7.0, 150 mM NaCl, 1 mM EDTA, and 1 mM dithiothreitol) before cleavage using PreScission Protease (Cytiva) at 4 °C overnight. Complete cleavage was confirmed by SDS-PAGE with Coomassie Brilliant Blue staining. The cleaved linker histones were then dialyzed against buffer D (20 mM Tris-HCl pH 8.0, 600 mM NaCl, and 1 mM dithiothreitol) at 4 °C. The cleaved recombinant proteins were applied to Glutathione Sepharose 4 Fast Flow (Cytiva) equilibrated with buffer D and washed with 10 column volumes of buffer D. The flow-through and wash fractions were pooled, and the final imidazole concentration was adjusted to 10 mM. Subsequently, the mixture was applied to Econo-Pac® Chromatography Columns containing NiNTA resin equilibrated with buffer D with 20 mM imidazole. The NiNTA resin was washed with 20 column volumes of buffer D (20 mM imidazole) and eluted using buffer D containing a linear gradient of imidazole from 25 mM to 500 mM. Fractions containing the protein of interest were collected, dialyzed against buffer D containing 20% glycerol, and stored at −80 °C in single-use aliquots.

### Human core histone purification

N-terminally His-tagged human core histones H2A, H2B, and H3 were expressed from a pHCE vector in *E. coli* BL21(DE3) cells, and human core histone H4 was expressed from a pET15b vector in *E. coli* JM109(DE3) cells in the absence of T7 RNA polymerase by omitting IPTG, which induces T7 RNA polymerase production. All histones were purified from the inclusion bodies under denaturing conditions as described previously^34^. Briefly, 200 ng of plasmid was transformed into the respective *E. coli* strains and incubated overnight at 37 °C. 10 colonies were inoculated into 2 L of LB medium containing 50 µg/ml ampicillin in a 5 L flask and incubated overnight at 37 °C with shaking at 200 rpm. Following incubation, the bacterial cells were pelleted by centrifugation at 5000 *g* for 20 min at 4 °C. Bacterial cells were resuspended in buffer A (50 mM Tris-HCl pH 8.0, 500 mM NaCl, 1 mM phenylmethylsulfonyl fluoride (PMSF), and 5% glycerol), disrupted by sonication, and then centrifuged at 27,000 *g* for 20 min at 4 °C. The pellet containing His-tagged histones in insoluble forms was resuspended in buffer A supplemented with 7 M guanidine hydrochloride and subjected to centrifugation at 27,000 *g* for 20 min at 4 °C. The supernatant containing His-tagged histones was then incubated with NiNTA resin (Complete His-Tag Purification Resin, Roche) equilibrated with buffer B (50 mM Tris-HCl pH 8.0, 500 mM NaCl, 6 M urea, 5 mM imidazole, and 5% glycerol) for 1 h with end-over-end mixing at 4 °C. NiNTA resin was transferred into Econo-Pac® Chromatography Columns (Bio-Rad), washed with buffer B, and subsequently eluted with a linear gradient of imidazole ranging from 5 mM to 500 mM in buffer B. Fractions containing eluted histones were dialyzed overnight at 4 °C against buffer C (5 mM Tris-HCl pH 7.5, 2 mM β-mercaptoethanol). The N-terminal His-tag was cleaved using thrombin protease (Cytiva) for 3–5 h at 4 °C. Following protease treatment, the histones were dialyzed against buffer D (20 mM sodium acetate pH 5.2, 200 mM NaCl, 5 mM β-mercaptoethanol, 1 mM EDTA, and 6 M urea), and the untagged histone was further purified by Resource S cation exchange column chromatography (Cytiva) using buffer D containing a linear gradient of NaCl from 200 to 900 mM. Eluted fractions containing pure histones were pooled and stored at −80 °C.

### Preparation of histone tetramers and dimers

Histone tetramers and dimers were prepared by mixing equimolar ratios of histone H3 with histone H4 and histone H2A with H2B, respectively, and folded by dialysis against Histone-Folding (HF) buffer (2 M NaCl, 10 mM Tris pH 7.4, 1 mM EDTA pH 8, and 10 mM β-mercaptoethanol) at 4 °C. The folded tetramer and dimer were further purified through Superose 6 Prep grade XK 16/70 size-exclusion column chromatography using HF buffer. The fractions containing purified tetramers and dimers were collected together and subsequently dialyzed against HF buffer containing 20% glycerol and stored at −20 °C.

### Preparation of DNA for hexanucleosome arrays and dinucleosomes

The 6 × 187 bp, 6 × 197 bp and 2 × 197 bp 601 DNA fragments were prepared as previously described^9,34^. The pGEM-T Easy vector containing three copies of the 6 × 187 bp 601 DNA sequence was amplified in *E. coli* DH5α. The 6 × 187 bp DNA sequence was excised from the vector using EcoRV, followed by phenol-chloroform extraction and ethanol precipitation. Subsequently, the 6 × 187 bp fragment was separated from the linearized vector by preparative-scale 1% agarose gel electrophoresis, eluted by electroelution and further purified through phenol-chloroform extraction and ethanol precipitation. The 2 × 197 bp and 6 × 197 bp 601 DNA fragments were obtained from a pGEM-T plasmid harboring a 12 × 197 bp 601 array by digestion with EcoRI and either BamH1 or BglII, respectively, and purified from the linearized vector by agarose gel electrophoresis. The DNA sequences are as follows:

#### 6 × 187 bp

ATCGCTGTTCAATACATGCACAGGATGTATATATCTGACACGTGCCTGGAGACTAGGGAGTAATCCCCTTGGCGGTTAAAACGCGGGGGACAGCGCGTACGTGCGTTTAAGCGGTGCTAGAGCTGTCTACGACCAATTGAGCGGCCTCGGCACCGGGATTCTCCAGGGCGGCCGCGTATAGGGTCTCGGGGCTGTTCAATACATGCACAGGATGTATATATCTGACACGTGCCTGGAGACTAGGGAGTAATCCCCTTGGCGGTTAAAACGCGGGGGACAGCGCGTACGTGCGTTTAAGCGGTGCTAGAGCTGTCTACGACCAATTGAGCGGCCTCGGCACCGGGATTCTCCAGGGCGGCCGCGTATAGGGTCTCGGGGCTGTTCAATACATGCACAGGATGTATATATCTGACACGTGCCTGGAGACTAGGGAGTAATCCCCTTGGCGGTTAAAACGCGGGGGACAGCGCGTACGTGCGTTTAAGCGGTGCTAGAGCTGTCTACGACCAATTGAGCGGCCTCGGCACCGGGATTCTCCAGGGCGGCCGCGTATAGGGTCTCGGGGCTGTTCAATACATGCACAGGATGTATATATCTGACACGTGCCTGGAGACTAGGGAGTAATCCCCTTGGCGGTTAAAACGCGGGGGACAGCGCGTACGTGCGTTTAAGCGGTGCTAGAGCTGTCTACGACCAATTGAGCGGCCTCGGCACCGGGATTCTCCAGGGCGGCCGCGTATAGGGTCTCGGGGCTGTTCAATACATGCACAGGATGTATATATCTGACACGTGCCTGGAGACTAGGGAGTAATCCCCTTGGCGGTTAAAACGCGGGGGACAGCGCGTACGTGCGTTTAAGCGGTGCTAGAGCTGTCTACGACCAATTGAGCGGCCTCGGCACCGGGATTCTCCAGGGCGGCCGCGTATAGGGTCTCGGGGCTGTTCAATACATGCACAGGATGTATATATCTGACACGTGCCTGGAGACTAGGGAGTAATCCCCTTGGCGGTTAAAACGCGGGGGACAGCGCGTACGTGCGTTTAAGCGGTGCTAGAGCTGTCTACGACCAATTGAGCGGCCTCGGCACCGGGATTCTCCAGGGCGGCCGCGTATAGGGTGAT

#### 6 × 197 bp

GAATTCAGGTCGCTGTTCAATACATGCACAGGATGTATATATCTGACAACAGCGTGGAGACTAGGGAGTAATCCCCTTGGCGGTTAAAACGCGGGGGACAGCGCGTACGTGCGTTTAAGCGGTGCTAGAGCTTGCTACGACCAGACGAGCGGCCTCGGCACCGGGATTCTCCAGGGCGGCCGCGTATAGGGTCCATCAGTACTAGGTCTTAAGTCAATACATGCACAGGATGTATATATCTGACAGAGGCGTGGAGACTAGGGAGTAATCCCCTTGGCGGTTAAAACGCGGGGGACAGCGCGTACGTGCGTTTAAGCGGTGCTAGAGCTTGCTACGACCACGAGAGCGGCCTCGGCACCGGGATTCTCCAGGGCGGCCGCGTATAGGGTCCATCAGTACTAGGTGGATCCTCAATACATGCACAGGATGTATATATCTGACATTCGCGTGGAGACTAGGGAGTAATCCCCTTGGCGGTTAAAACGCGGGGGACAGCGCGTACGTGCGTTTAAGCGGTGCTAGAGCTTGCTACGACCATCGGAGCGGCCTCGGCACCGGGATTCTCCAGGGCGGCCGCGTATAGGGTCCATCAGTACTAGGTCTTCGAACAATACATGCACAGGATGTATATATCTGACACGTTACTGGAGACTAGGGAGTAATCCCCTTGGCGGTTAAAACGCGGGGGACAGCGCGTACGTGCGTTTAAGCGGTGCTAGAGCTTGCTACGACCAATTCCACGGCCTCGGCACCGGGATTCTCCAGGGCGGCCGCGTATAGGGTCCATCAGTACTAGGTCATATGTCAATACATGCACAGGATGTATATATCTGACACGTAGTTGGAGACTAGGGAGTAATCCCCTTGGCGGTTAAAACGCGGGGGACAGCGCGTACGTGCGTTTAAGCGGTGCTAGAGCTTGCTACGACCAATTTTCCGGCCTCGGCACCGGGATTCTCCAGGGCGGCCGCGTATAGGGTCCATCAGTACTAGGTGCACGTTCAATACATGCACAGGATGTATATATCTGACACGTCTATGGAGACTAGGGAGTAATCCCCTTGGCGGTTAAAACGCGGGGGACAGCGCGTACGTGCGTTTAAGCGGTGCTAGAGCTTGCTACGACCAATTAGTCGGCCTCGGCACCGGGATTCTCCAGGGCGGCCGCGTATAGGGTCCATCAGTACTAGGTAGATCT

#### 2 × 197 bp

GAATTCAGGTCGCTGTTCAATACATGCACAGGATGTATATATCTGACAACAGCGTGGAGACTAGGGAGTAATCCCCTTGGCGGTTAAAACGCGGGGGACAGCGCGTACGTGCGTTTAAGCGGTGCTAGAGCTTGCTACGACCAGACGAGCGGCCTCGGCACCGGGATTCTCCAGGGCGGCCGCGTATAGGGTCCATCAGTACTAGGTCTTAAGTCAATACATGCACAGGATGTATATATCTGACAGAGGCGTGGAGACTAGGGAGTAATCCCCTTGGCGGTTAAAACGCGGGGGACAGCGCGTACGTGCGTTTAAGCGGTGCTAGAGCTTGCTACGACCACGAGAGCGGCCTCGGCACCGGGATTCTCCAGGGCGGCCGCGTATAGGGTCCATCAGTACTAGGTGGATCC

### Reconstitution of 6 × 187 bp hexanucleosome arrays

The 6 × 187 bp 601 hexanucleosomal array was reconstituted using purified human core histones by a well-established salt gradient dialysis method^34^. The 6 × 187 bp 601 DNA was mixed in HF buffer with human H3-H4 tetramers and H2A-H2B dimers at molar ratios corresponding to one histone octamer per 187 bp repeat. The mixtures were transferred into dialysis tubing and reconstitution was performed by dialysis against a buffer whose NaCl concentration was gradually reduced to 0.6 M using a peristaltic pump. Full-length WT H1.4, the RS mutant or the RSΔC mutant was added to the hexanucleosome in an equimolar ratio relative to the core histone octamer at 0.6 M NaCl. The samples were then dialyzed in 10 mM NaCl, 10 mM Tris-HCl pH 7.4, 0.25 mM EDTA, and their quality was verified by native agarose gel electrophoresis. For array mixing experiments, hexanucleosome arrays containing WT and mutant H1.4 were prepared using two approaches. In co-reconstitution, WT H1.4 and either RS or RSΔC were added simultaneously during array assembly at a 1:1 molar ratio, corresponding to 0.5 equivalents of each per core histone octamer (providing one total H1.4 per octamer), at 0.6 M NaCl. The samples were then dialyzed into 10 mM NaCl, 10 mM Tris-HCl pH 7.4 and 0.25 mM EDTA. In post-assembly mixing, arrays reconstituted separately with WT or mutant H1.4 were combined at a 1:1 ratio based on DNA concentration. Uncropped and unprocessed scans of gels are provided in the Source Data file.

### Preparation of dinucleosomes and hexanucleosomes for hydroxyl radical footprinting

The purified 2 × 197 bp and 6 × 197 bp tandem DNA fragments were radiolabeled with ^32^P at the 3’-end by Klenow fill-in, and nucleosome reconstitution was carried out as in ref. ^34^ using the following procedure. 500 ng of P-labeled DNA was combined with 9.5 μg of chicken erythrocyte carrier DNA fragments (300–400 bp). For stoichiometric calculations, the total DNA present was expressed in equivalents of 197-bp nucleosome positioning sites. Human H3-H4 tetramers and H2A-H2B dimers were then added in HF buffer at stoichiometries corresponding to one histone octamer per equivalent positioning site. The mixtures were transferred to dialysis tubing, and nucleosome reconstitution was performed by gradually reducing the salt concentration to 0.6 M NaCl using a peristaltic pump. Wild-type H1.4, the RS mutant or the RSΔC mutant was added to the dinucleosomes or hexanucleosomal arrays at a 1:1 molar ratio relative to the core histone octamer at 0.6 M NaCl. Subsequently, samples were dialyzed into a low-salt buffer (10 mM NaCl, 10 mM Tris-HCl pH 7.4, 0.25 mM EDTA), and their quality was verified by native agarose gel electrophoresis.

### Hydroxyl radical footprinting analysis

Hydroxyl radical footprinting of dinucleosomes and hexanucleosomal arrays was performed as previously described^9,34^. Footprinting reactions (24 μl) contained 400 ng of radiolabeled nucleosomes in the unbound state or bound to linker histones H1.4 (WT, RS and RSΔC). The hydroxyl radicals were generated by mixing 4 μl of 2 mM FeAmSO_4_/4 mM EDTA, 0.1 M ascorbate and 0.12% H_2_O_2_ together in a drop on the side of the reaction tube, followed by rapid mixing with the reaction solution. After a 2-min digestion with hydroxyl radicals, the reaction was stopped by adding 100 μl of stop solution (0.1% SDS, 25 mM EDTA, 1% glycerol and 100 mM Tris, pH 7.4), and the DNA was purified by phenol/chloroform extraction and ethanol/glycogen precipitation. The denatured DNA samples were run on an 8% denaturing polyacrylamide gel in 1X TBE buffer. The gels were dried, exposed overnight and imaged using the Typhoon FLA 9500 biomolecular imager (Cytiva). Gel scans were analyzed using ImageQuant TL software (Cytiva).

### Analytical ultracentrifugation

Hexanucleosomes bound to linker histones H1.4 (WT, RS and RSΔC) were prepared as described above and dialyzed overnight against a buffer containing (i) 5 mM Tris-HCl pH 7.4, 0.25 mM EDTA and either 50 or 90 mM NaCl or (ii) 5 mM Tris-HCl pH 7.4 and either 0.35, 0.5 or 0.6 mM MgCl_2_. The hexanucleosome concentration ranged from 15 μg/mL to 36 μg/mL. Sedimentation velocity experiments were performed on a Beckman XL-I analytical ultracentrifuge equipped with an AN-50 TI rotor (Beckman Coulter, Brea, USA) at 20 °C, using 450 μL samples loaded into two channel 12 mm path-length centerpieces with Sapphire windows (Nanolytics). The absorbance at 260 nm was measured in continuous scan mode during sedimentation at 18,000 rpm. Data were processed using Redate software (v. 1.0.1) and analyzed in terms of *c(s)* distributions using SEDFIT (v. 16.36) and Gussi (v. 1.4.2). *s*_20, W_ values (sedimentation coefficient corrected for water at 20 °C) were calculated with a partial specific volume of 0.622 ml/g for chromatin, and solvent density and viscosity were calculated using SEDNTERP. The *c(s*_20w_) distributions were then converted into percentage boundary fractions, and the average sedimentation coefficients (*s*_ave_) were determined at the boundary midpoint. The frictional ratio *f/f*_0_ was derived from *s*_ave_ in the Svedberg equation.

### Small-angle X-ray scattering

Hexanucleosomes bound to linker histones H1.4 (WT, RS and RSΔC) were prepared as described above and dialyzed overnight against a buffer containing (i) 5 mM Tris-HCl pH 7.4, 0.25 mM EDTA and either 50 or 90 mM NaCl or (ii) 5 mM Tris-HCl pH 7.4 and either 0.35, 0.5 or 0.6 mM MgCl_2_. The final concentration of the samples tested at 50 and 90 mM NaCl or at 0.35, 0.5 and 0.6 mM MgCl_2_ was in the range of 15–45 μg/ml (DNA concentration). The SAXS data were collected at an X-ray energy of 12.5 keV at the ESRF beamline BM29. Scattered X-rays were recorded on a Pilatus 1M detector (Dectris) at a distance of 2.879 m from the sample. Samples were automatically loaded into a vacuum-mounted quartz capillary of 1.8 mm in diameter. 40–50 μL were loaded for each sample and 10 frames of 1 s duration were collected at 20 *°*C. The samples constantly flowed through the capillary during measurement to minimize the effect of radiation damage. Processing of individual frames, including azimuthal integration to obtain the one-dimensional scattering curve, was performed using a processing pipeline within the EDNA framework^60^. Primary data reduction was performed using the program PRIMUS^61^. Time frames were combined, excluding frames affected by aggregation due to radiation damage, to give the average scattering curve for each measurement. The average scattering from the buffer alone, measured before and after each sample, was used for the background subtraction. Model-independent scattering parameters (*R*_g_ and *D*_max_) were determined using PRIMUS. The Fourier transform was calculated using the program GNOM^62^. Scattering, density modification curves and Kratky plots were produced with the program ScÅtter (https://bl1231.als.lbl.gov/scatter/).

Ab initio models for each sample were calculated using the graphical interface program *BioXTAS RAW* 4^63^. For each input data, 15 bead reconstructions were calculated with DAMMIF^64^ in slow mode calculation. These models were further averaged and filtered with DAMAVER^65^ and finally refined with DAMMIN^66^. The final ab initio models were visualized using PyMOL (The PyMOL Molecular Graphics System, Version 2.5.5 Schrödinger, LLC.). All of the above mentioned software for SAXS analysis and visualization was compiled by SBGrid^67^.

### Mass photometry

Hexanucleosomes bound to linker histones H1.4 (WT, RS and RSΔC) were prepared as described above, dialyzed overnight against a buffer containing 5 mM Tris-HCl pH 7.4, 10 mM NaCl, and then concentrated to 200 μg/ml (DNA concentration). The hexanucleosomal arrays were condensed by intermittent addition of MgCl_2_ to adjust the concentration to 0, 0.2, 0.4, 0.6, 0.9, 1.2, 1.6, and 2.0 mM. Samples were collected at each ionic condition, and the final concentration of the samples was maintained at 200 μg/ml (DNA concentration) by supplementation with two-fold concentrated hexanucleosomes. For hexanucleosomes reconstituted without H1, the buffer was adjusted to 2.5 and 3.0 mM MgCl_2_. To perform the mass photometry experiment, a 20 μl drop of each individual sample at a final concentration of approximately 10 μg/ml (20-fold dilution of the initial concentration) was placed on a coverslip on top of the objective of a Refeyn One^MP^ instrument. Refeyn Acquire^MP^ software was used to record a movie of the particles landing on the coverslip for one minute using a large field of view. Each movie was then analyzed using Refeyn Discover^MP^ software, which calculates the molecular weight distribution of each sample using a predetermined calibration curve. The mass distribution histograms of the samples were treated and overlaid for comparison purposes using the same software.

The mass distribution of particles observed for the H1-free hexanucleosome array was fitted as a population mixture model comprising free DNA and nucleosome arrays containing between one and six nucleosomes (Supplementary Fig. 4A). To account for the slight positive skew (right-hand tail) observed in the experimental distribution, each species was represented by an exponentially modified Gaussian (EMG) function, widely used to model asymmetric peak shapes in analytical techniques such as chromatography and mass spectrometry^68,69^. The EMG function was defined as

*f*(*m*) = (1/(2*τ*)) · exp[0.5(*σ*/*τ*)² – (*m* – *μ*)/*τ*] · erfc[(*σ*/*τ* – (*m* – *μ*)/*σ*)/√2], where *μ* is the Gaussian mean, *σ* the standard deviation, *τ* the exponential time-scale parameter, and erfc the complementary error function. To prevent overfitting, all species were constrained to share a common distribution shape characterized by a single global σ and *τ*, each expressed as a fraction of the mean mass of the corresponding species. Thus, only these two fractional parameters, together with the overall mass of the full hexanucleosome and the amplitudes of the seven component distributions, were fitted (ten fitted parameters in total). The masses of all other species were determined automatically by fixing the histone octamer mass at 109.7 kDa. Both fractional parameters (*σ*, *τ*) were less than 5% of the corresponding mean mass.

The mass and H1 occupancy values shown in Supplementary Fig. 4B were determined as follows. The mean particle mass within each primary peak (shaded area) was calculated as the intensity-weighted expectation value, <*m* > = ∫ *m*·*I*(*m*)*dm*/∫ *I*(*m*)*dm*, where *I*(*m*) is the measured signal (counts). The fraction of array particles in the primary peak was obtained as the ratio of the peak area to the total area under the distribution between 600 and 2500 kDa (the mass range corresponding to all particles containing full-length DNA). The number of incorporated H1 molecules was derived by dividing the mean mass shift (ΔMass) relative to the H1-free array by the molecular mass of the respective H1.4 variant. The mean H1 occupancy (H1-to-nucleosome ratio) was then estimated by comparing this value with the mean number of nucleosomes per array (5.67) determined for the primary peak of the H1-free array.

### Differential centrifugation assay

Hexanucleosome arrays bound to linker histones H1.4 (WT, RS and RSΔC) or unbound hexanucleosomes were prepared as described above, dialyzed overnight against a buffer containing 5 mM Tris-HCl pH 7.4, 10 mM NaCl, and subsequently concentrated to 200 μg/ml (DNA concentration). The hexanucleosomal arrays were condensed by intermittent addition of MgCl_2_ to adjust the concentration to 0, 0.2, 0.4, 0.6, 0.9, 1.2, 1.6, 2.0, 2.5, 3.0, and 3.5 mM. Samples were collected at each ionic condition, and the final concentration of the samples was maintained at 200 μg/ml (DNA concentration) by supplementing with a two-fold concentrated hexanucleosomes. The samples were incubated at 20–23 °C for 15 min, centrifuged in an Eppendorf tube at 13,000 *g* for 3 min, and the absorbance of the supernatant was measured at 260 nm. Data were expressed as the percent of initial hexanucleosomal array absorbance present in the supernatant against the cation concentration in the solution.

### Preparation of 384-well microscopy plates

Microscopy plates were prepared as previously described^42,70^. Briefly, microscopy plates with high-performance #1.5 cover glass (product No. P384-1.5H-N, Cellvis) were treated with 5% Hellmanex (Fisher) at 37 °C for 4 h and then extensively rinsed with MilliQ water. Subsequently, the plates were treated with 1 M NaOH for 1 h and then rinsed with MilliQ water, followed by incubation with 25 mg/mL mPEG5K-silane (SIGMA JKA3037) in 95% ethanol overnight at room temperature. Plates were then rinsed with 95% ethanol and MilliQ water before being dried and sealed with adhesive PCR plate foils (Thermo). Right before use, the phase glasses of the microscopy plates were passivated by 100 mg/mL BSA for 30 min and then rinsed with MilliQ water.

### Preparation of phase separation samples

Hexanucleosome array samples were prepared as described above, dialyzed overnight against 10 mM Tris-HCl pH 7.4 and 10 mM NaCl buffer and then concentrated. Hexanucleosome array samples were equilibrated in 1X phase separation buffer (10 mM Tris, pH 7.4, 10 mM NaCl, 2 mM DTT, 0.05 mg/mL BSA, 2.5% [w/v] glycerol, 1 μg/mL Glucose Oxidase [SIGMA cat. no. G2133], 175 ng/mL Catalase [SIGMA cat. no. C1345], 2 mM Glucose and 0.25 μM DyeCycle stain [ThermoFisher cat. No. V35004]) to achieve a final sample concentration of 200 μg/ml (DNA concentration). Phase separation was induced by intermittently adding 1X phase separation buffer containing MgCl_2_ to adjust the concentration to 0, 0.2, 0.4, 0.6, 0.9, 1.2, 1.6, 2.0, 2.5, 3.0 and 3.5 mM. Samples were collected at each ionic condition, and the final concentration of the samples was maintained at 200 μg/ml (DNA concentration) by supplementing with a two-fold concentrated hexanucleosomes equilibrated in 1X phase separation buffer. After incubation at room temperature for 30 min, the samples were transferred to the PEGylated and freshly passivated microscopy plates for microscopy imaging.

### Fluorescence microscopy

Microscopy images of nucleosome array condensates were captured with an inverted IX83 Olympus microscope with a 16-channel LED light source, equipped with a temperature-controlled chamber and a sCMOS camera (Hamamtsu Orca Flash4) using an Olympus LUC PLAN FLN 40x LWD/0.60 objective. The microscope was operated by the native Olympus cellSens Dimension software. Images from each well were acquired with identical microscopy settings and then analyzed, and the mean pixel intensity and standard deviation were calculated in IMARIS image analysis software, from which the grayscale intensities of the coefficient of variation values were determined.

### Cryo-electron microscopy

Hexanucleosome array samples were prepared as described above and then concentrated to approximately 200 μg/ml (DNA concentration), followed by overnight dialysis to achieve the desired final concentration of NaCl or MgCl_2_ as specified in the text. Quantifoil 1.2/1.3 grids were glow-discharged (25 mA, 30 s, 10^−1 ^Pa), 3 μl of each sample was deposited on these grids and flash frozen in liquid ethane using an automated plunger (Vitrobot, FEI) with controlled blotting time (1 s), blotting force (1), wait time (0), humidity (100%) and temperature (4 °C). Images were captured using either a TecnaiSphera F20 (FEI) microscope operating at 200 kV and fitted with a 4k × 4k Ceta (FEI) camera or a Tecnai Sphera G20 (FEI) microscope operating at 120 kV with a GATAN Ultrascan 1000 camera. Images were acquired under low-dose conditions (<20 e/A^2^), with a nominal underfocus ranging from 2 to 2.7 μm to enhance particle contrast. The pixel sizes of recorded images were 0.414 nm and 0.702 nm for the F20 and G20 respectively, and images were processed in Adobe Photoshop.

### FRAP analysis in mES and U2OS cells

#### Experiments in mES cells

mES cells in which endogenous H1.4 was replaced with photoactivatable GFP-tagged versions of either the WT, RS, or RSΔC H1.4 variants are described in ref. ^49^. For microscopy, cells were seeded in MatTek glass-bottom Petri dishes, plated with 0.1% gelatin in PBS. mES cells were transfected with an mTag-BFP2-H3.3-N-14 plasmid (Addgene #55302) encoding the histone H3.3-BFP fusion protein using Lipofectamine 2000, following the manufacturer’s guidelines, 24 h prior to imaging. H3.3-BFP fluorescence was used to target the nuclei of cells growing in clumps. FRAP experiments were conducted using an Andor Revolution Xdi spinning-disk confocal microscope system, outfitted with a FRAP-PA module and a live-cell imaging chamber. Photoactivation of H1.4-PAGFP was initiated at 405 nm within an 8 μm diameter region (35 pixels). After a 30-s interval, photobleaching of a smaller internal region (3.5 μm diameter) was performed at 488 nm, and recovery was monitored for the specified duration. Image analysis was carried out using the CellTool software^71^.

#### Experiments in U2OS cells

The pcDNA5/FRT vector (ThermoFisher) was linearized with MluI and HindIII to replace its promoter with a block containing the tetracycline repressor gene, followed by Tet-regulated CMV from pEBTetBlast-RNaseH1 (amplified with the following primers: 5’-cgatgtacgggccagatatataatacgactcactataggg and 5’- tggatccgagctcggtaccatctctatcactgataggg and inserted into pcDNA5FRT by Gibson assembly) to produce the T1 vector. For H1.4 tagging, we used a plasmid derivative of pEGFP-C1 (Clontech) in which a linker comprising 7 tandem GGGSGGG repeats was cloned by Gibson assembly to produce the vector C1L. The WT H1.4 coding sequence was amplified using primers 5’- GGTTCTGGTGGTGGTTCGATGTCCGAGACTGCGCCT and 5’- GGTACCGTCGACTGCAGCTACTTTTTCTTGGCTGCC and cloned into EcoRI-linearized C1L by Gibson assembly. Site-directed mutagenesis was carried out with overlapping primers, containing the RS (c.430dupG) mutation at the center: 5’-CAAGAAGGCGACGGGGGGCGGCCACCCCCAAGAAG and 5’-CTTCTTGGGGGTGGCCGCCCCCCGTCGCCTTCTTG. The WT and RS variant sequences (together with GFP and the linker, N-terminal to the histone) were recloned into the NotI-linearized T1 vector by Gibson assembly using primers 5’-AGATATCCAGCACAGTGGCATGGTGAGCAAGGGCGAGG and 5’-GGCCCTCTAGACTCGAGCCTACTTTTTCTTGGCTGCCGCC (5-CTACTTTTTCTTGGCTGCCGCCCTATTTGGGGTCTTCTTGGCGCTC for the RSΔC variant). The resulting pcDNA5FRT-GFP-H1.4 constructs (encoding the WT, RS, or RSΔC variants of H1.4) were integrated into U2OS-FlpIn cell line (Thermo Fisher) according to the manufacturer’s recommendations and GFP-positive cells were sorted three days later using a BD FACSDiscover™ S8 Cell Sorter. For microscopy, cells were seeded in MatTek glass-bottom Petri dishes. FRAP experiments were conducted using an Andor Revolution Xdi spinning-disk confocal microscope system, outfitted with a FRAP-PA module and a live-cell imaging chamber. Photobleaching of a 3.5-μm-diameter region was performed at 488 nm, and recovery was monitored over 120 s at 200 ms intervals. Image analysis was carried out using the CellTool software^71^.

### FRET experiments

#### Preparation of 207 bp 601 DNA fragments and mononucleosomes

207 bp DNA containing the 601 positioning sequence for mononucleosome reconstitution was achieved by PCR using Cy3- and Cy5-labeled primers (GenScript). The forward and reverse primer sequences were ATCGGACCC/iCy5N/ATACGCGGCC and AGTAG/iCy3N/ATTAATTAATATGAATTCGGATCCACATGCAC, where iCy3N and iCy5N indicate the positions of the Cy3 and Cy5 fluorophores, respectively. After PCR, fluorescently labeled 207 bp DNA fragments were purified using PCR purification kit (Qiagen). Nucleosomes were reconstituted using 2.5 μg of H3-H4 tetramer, 2.6 μg H2A-H2B dimer and 5 μg labeled 207 bp 601 DNA in a total volume of 200 μL reconstitution buffer (10 mM Tris, pH 8.0, 1 mM EDTA, 5 mM DTT and 2 M NaCl) by stepwise dialysis. The sample was dialyzed against TE buffer (10 mM Tris, 1 mM EDTA, pH 8.0) containing decreasing concentrations of NaCl at 1.2, 1, 0.8, and 0.6 M, each for 2 h at 4 °C, followed by dialysis against TE overnight at 4 °C. Reconstituted nucleosomes were purified over 10 ml 7–20% sucrose gradients and pure fractions were concentrated as described previously (Hao et al. 2020). Purified nucleosomes were analyzed on 0.7% agarose gels.

#### FRET analysis

Nucleosomes containing fluorophore-labeled 207 bp 601 DNA (final concentration 4.4 nM) in H1 binding buffer (10 mM Tris–HCl pH 8.0, 50 mM NaCl, 300 ng/ul BSA) were mixed with either the WT, RS mutant or RSΔC mutant form of H1.4 to ensure saturated H1.4 binding by nucleosomes. Emission spectra were recorded with excitation at 515 nm (Cy3 donor) and 610 nm (Cy5 acceptor) wavelengths with 5-nm slit widths in both excitation and emission channels on a Horiba Jobin Yvon FluoroMax-4 spectrofluorometer. The spectrum of the H1 binding buffer was recorded as a control and used for background subtraction. The FRET efficiency was determined by the (ratio)_A_ method, as described previously (Hao et al. 2020), using Eqs. (1) and (2). For this work, ϵ^D^(515) = 92 058 cm^−1^ M^−1^ (Cy3), ϵ^A^(515) = 6078 cm^−1^ M^−1^ (Cy5), ϵ^A^(610) = 161,103 cm^−1 ^M^−1^ (Cy5), *λ*’ = 515 nm, *λ*” = 610 nm and *d*^+^ = 1.

1 $${\left({{\mathrm{ratio}}}\right)}_{A}=\frac{E{\varepsilon }^{{{{\rm{D}}}}}\left({\lambda }^{{\prime} }\right){d}^{+}+{\varepsilon }^{{{{\rm{A}}}}}\left({\lambda }^{{\prime} }\right)}{{\varepsilon }^{{{{\rm{A}}}}}\left({\lambda }^{{\prime} {\prime} }\right)}$$

2 $$E=\frac{{{{{\rm{\varepsilon }}}}}^{{{{\rm{A}}}}}\left({{{{\rm{\lambda }}}}}^{{\prime} {\prime} }\right){({ratio})}_{A}-{{{{\rm{\varepsilon }}}}}^{{{{\rm{A}}}}}\left({{{{\rm{\lambda }}}}}^{{\prime} }\right)}{{{{{\rm{\varepsilon }}}}}^{{{{\rm{D}}}}}\left({{{{\rm{\lambda }}}}}^{{\prime} }\right){d}^{+}}$$

### Molecular dynamics simulations

Atomistic models of the nucleosome bound to WT human H1.4 and to the Rahman Syndrome (RS) mutant were generated using the X-ray structure of the nucleosome containing the GH1 domain of *Xenopus laevis* H1.0b (PDB ID 5NL0^9^). In this structure, the linker histone isoform was replaced by the GH1 domain of the human H1.4 coordinates obtained from a more recent cryo-EM study (PDB ID 7K5Y^10^). H1.4 CTD sequences of the WT and the c.430dup Rahman syndrome (RS) constructs were obtained from the Universal Protein (UniProt) database and a whole-exome study on RS patients^72^, respectively. The DNA sequence of the RS H1.4 CTD was translated into the amino acid sequence using the Biopython package. The atomic coordinates of each CTD were generated and joined to the GH1 domain of the H1.4 structure via PyMOL. Each nucleosome was solvated in a triclinic water box system containing 161.5 mM NaCl and 5 mM Mg^2+^ ions to mimic physiological intracellular conditions. Both systems were energy-minimizated using a combination of conjugate gradient and steepest descent algorithms, followed by a two-step NVT equilibration, first at 100 K, then at 310 K, followed by the production run in the NPT ensemble at 310 K and 1 atm. 100 K and 310 K simulations were integrated using time steps of 1 fs and 2 fs, respectively. The aqueous environment and the biological material were represented via the Optimal Point Charge water model^73^ and the CHARMM36m force field^74^, respectively. NBFIX correction^75^ was used for the interactions involving ions. To mimic the experimental nucleosome reconstitution and subsequent H1 integration, solvated RS H1.4 was equilibrated alone for 100 ns before integration into the nucleosome. Solvated WT H1.4 maintains its elongated CTD configuration in the absence of the nucleosome. Production run simulations were collected for 500 ns for both the WT and the RS systems in the NPT ensemble. The first 250 ns of each trajectory were considered equilibration, and the second 250 ns were considered for subsequent analyses. MD trajectories were collected using the Groningen Machine for Chemical Simulation (GROMACS) software, version 2018^76^. Coordinates were recorded every 100 ps. Preparation of the molecular setups, numerical analyses and visualizations were performed using Gromacs tools, PyMOL, and Visual Molecular Dynamics, as well as in-house Python and Bash scripts. Interfacial contacts were defined geometrically by a 5 Å distance cutoff between non-hydrogen atoms. Pairwise end-to-end distances are computed between the mass centers of the non-hydrogen atoms comprising on each side the three linker DNA base pairs 88th through 90th. DNA affinities of linker histones were computed via a modified version of the PDA-Pred method^77^ by computing and averaging over the affinities in every recorded frame within the 250–500 ns intervals of the NPT ensemble trajectories. DNA-pulling simulations were initiated using the final chromatosome conformations by removing the nucleosome core particles and retaining only the H1.4 proteins and the two linker DNA fragments, each of length 25 base pairs. The solvent environments are re-built, energy minimized and equilibrated using the same protocols as in the earlier simulations but this time using a cubic box of side length 200 Å. DNA fragments are pulled away from each other using Adiabatic Bias Molecular Dynamics (ABMD)^78^ as implemented in PLUMED 2.9.0 library^79^ patched with GROMACS. For both WT and RS simulations, the reaction coordinate is chosen as the distance between the centers of mass of the linker DNA fragments. The fragments are pulled away from each other for 6 ns using a force constant of 10 kJ mol^–1^ Å^–2^ toward a target value of 100 Å.

### Statistics and reproducibility

All experiments were repeated at least twice with independently prepared samples. Sample sizes and the number and type of replicates are specified in the figure legends. Statistical analyses were performed in GraphPad Prism using two-sided Student’s *t* tests, two-sided Fisher’s exact tests, or chi-square tests, as indicated in the relevant figure legends.

### Reporting summary

Further information on research design is available in the Nature Portfolio Reporting Summary linked to this article.

## Supplementary information

Supplementary Information

Transparent Peer Review file

Reporting summary

Description of Additional Supplementary Files

Supplementary Movie 1

Supplementary Movie 2

Supplementary Movie 3

Supplementary Movie 4

## Source data

Source data

## Data Availability

The data supporting the findings of this study are available within the article and its supplementary material, including additional references^80–88^. Supplementary Figs. 1–13, Supplementary Tables 1, 2 and Supplementary Movies 1–4 are in the supplementary information. Uncropped gel images and the values that were used to create all graphs can be found in the accompanying “Source data” file. Source data are provided with this paper.
