# Supplementary material for: A Rahman Syndrome mutation in histone H1.4 disrupts chromatin compaction and phase separation
Source: Nat Commun. 2026 May 22;17:6727. doi: 10.1038/s41467-026-73046-8 (PMC13385758; doi:10.1038/s41467-026-73046-8)

Supplementary Information

A Rahman Syndrome mutation in histone H1.4 disrupts chromatin compaction and phase separation

Ramachandran Boopathi, Isabel Garcia-Saez, Serhan Turunç, Imtiaz Nisar Lone, Ashok Kumar, Abed Alkarem Abu Alhaija, Jeff Hayes, Jan Bednar, Kasim Diril, Dimitar Iliev, Anastas Gospodinov, Aline Le Roy, Dimitrios Skoufias, Dimitar Angelov, Ali Hamiche[*], Seyit Kale[*], Stefan Dimitrov[*] and Carlo Petosa[*]

Supplementary Table 1: Summary of SAXS data collection and analysis parameters

**Data collection parameters**	
Source and instrument	ESRF beamline BM-29
Wavelength (Å)	0.9918
Sample-to-detector distance	2.879 m
q-measurement range (nm^{-1})	0.052 - 5.0
Detector	Pilatus 1M
Basis for normalization to constant counts	Normalization based on beam intensity and exposure time using the BM29 data processing pipeline
Method for monitoring radiation damage	Radiation damage was monitored by comparison of successive frames, with aggregation-affected frames excluded
No. of exposures, exposure time	10 frames of 1 s duration per sample
Sample temperature	20°C
Sample configuration	Samples (40-50 μL) were loaded into a vacuum-mounted quartz capillary (1.8 mm diameter) and measured under continuous flow during data acquisition
**Software employed for data reduction and analysis**	
Initial processing and azimuthal integration	EDNA
Primary data reduction and buffer subtraction	PRIMUS
Determination of R_g and D_{max}	PRIMUS
Pair-distance distribution function $P(r)$	GNOM
Bead modelling	DAMMIF/DAMAVER/DAMMIN
Molecular graphics	PyMOL

Supplementary Table 2: Summary of parameters derived from SAXS analysis of H1.4-bound hexanucleosomes.

Ionic conditions	H1.4	D_{max} (nm)	R_g (nm)	χ^2 of *ab initio* refined model
50 mM NaCl	WT	44.6	13.2	0.844
	RS	59.5	16.5	0.920
	RSΔC	50.4	15.2	0.827
90 mM NaCl	WT	38.1	12.0	0.765
	RS	43.8	13.4	0.516
	RSΔC	47.1	13.8	1.150
0.35 mM $MgCl_2$	WT	45.2	13.1	0.894
	RS	57.7	16.9	0.910
	RSΔC	55.3	16.8	1.251
0.50 mM $MgCl_2$	WT	39.9	13.0	1.645
	RS	53.7	16.1	1.388
	RSΔC	53.1	15.9	0.819
0.60 mM $MgCl_2$	WT	40.1	12.2	2.292
	RS	52.0	15.6	0.933
	RSΔC	52.9	16.1	0.935

110 . 120 . 130 . 140 . 150 . 160 . 170 . 180 . 190 . new stop 200 . 210 . 219

Nucleotide change	Protein change	Ref.	CTD Sequence	Net charge
WT	WT	f	KAASGEAKPKAKKAGAAKAKKPAGAAKKPKKATGAATPKKSAKKTPKKAKKPAAAAGAKKAKSPKKAKAAKPKKAPKSPAKAKAVKPKAAKPKTAKPKAAKPKKAAAKKK	+43
c.360_361insA	p.Ala123Glyfs*73	f	KAASGEAKPKAKK-GRRGQGQEASRSGEEAQEGDGGGHPQEERQEDPKEGEEAGCSCWSQKSEKPEKGESSQAKKGAQEPSEGQSS	-3
c.368dupC	p.Gly124Argfs*72	k	KAASGEAKPKAKKA-RRGQGQEASRSGEEAQEGDGGGHPQEERQEDPKEGEEAGCSCWSQKSEKPEKGESSQAKKGAQEPSEGQSS	-3
c.392dupC	p.Ala132Serfs*64	j	KAASGEAKPKAKKAGAAKAKKP-SRSGEEAQEGDGGGHPQEERQEDPKEGEEAGCSCWSQKSEKPEKGESSQAKKGAQEPSEGQSS	-1
c.392_395dup	p.Gly133Serfs*64	h	KAASGEAKPKAKKAGAAKAKKPASRSGEEAQEGDGGGHPQEERQEDPKEGEEAGCSCWSQKSEKPEKGESSQAKKGAQEPSEGQSS	-1
c.406_407insT	p.Lys136Ilefs*60	f	KAASGEAKPKAKKAGAAKAKKPAGAA-IEAQEGDGGGHPQEERQEDPKEGEEAGCSCWSQKSEKPEKGESSQAKKGAQEPSEGQSS	-1
c.407dupA	p.Lys137Glufs*59	f	KAASGEAKPKAKKAGAAKAKKPAGAAK-EAQEGDGGGHPQEERQEDPKEGEEAGCSCWSQKSEKPEKGESSQAKKGAQEPSEGQSS	0
c.408dupG	p.Lys137Glufs*59	e	KAASGEAKPKAKKAGAAKAKKPAGAAK-EAQEGDGGGHPQEERQEDPKEGEEAGCSCWSQKSEKPEKGESSQAKKGAQEPSEGQSS	0
c.414dupC	p.Lys139Glnfs*57	e	KAASGEAKPKAKKAGAAKAKKPAGAAKKP-QEGDGGGHPQEERQEDPKEGEEAGCSCWSQKSEKPEKGESSQAKKGAQEPSEGQSS	+2
c.416dupA	p.Lys140Glufs*56	f	KAASGEAKPKAKKAGAAKAKKPAGAAKKPK-EGDGGGHPQEERQEDPKEGEEAGCSCWSQKSEKPEKGESSQAKKGAQEPSEGQSS	+3
c.416_419dupAGAA	p.Ala141Glufs*56	m	KAASGEAKPKAKKAGAAKAKKPAGAAKKPKKEGDGGGHPQEERQEDPKEGEEAGCSCWSQKSEKPEKGESSQAKKGAQEPSEGQSS	+4
c.425delinsAG	p.Thr142Lysfs*54	e	KAASGEAKPKAKKAGAAKAKKPAGAAKKPKKA-KGGGHPQEERQEDPKEGEEAGCSCWSQKSEKPEKGESSQAKKGAQEPSEGQSS	+7
c425_431delinsAGGGGGTT	p.Thr142Lysfs*54	e, f	KAASGEAKPKAKKAGAAKAKKPAGAAKKPKKA-KGVGHPQEERQEDPKEGEEAGCSCWSQKSEKPEKGESSQAKKGAQEPSEGQSS	+7
**c.430dupG**	p.Ala144Glyfs*52	b,e,f,j	KAASGEAKPKAKKAGAAKAKKPAGAAKKPKKATG-GGHPQEERQEDPKEGEEAGCSCWSQKSEKPEKGESSQAKKGAQEPSEGQSS	+6
c.431dupC	p.Ala145Glyfs*51	f,j	KAASGEAKPKAKKAGAAKAKKPAGAAKKPKKATGA-GHPQEERQEDPKEGEEAGCSCWSQKSEKPEKGESSQAKKGAQEPSEGQSS	+6
c.433dupG	p.Ala145Glyfs*51	d,f	KAASGEAKPKAKKAGAAKAKKPAGAAKKPKKATGA-GHPQEERQEDPKEGEEAGCSCWSQKSEKPEKGESSQAKKGAQEPSEGQSS	+6
c.435dupC	p.Thr146Hisfs*50	c,f,j	KAASGEAKPKAKKAGAAKAKKPAGAAKKPKKATGAA-HPQEERQEDPKEGEEAGCSCWSQKSEKPEKGESSQAKKGAQEPSEGQSS	+6
c.436_458del23	p.Thr146Asp*42	b,f,g	KAASGEAKPKAKKAGAAKAKKPAGAAKKPKKATGAA---------DPKEGEEAGCSCWSQKSEKPEKGESSQAKKGAQEPSEGQSS	+7.9
c.437_438del	p.Pro147Glnfs*48	f	KAASGEAKPKAKKAGAAKAKKPAGAAKKPKKATGAAT--QEERQEDPKEGEEAGCSCWSQKSEKPEKGESSQAKKGAQEPSEGQSS	+5.9
c.441dupC	p.Lys148Glnfs*48	a,b,e,f,l	KAASGEAKPKAKKAGAAKAKKPAGAAKKPKKATGAATP-QEERQEDPKEGEEAGCSCWSQKSEKPEKGESSQAKKGAQEPSEGQSS	+5.9
c.444_466del23	p.Lys149Glufs*39	f	KAASGEAKPKAKKAGAAKAKKPAGAAKKPKKATGAATPK---------EGEEAGCSCWSQKSEKPEKGESSQAKKGAQEPSEGQSS	+8.9
c.447dupG	p.Ser150Glufs*46	e	KAASGEAKPKAKKAGAAKAKKPAGAAKKPKKATGAATPKK-ERQEDPKEGEEAGCSCWSQKSEKPEKGESSQAKKGAQEPSEGQSS	+8.9
c.454_455insT	p.Lys152Ilefs*44	f	KAASGEAKPKAKKAGAAKAKKPAGAAKKPKKATGAATPKKSA-IEDPKEGEEAGCSCWSQKSEKPEKGESSQAKKGAQEPSEGQSS	+8.9
c.464dupC	p.Lys157Glufs*39	e	KAASGEAKPKAKKAGAAKAKKPAGAAKKPKKATGAATPKKSAKKTPK-EGEEAGCSCWSQKSEKPEKGESSQAKKGAQEPSEGQSS	+12.9
c.505_506insT	p.Lys169Ilefs*27	i	KAASGEAKPKAKKAGAAKAKKPAGAAKKPKKATGAATPKKSAKKTPKKAKKPAAAAGAK-ISEKPEKGESSQAKKGAQEPSEGQSS	+19

Supplementary Fig. 1. Sequence alignment of H1.4 variants identified in RS patients.
The c.430dupG variant used in this study is boxed. The predicted net charge at pH 7 of the CTD is indicated. All variants share a common C-terminal stretch of 38 residues enriched in acidic amino acids, except for c.505_506insT, which shares only 25 residues with this sequence. References are: *a* - Helsmoortel, 2015 [80]; *b* - Tatton-Brown, 2017 [1]; *c* - Duffney, 2018 [81]; *d* - Takenouchi, 2018 [82]; *e* - Flex, 2019 [2]; *f* - Burkardt, 2019 [3]; *g* - Pelle, 2021 [83]; *h* - Indugula, 2022 [72]; *i* - Tremblay, 2022 [31]; *j* - Zhao, 2022 [84]; *k* - Tanabe, 2023 [85]; *l* - Zhao, 2023 [86].

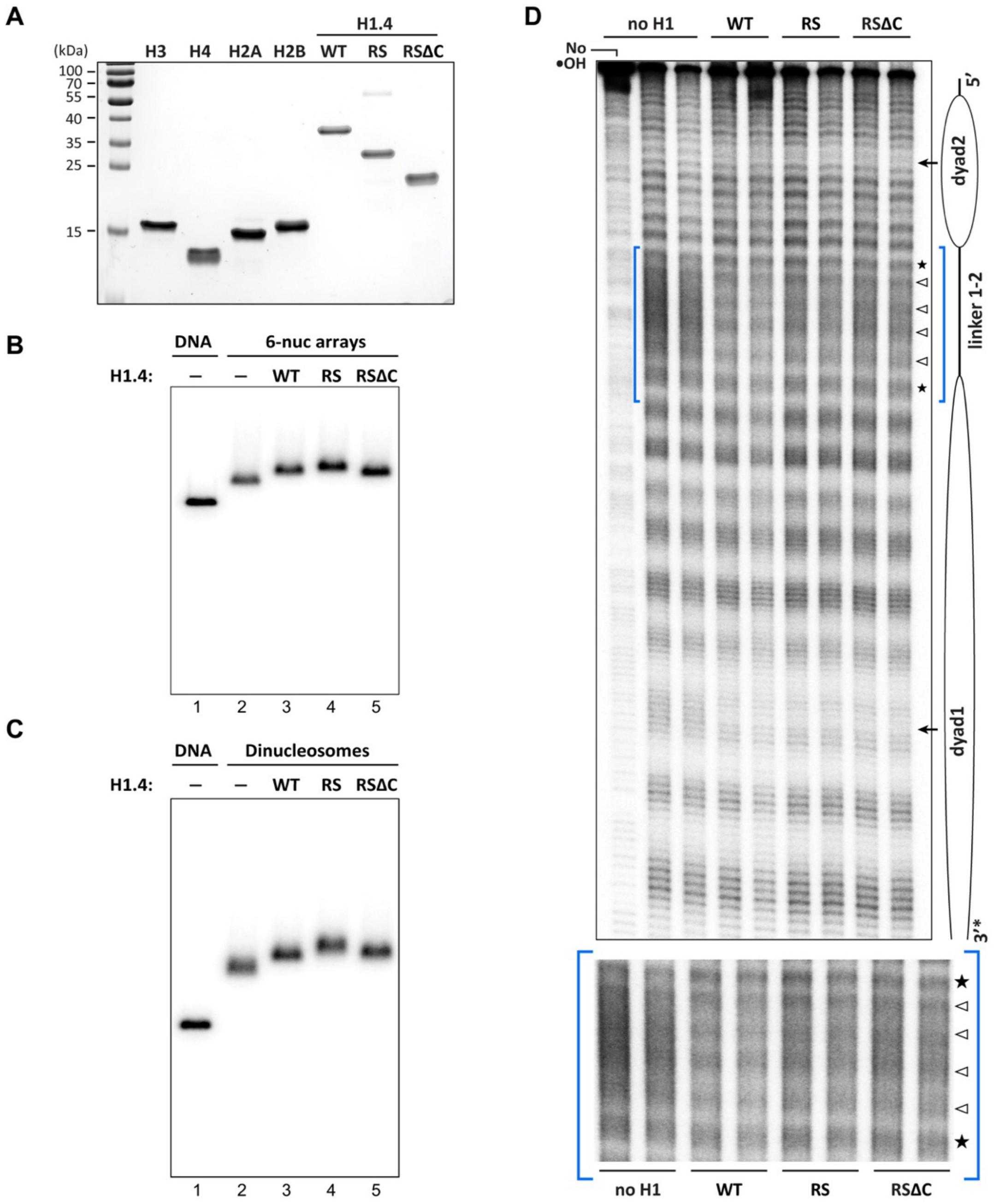

Supplementary Fig. 2. Analysis of nucleosome arrays and dinucleosomes.

(**A**) 18% SDS polyacrylamide gel electrophoresis (PAGE) showing the purity of recombinant histones.

(B) Autoradiogram of 6 × 197 bp hexanucleosome arrays analyzed by native agarose gel electrophoresis. Arrays were reconstituted in the absence (lane 2) or presence (lanes 3-5) of the WT, RS or RSΔC forms of H1.4. Naked DNA was included as a control (lane 1).

(**C**) Autoradiogram of 2 × 197 bp dinucleosomes analyzed by native agarose gel electrophoresis. Dinucleosomes were reconstituted in the absence (lane 2) or presence (lanes 3-5) of the WT, RS or RSΔC forms of H1.4. Naked DNA was included as a control (lane 1).

(**D**) Hydroxyl radical footprinting analysis showing that the RS mutation enhances DNA linker accessibility in dinucleosomes. Arrows denote regions of nucleotide base protection at the dyad in nucleosomes N1 and N2. Open arrowheads indicate cleavage products within the linker DNA. Asterisks indicate the 10 bp regions at both ends of the linker DNA adjacent to the nucleosome core region. No •OH, free DNA.

Experiments shown in (A)-(D) were each repeated independently three times with similar results.

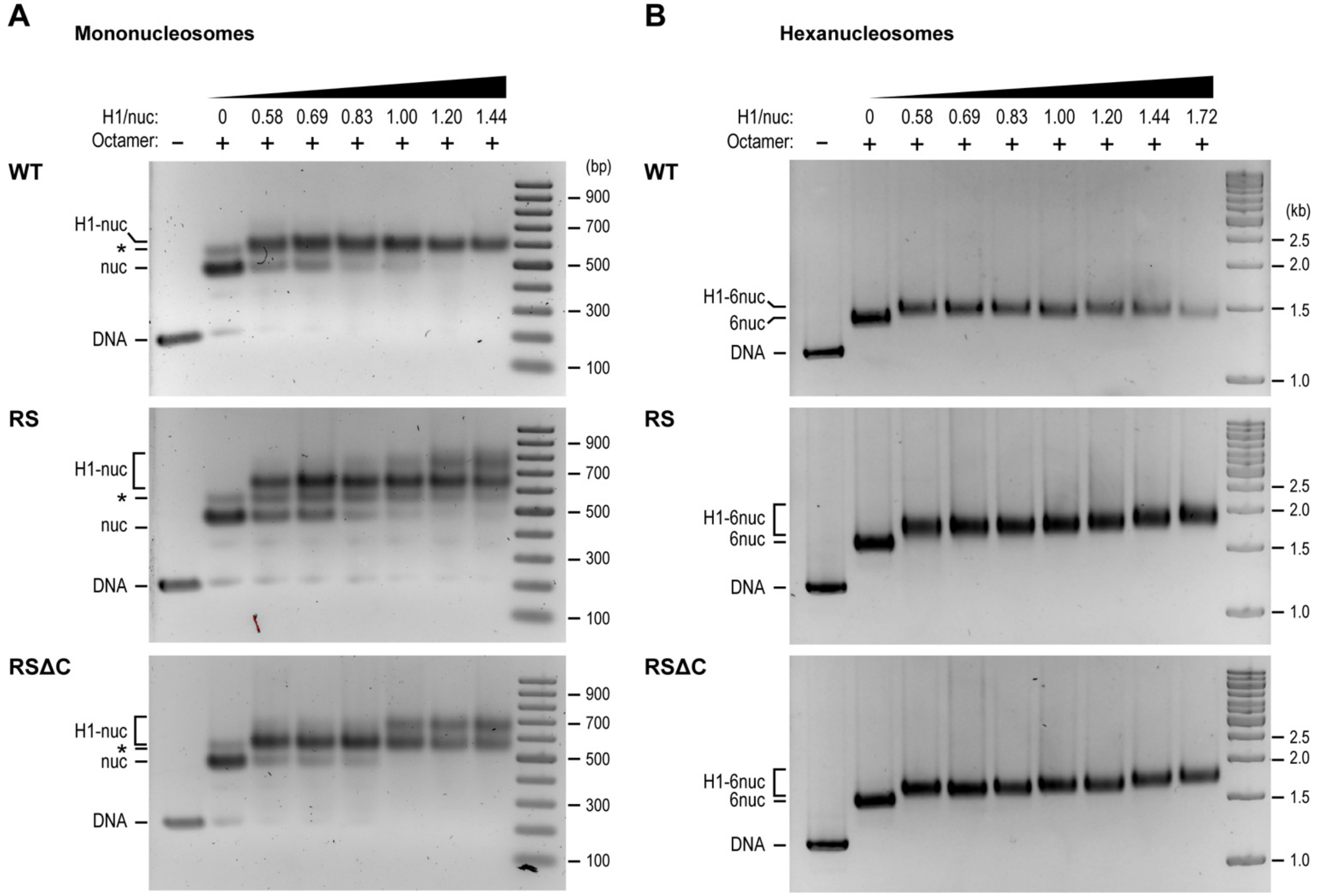

Supplementary Fig. 3. WT and mutant H1.4 variants are incorporated into nucleosomes with comparable efficiency.

(A) EMSAs of mononucleosomes (207 bp) reconstituted with increasing amounts of each H1.4 variant, as indicated by the H1:nucleosome stoichiometry (H1/nuc). The asterisk indicates a minor species present in the nucleosome preparation, likely corresponding to tetrasomes (DNA bound to an H3-H4 tetramer). At H1/nucleosome ratios above 1, the RS and RSΔC mutants display an additional band, consistent with binding to a secondary site on the nucleosome, whereas the WT sample shows partial precipitation that reduces overall band intensity.

(B) EMSAs of hexanucleosome arrays (6×187 bp) reconstituted with increasing amounts of each H1.4 variant. As with mononucleosomes, the WT array displays partial precipitation at H1/nucleosome ratios above 1, reducing band intensity, whereas the mutant arrays show decreased mobility, consistent with additional H1.4 molecules binding to secondary sites on the array.

Experiments shown in (A) and (B) were each repeated independently three times with similar results.

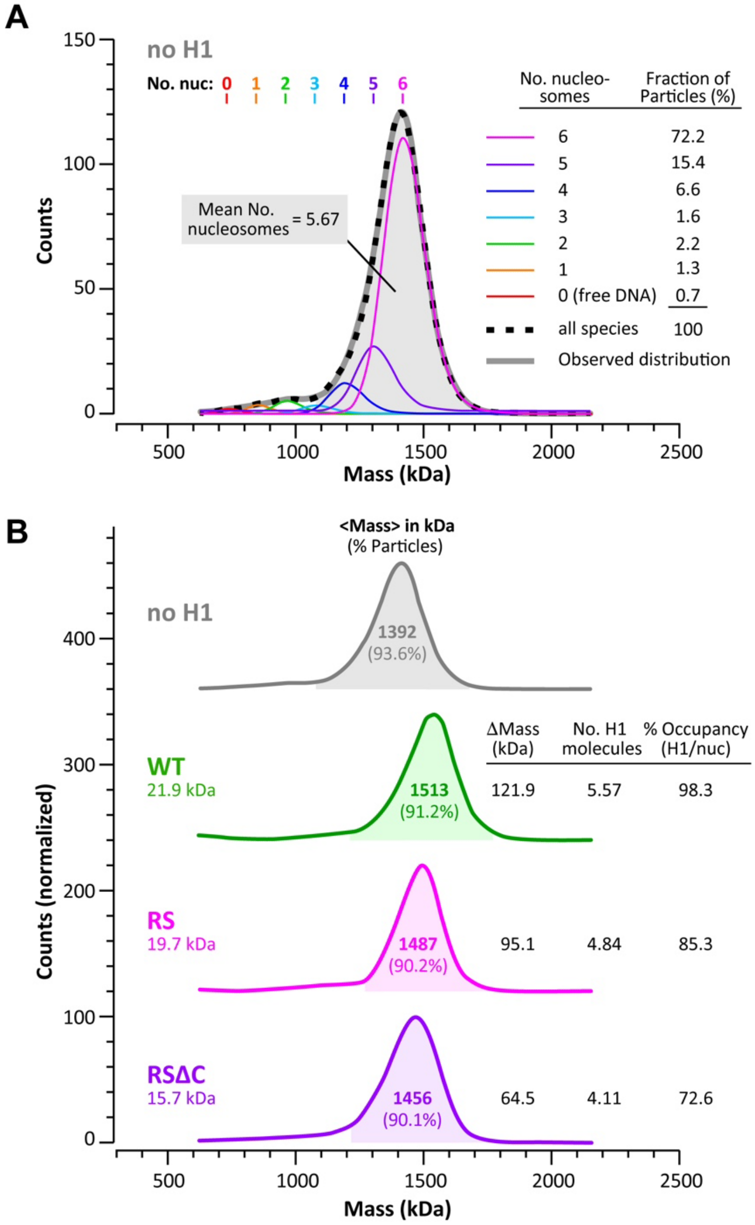

Supplementary Fig. 4. Mass photometry analysis of H1.4 occupancy in hexanucleosome arrays.

(A) Mass photometry of H1-free arrays. The observed mass distribution (solid grey line) was fitted as a population mixture model (dotted black line) comprising free DNA (red curve) and arrays containing one to six nucleosomes (orange, green, cyan, blue, purple and magenta curves, respectively). The component distributions were modeled as identically shaped exponentially modified Gaussian functions of variable amplitude, evenly spaced to reflect successive nucleosome additions. The primary peak of the observed distribution is shaded in grey. The mean nucleosome number per array, calculated from the fitted component distributions within the shaded area, is indicated. Distributions in panels A and B are those of Fig. 1C.

(B) Mass shift analysis of H1-bound arrays relative to the H1-free array. The primary peak of each distribution (shaded) is labeled with the mean particle mass and the fraction of total particles within the peak (in parentheses). The mean mass shift (ΔMass) relative to the H1-free array and the corresponding number of incorporated H1 molecules are indicated. Estimated H1 occupancies (H1-to-nucleosome ratios) were calculated using the mean nucleosome number determined in panel A. Although the RS and RSΔC arrays show slightly lower occupancies than WT, these modest differences cannot account for the pronounced biophysical and structural differences observed among the arrays. Moreover, because mass photometry was performed at low concentration (~15 nM, or 20-fold lower than in AUC, cryo-EM and phase separation experiments), the measured occupancies likely represent lower-bound estimates due to partial dissociation, which would preferentially affect arrays bound by the lower-affinity mutant variants.

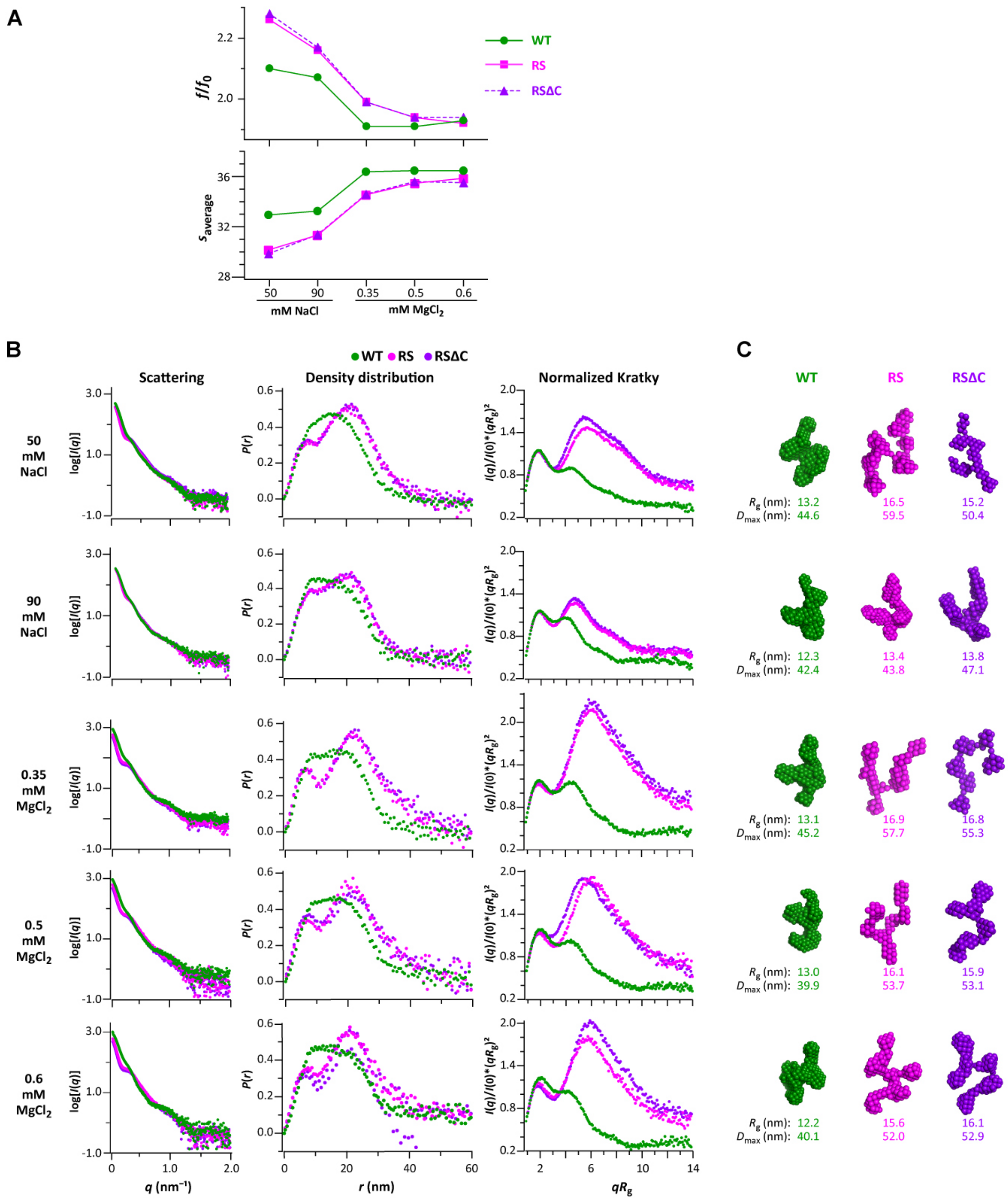

Supplementary Fig. 5. The RS mutation induces a more extended and flexible hexanucleosome.
Data for hexanucleosomes bound to the WT, RS mutant and RSΔC mutant forms of H1.4 are shown in green, magenta and purple, respectively.
(**A**) Average sedimentation coefficient (s_{ave}) and frictional ratio (f/f_0) for H1.4-bound hexanucleosomes at the indicated NaCl and $MgCl_2$ concentrations.
(B) Plots of background-corrected scattering data (left), density distribution curves (middle) and normalized Kratky plots (right) for H1.4-bound hexanucleosomes at the indicated NaCl and $MgCl_2$ concentrations.
(**C**) *Ab initio* (DAMMIF) models derived from the SAXS data. Radius of gyration (*Rg*) and maximal diameter (*Dmax*) values are indicated below each model.

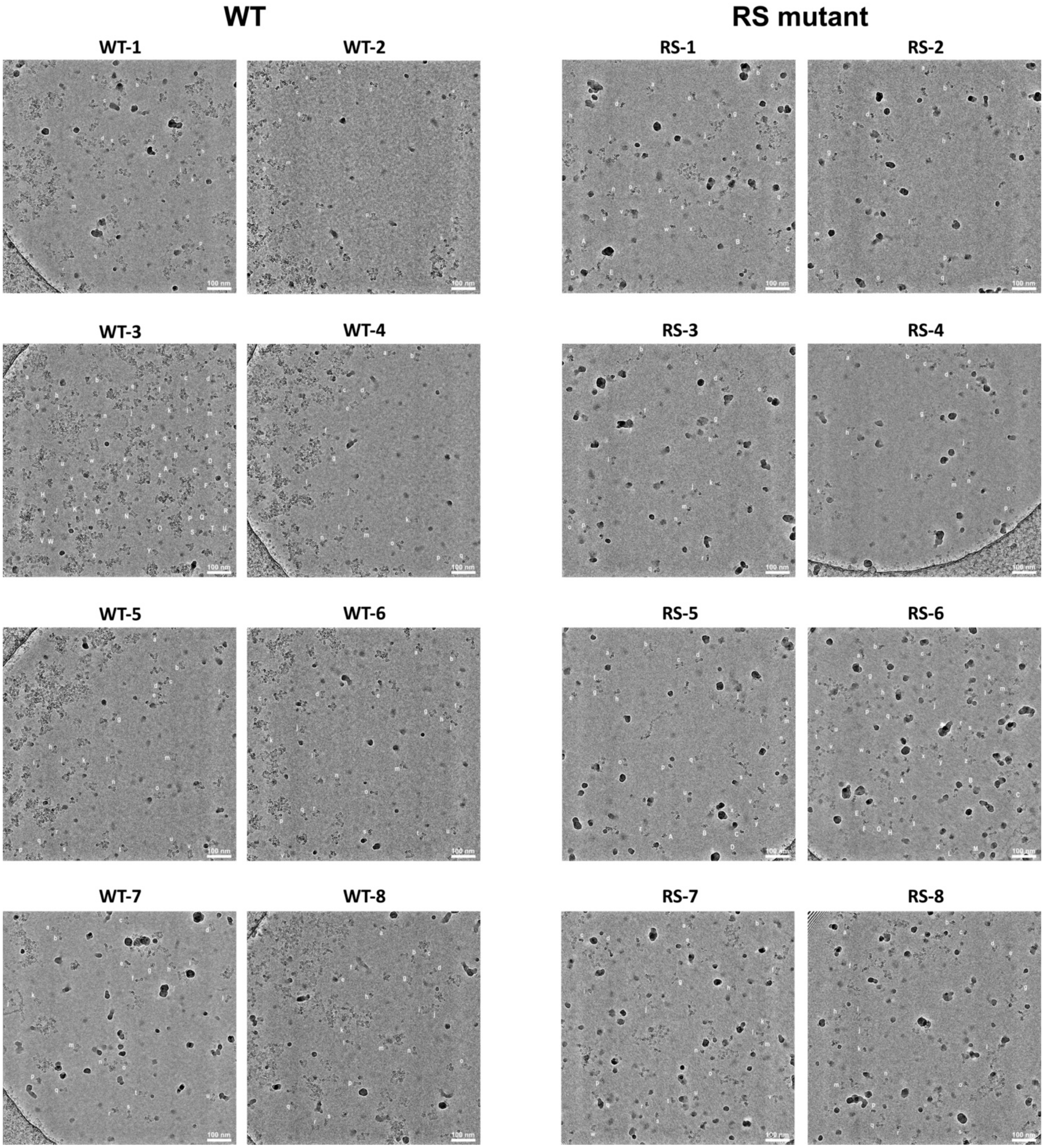

Supplementary Fig. 6. Full-field views of WT and RS mutant hexanucleosome arrays in 0.6 mM $MgCl_2$. Cryo-EM micrographs are shown for arrays bound to WT H1.4 (right panels) or the RS mutant (left panels). Isolated particles are labelled with lower- or upper-case letters, which index the corresponding images in Fig. 4A. The experiment was independently repeated twice with similar results.

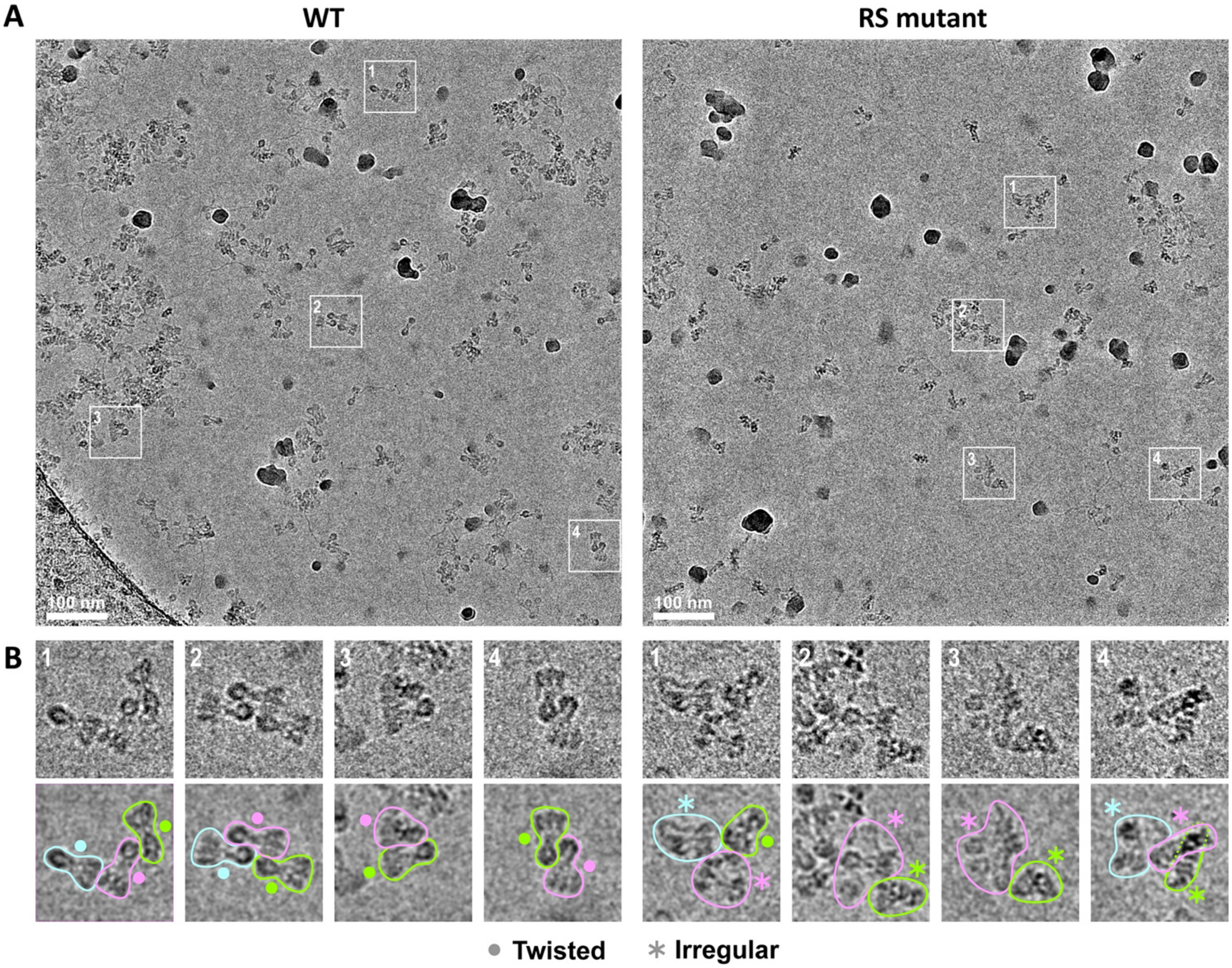

Supplementary Fig. 7. The RS mutation hampers proper folding of nucleosome arrays.

(A) Representative cryo-EM field views of hexanucleosomes bound to WT H1.4 or the RS mutant in 0.6 mM $MgCl_2$. The micrographs shown correspond to panels WT-1 and RS-1 in Supplementary Fig. 6.

(B) Closeup views of particle clusters from (A), shown before (top) and after (bottom row) applying a Gaussian blur to aid recognition of particle contours. Properly twisted conformations are marked with a circle, and irregular forms by an asterisk. Clusters of WT arrays often contain multiple twisted particles, whereas RS array clusters predominantly consist of irregular or poorly folded arrays. This trend is also evident across the images shown in Supplementary Fig. 6. The experiment was independently repeated twice with similar results.

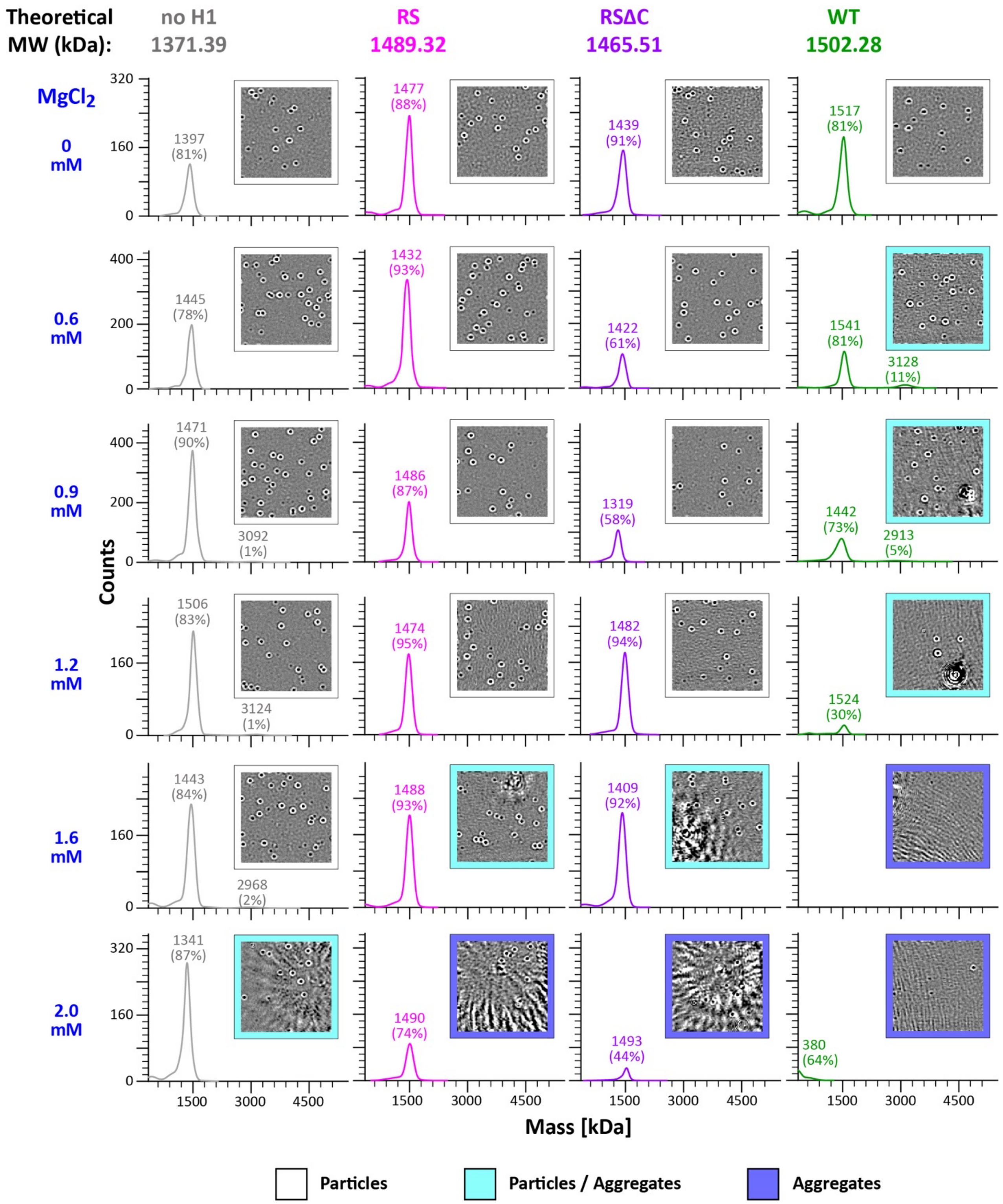

Supplementary Fig. 8. The RS mutation impairs chromatin phase separation. Molecular mass distribution and video frames obtained by mass photometry analysis of hexanucleosomes reconstituted in the presence or absence of the indicated form of histone H1.4. The sample behavior (particles, particles/aggregates and aggregates) observed in different $MgCl_2$ concentrations is highlighted in different colors as indicated.

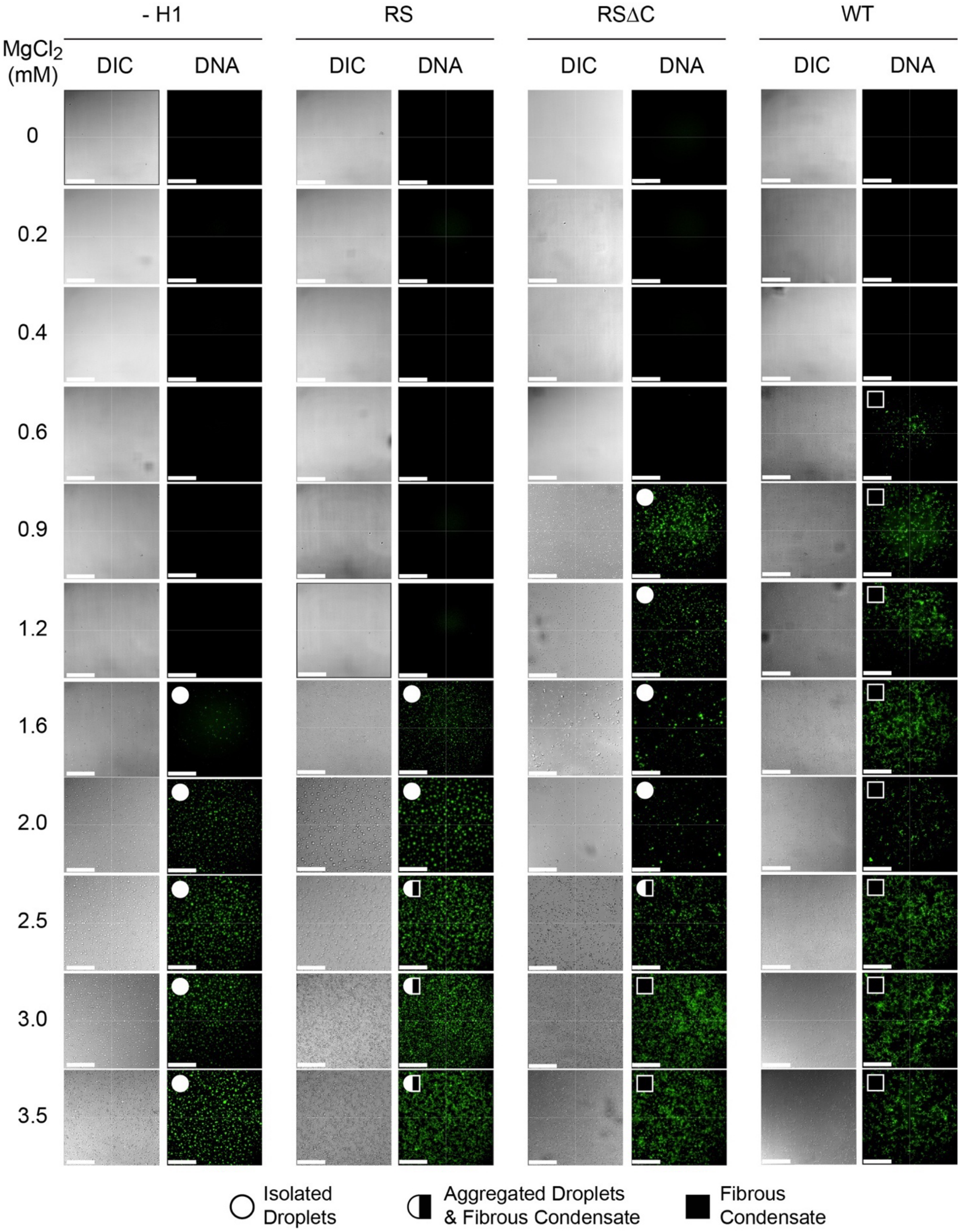

Supplementary Fig. 9. The RS mutation impairs chromatin phase separation. DIC and fluorescence microscopy images showing phase separation properties of hexanucleosomes at varying $MgCl_2$ concentrations. Circles and squares indicate the type of condensates observed: white circles, droplets; black squares, fibrous condensates. Scale bar, 30 μm.

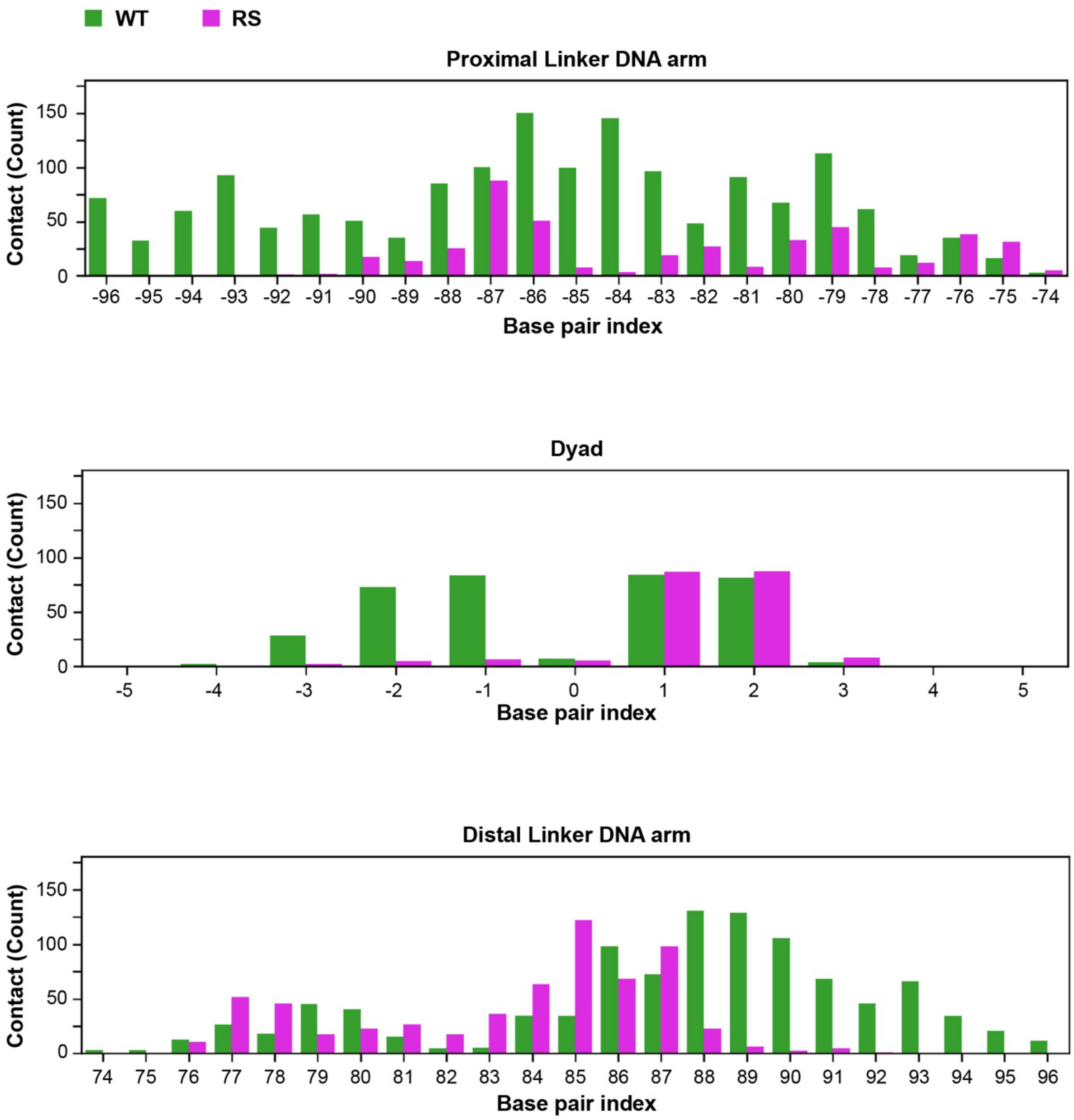

Supplementary Fig. 10. **The CTD of the RS variant exhibits reduced interactions with linker DNA terminal regions.** Number of contacts observed during the MD simulation between WT H1.4 (green) or the RS mutant (magenta) and the proximal DNA linker (upper panel), dyad DNA (middle panel) or distal linker (lower panel).

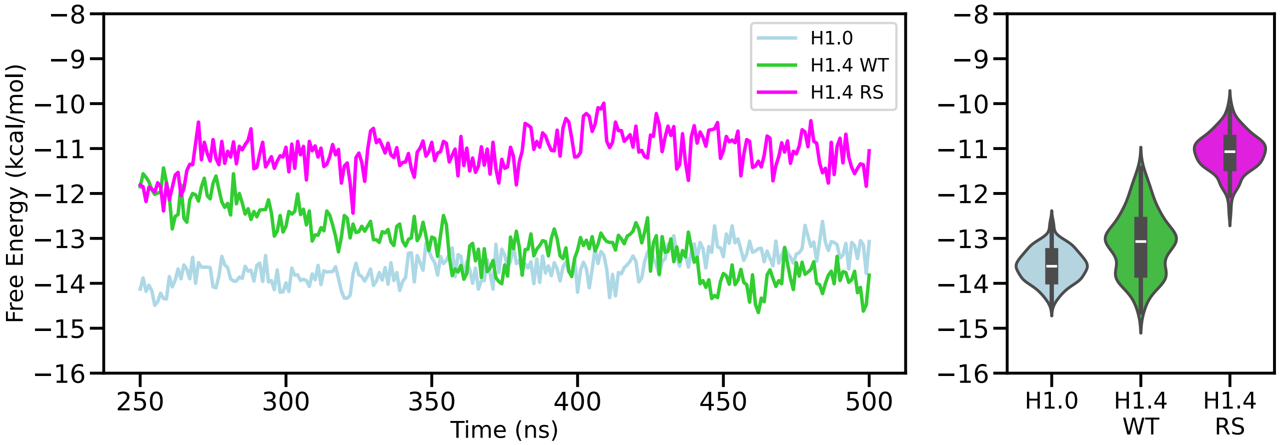

Supplementary Fig. 11. **Computed DNA affinities of the WT and RS mutant H1.4 linker histones.**
The left panel shows the predicted DNA binding free energy over time. The right panel shows the corresponding distributions as violin plots. To validate the predicted energies, the DNA-binding free energy of linker histone H1.0 was also computed using the same protocol, yielding a predicted value of –13.61 ± 0.35 kcal/mol, which is close to the average of the two experimental measurements performed by independent groups via native gel electrophoresis[87] and FRET[88] (–11.50 kcal/mol and –15.25 kcal/mol, respectively). Data were obtained from a single independent MD simulation (*n* = 1 trajectory) per setting (H1.0, H1.4 WT or H1.4Supp RS).

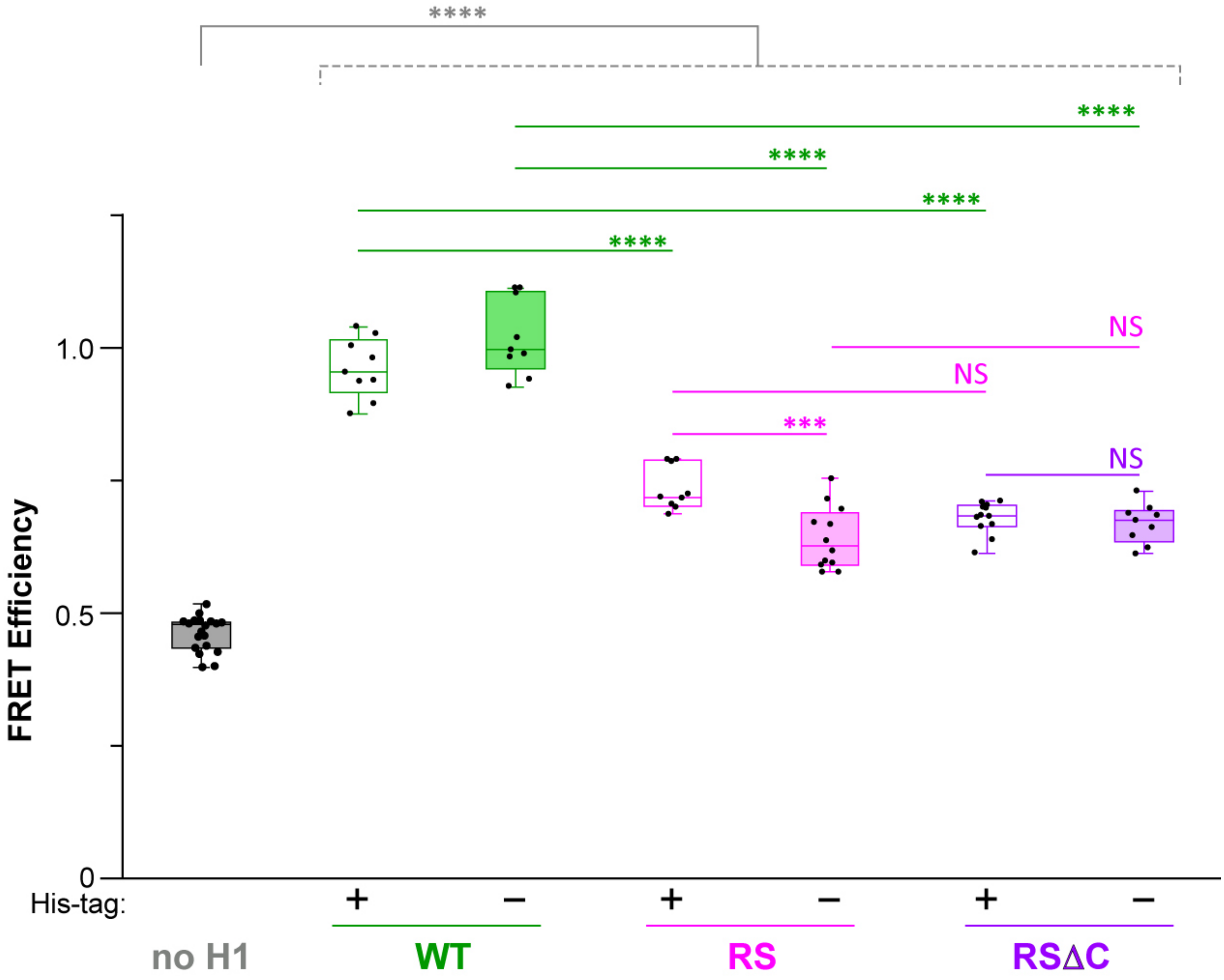

Supplementary Fig. 12. **The His-tag fused to WT and mutant forms of histone H1.4 does not affect FRET efficiency.** FRET efficiency was measured between the ends of nucleosomes bound to the indicated untagged or His-tagged H1.4.

Points represent individual FRET measurements (3 biological replicates, at least 3 technical replicates each), with box plots displaying the median, lower and upper quartiles, and minimum and maximum values. **** $p < 0.0001$; *** $p<0.005$; NS, not significant; p values were determined using two-sided Student's t-tests with Bonferroni correction in GraphPad Prism.

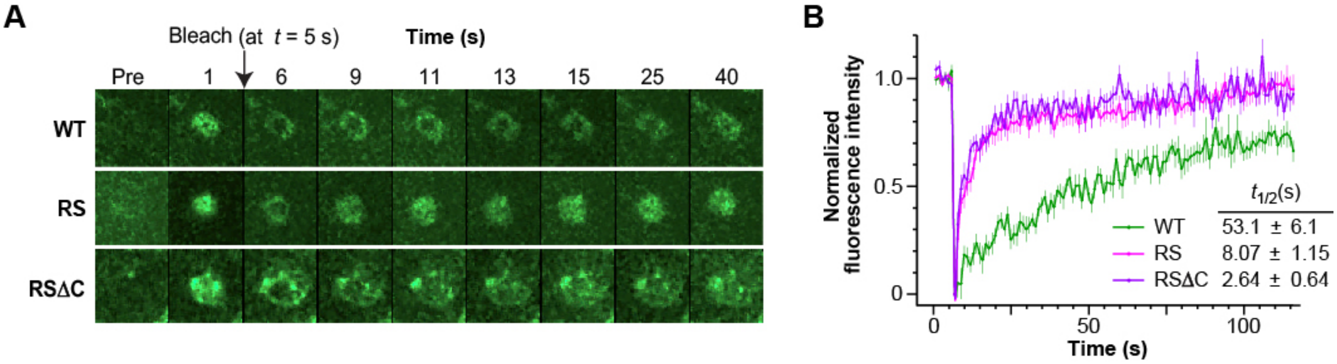

Supplementary Fig. 13. The RS mutation enhances H1.4 mobility *in vivo*.

(A) FRAP experiment in mES cells comparing the mobility of WT H1.4 with that of the RS and RSΔC mutants. The leftmost image in each series was recorded prior to photoactivation.

(B) Time course of fluorescence in bleached area, showing mean intensity and S.D. Data are from 2 (RSΔC) or 3 (WT, RS) independent experiments with at least 10 cells per condition per experiment.

SUPPLEMENTARY REFERENCES

80. Helsmoortel C, Vandeweyer G, Ordoukhanian P, Van Nieuwerburgh F, Van der Aa N, Kooy RF. Challenges and opportunities in the investigation of unexplained intellectual disability using family-based whole-exome sequencing. *Clin Genet* **88**, 140-148 (2015).
81. Duffney LJ*, et al.* Epigenetics and autism spectrum disorder: A report of an autism case with mutation in H1 linker histone HIST1H1E and literature review. *Am J Med Genet B Neuropsychiatr Genet* **177**, 426-433 (2018).
82. Takenouchi T, Uehara T, Kosaki K, Mizuno S. Growth pattern of Rahman syndrome. *Am J Med Genet A* **176**, 712-714 (2018).
83. Pelle A, Pezzoli L, Apuril E, Iascone M, Selicorni A. A novel HIST1HE pathogenic variant in a girl with macrocephaly and intellectual disability: a new case and review of literature. *Clin Dysmorphol* **30**, 39-43 (2021).
84. Zhao J*, et al.* Expanding the mutational spectrum of Rahman syndrome: A rare disorder with severe intellectual disability and particular facial features in two Chinese patients. *Mol Genet Genomic Med* **10**, e1825 (2022).
85. Tanabe Y*, et al.* HIST1H1E syndrome with deficiency in multiple pituitary hormones. *Clin Pediatr Endocrinol* **32**, 195-198 (2023).
86. Zhao W, Zhang Y, Lv T, He J, Zhu B. A case report of a novel HIST1H1E mutation and a review of the bibliography to evaluate the genotype-phenotype correlations. *Mol Genet Genomic Med* **11**, e2273 (2023).
87. Caterino TL, Fang H, Hayes JJ. Nucleosome linker DNA contacts and induces specific folding of the intrinsically disordered H1 carboxyl-terminal domain. *Mol Cell Biol* **31**, 2341-2348 (2011).
88. White AE, Hieb AR, Luger K. A quantitative investigation of linker histone interactions with nucleosomes and chromatin. *Sci Rep* **6**, 19122 (2016).

Uncropped Scans of Gels

Supplementary Fig. 2A

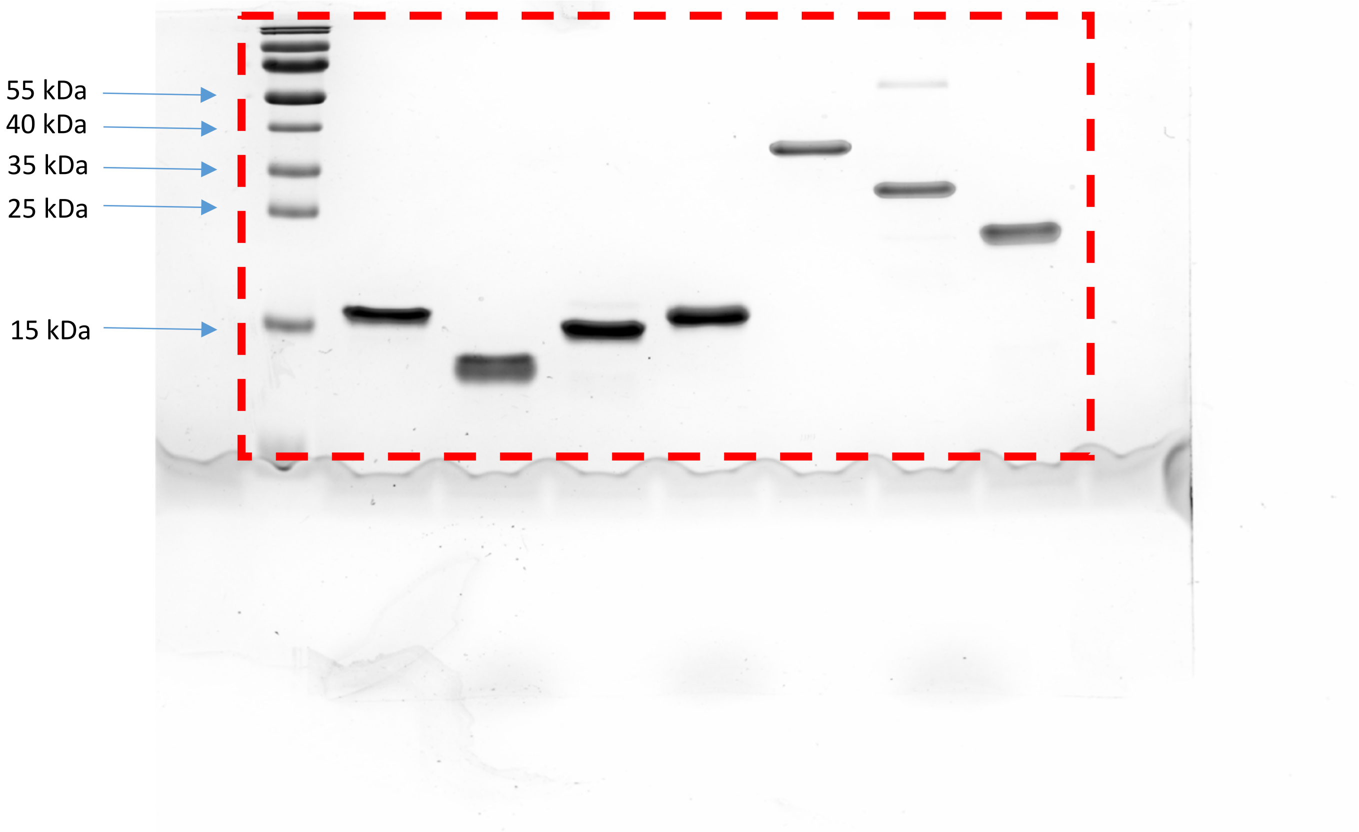

PageRuler™ Prestained Protein Ladder, 10 to 180 kDa (Thermo Fisher Scientific, catalog No. 26616)

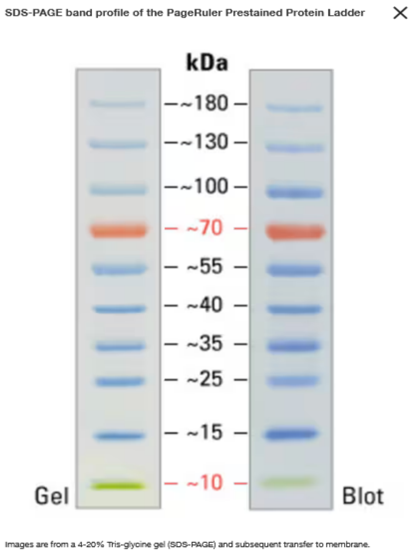

Supplementary Fig. 2B

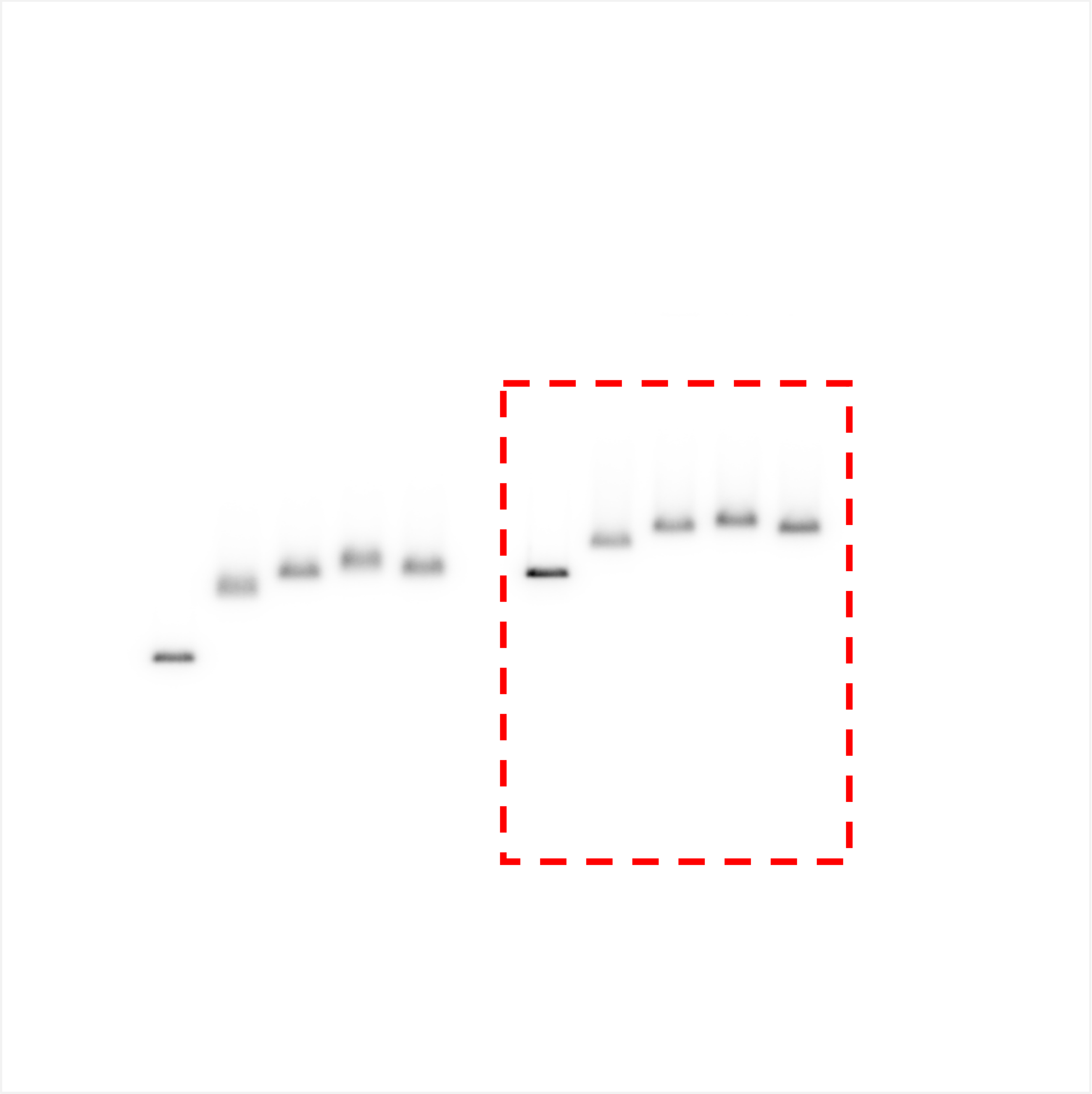

Supplementary Fig. 2C

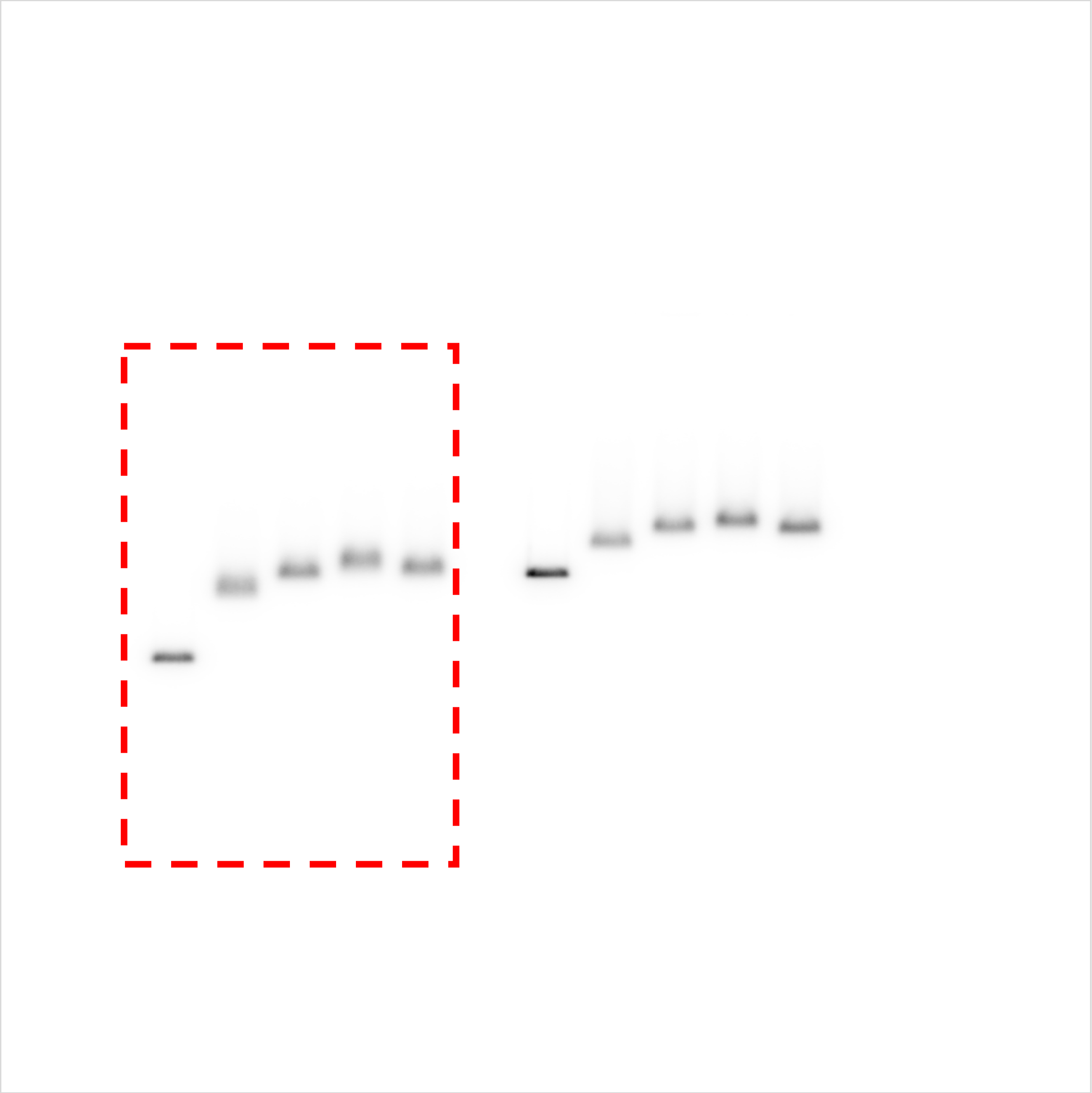

Supplementary Fig. 2D

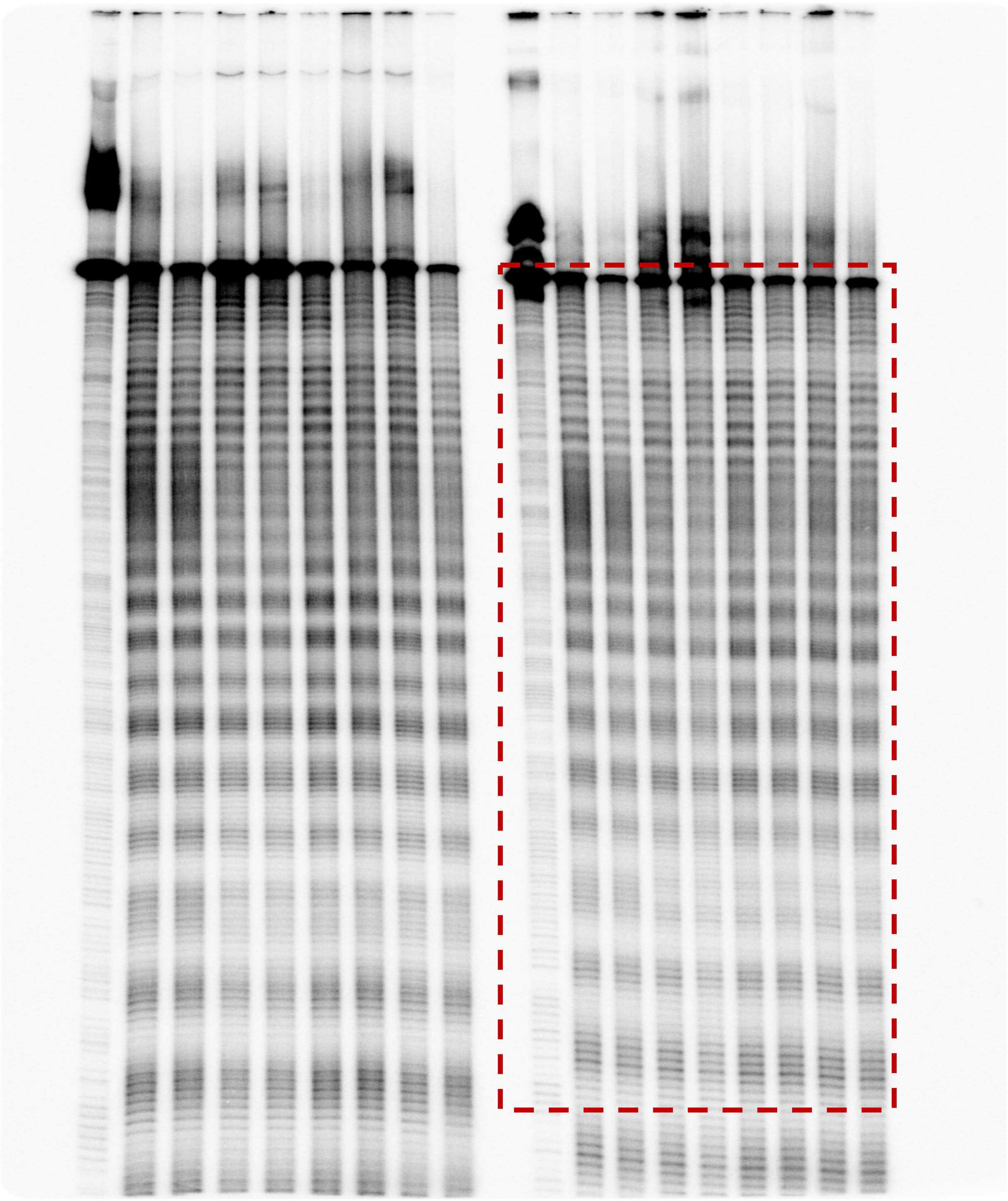

Supplementary Fig. 3A

WT

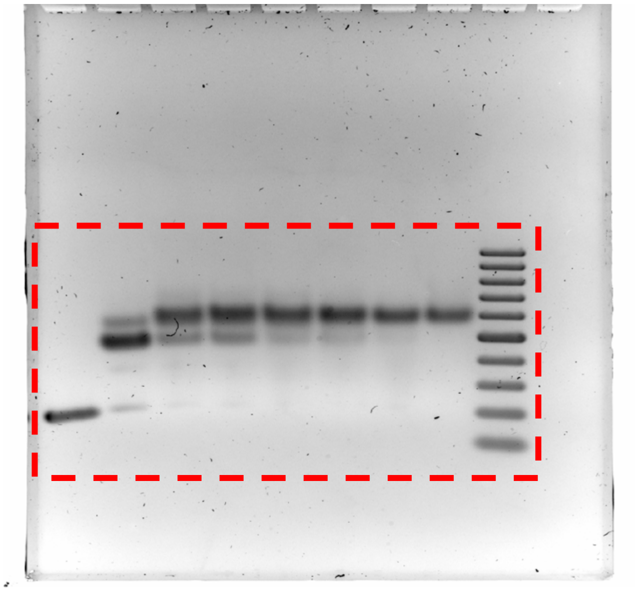

RS

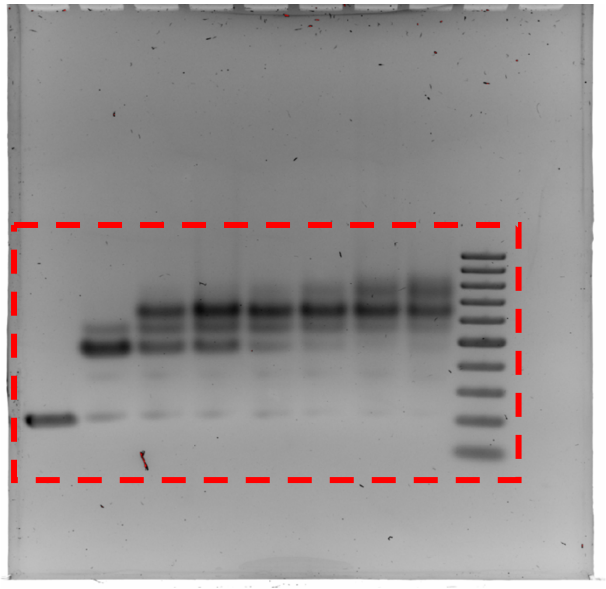

RSΔC

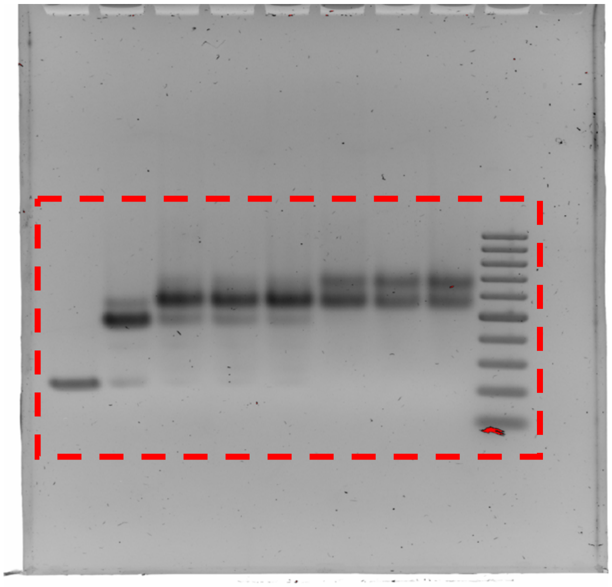

Supplementary Fig. 3B

WT

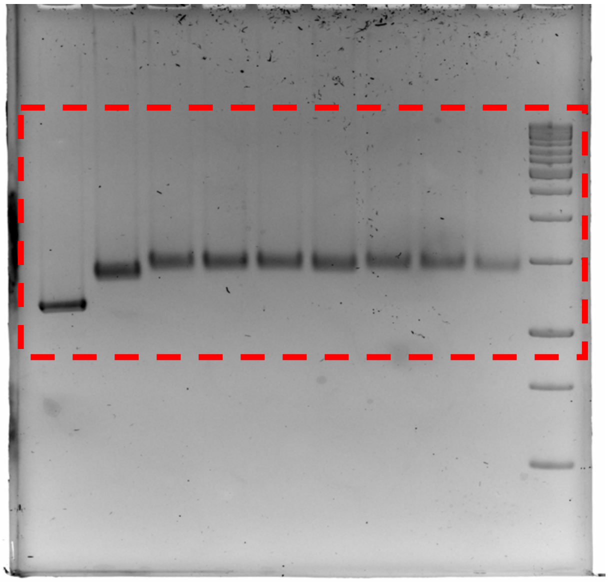

RS

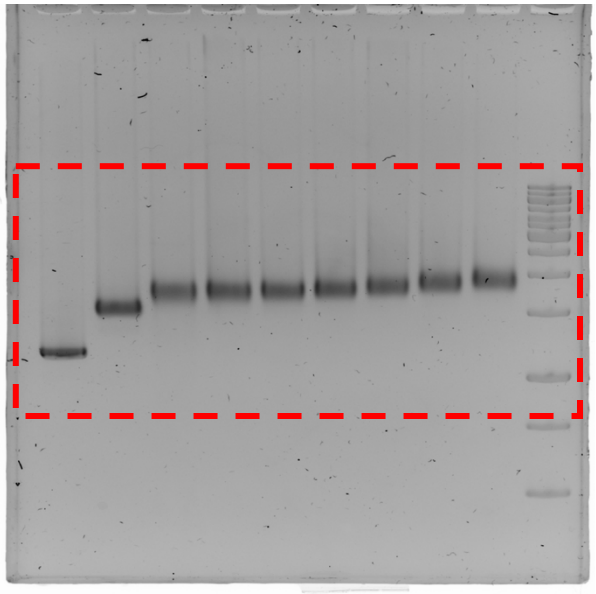

RSΔC

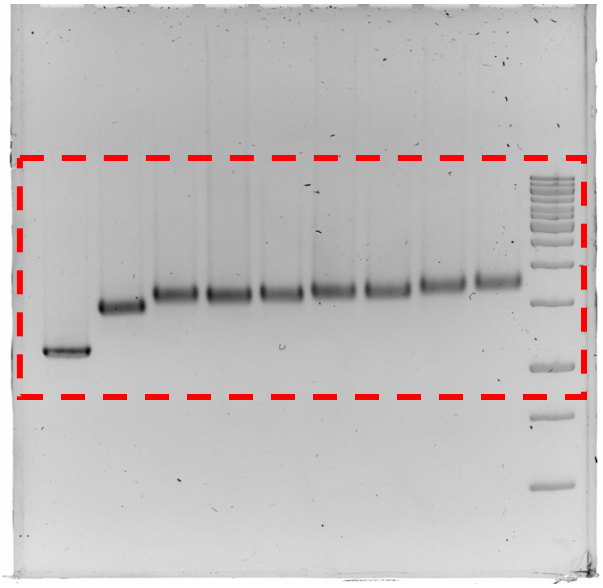

Supplement: Supplementary file 1 — Supplementary Information [file 41467_2026_73046_MOESM1_ESM.pdf]
