# Supplementary material for: A Rahman Syndrome mutation in histone H1.4 disrupts chromatin compaction and phase separation
Source: Nat Commun. 2026 May 22;17:6727. doi: 10.1038/s41467-026-73046-8 (PMC13385758; doi:10.1038/s41467-026-73046-8)
Supplement: Supplementary file 4 — Description of Additional Supplementary Files [file 41467_2026_73046_MOESM4_ESM.pdf]

Description of Additional Supplementary Files

File Name: Supplementary Movie 1

Description: MD simulation of a 197-bp nucleosome bound to WT human histone H1.4.

File Name: Supplementary Movie 2

Description: MD simulation of a 197-bp nucleosome bound to the RS mutant of histone H1.4.

File Name: Supplementary Movie 3

Description: DNA pulling simulation with WT human histone H1.4.

File Name: Supplementary Movie 4

Description: DNA pulling simulation with the RS mutant of histone H1.4.
