# Supplementary material for: A Rahman Syndrome mutation in histone H1.4 disrupts chromatin compaction and phase separation
Source: Nat Commun. 2026 May 22;17:6727. doi: 10.1038/s41467-026-73046-8 (PMC13385758; doi:10.1038/s41467-026-73046-8)
Supplement: Supplementary file 2 — Transparent Peer Review file [file 41467_2026_73046_MOESM2_ESM.pdf]

nature portfolio

Peer Review File

A Rahman Syndrome mutation in histone H1.4 disrupts chromatin compaction and phase separation

Corresponding Author: Dr Carlo Petosa

This file contains all reviewer reports in order by version, followed by all author rebuttals in order by version.

Version 0:

Reviewer comments:

Reviewer #1

(Remarks to the Author)
Boopathi et al investigated the effects of an RS-associated mutation on chromatin structure and dynamics, focusing on H1.4-bound nucleosomes and hexanucleosomal arrays. It was found that RS mutation c.430dupG induces a more extended and flexible array conformation, characterized by enhanced linker DNA accessibility and an inability to form compact, regularly stacked nucleosome structures. Mutant H1.4-bound arrays show a reduced capacity to undergo liquid-liquid and liquid-solid phase separation, closely resembling linker histone-free arrays. Molecular dynamics simulations analysis indicates that the mutated CTD interacts with a shorter linker DNA segment, resulting in a more open nucleosome conformation. The RS mutation significantly enhances H1.4 mobility within cell nuclei, reflecting a weaker chromatin association. Authors concluded that data suggest that RS mutations promote an aberrantly relaxed chromatin state. The include RSΔC mutant is a good design. However the results and comparison of RS and RS ΔC are intriguing and difficulty to interpret or conclude in term of the relevance of the findings to the pathophysiology of RS. As reviewer noted that the complete null of H1-4 mutation in both human and mice show little evidence of functional impairments. The nearly identical changes of chromatin structure for RS and RS ΔC were observed almost all in vitro experiments except Cryo-EM. FRAP experiments using mutant cells also did not detect the difference between RS and RSΔC. However, the functional consequences in human for RS and RSΔC mutant are clearly different. There is no functional evidence that H1-4 mutants missed CTD such as RSΔC in human are discases causing and same in mouse H1-4 null mutant. Thus it raises an important question whether the results from these assays reflect the disease mechanism endogenously or whether these assays are specific enough to detect the potential dominant negative mechanism associated with RS c430dupG mutation.

• Could some of these assays distinguish Gain of function vs dominative negative mechanism of RS?
• Could Reconstitution of hexanucleosome arrays be done in a mixture of RS and WT and RSΔC and WT? This experiment may allow to examine whether RS mutation affect the function of normal H1-4 linker histone. Similar questions may be asked for other assay?
• The data in Figure 5E and F was generated from H1-4 RS and RSΔC mutant cells in vivo. However it was not clear whether RSΔC cell is also stem cells. The images in 5E and 5F seemed drastic different. But the quantification of RS and RSΔC is nearly identical. If the method could not detect the difference between RS and RSΔC, it again raises whether the method is sensitive or valid because RS and RSΔ are clearly different related the disease mechanism
• Dose RS still have the same binding capacity with linker DNA?
• Because of these intriguing data, the following conclusion may be premature: “the observed changes in chromatin structure and dynamics are primarily driven by the loss of the WT C terminal sequence caused by the frameshift mutation, rather than by the acquisition of the acidic C terminal motif”

Reviewer #2

(Remarks to the Author)
Summary: In this study, the authors analyze the effect of a frameshift mutation in histone H1.4 on chromatin compaction, a mutation found to be associated with a rare developmental disorder known as Rahman Syndrome (RS). Compared with the positively charged C-terminal region of wild-type histone H1.4, the C-terminal region of the RS mutant is enriched in acidic amino acids. They found that the RS mutation resulted in an aberrant state of chromatin relaxation. To better determine whether this observation results from the increased acidity of the C-terminal region or from the loss of the wild-type sequence of the C-terminal region, the authors also included in their analysis the H1.4 mutant, RSdeltaC, lacking the C-terminal

region. Using a variety of analytical methods, including analytical ultracentrifugation, SAXS, Cryo-EM, LSPS and LLPS, they found that hexanucleosomes bound to RS and RSdeltaC mutans adopted a more relaxed chromatin conformation than hexanucleosomes bound to WT H1.4. The results of MD simulations and FRAP analyses are also consistent with their conclusion that H1.4 mutants are prevented from forming a compact structure.

The analysis of the effects of the RS mutation on chromatin compaction and dynamics, based on a variety of analytical methods, is highly commendable. The manuscript is well written, with results based on convincing experimental data. The results obtained in this study are not, in themselves, extremely unexpected, but provide important insights that are directly related to a disease, in this case Rahman Syndrome, making this study relevant.

Authors are encouraged to respond to the following comments to improve the quality of the manuscript.

Major comments:

1. In the cryo-EM analysis (Figure 3), the authors report that RS and RSdeltaC mutants show a weaker effect on hexanucleosome compaction than WT H1.4. However, as the number of particles shown is too small, it is not clear for which population size such a tendency is observed. It is crucial that the authors quantify the differences observed between WT H1.4 and the RS and RSdeltaC mutants. To this end, the authors should increase the number of particles to be evaluated.
2. Line 236, “we designate the DNA linkers facing the alpha3 helix and L1 loop of H1.4 as "proximal" and "distal", respectively.”: the authors should illustrate the alpha3 and L1 loops in H1.4, to facilitate the reader's understanding.
3. As indicated in the discussion, the RS mutation would induce the RS mutation phenotype despite the presence of H1.4 WT. This strongly suggests that RS mutations may be dominant-negative mutations. The authors also suggest that the RS mutant could occupy the H1.4 WT binding site in the cell, resulting in aberrant chromatin compaction by H1. To better understand this mechanism, in vitro analysis of chromatin compaction and FRAP assays should be performed under conditions where RS mutants and H1.4 WT are present at the same time.
On the other hand, other histone H1 subtypes are very likely to be present in the cell. It would be interesting to know to what extent the effect induced by the RS mutation on H1.4 function revealed here can be complemented by other H1 histones.
4. When preparing nucleosome-H1 complexes, the RS and RSdeltaC mutants were mixed with nucleosomes in the same ratio. The authors should present a figure showing that nucleosome binding efficiency between WT and H1.4 mutants is the same. The same applies to hexanucleosomes. For correct interpretation of the results, it is important to show that the same amount of H1.4 (WT, RS and RSΔC) is bound to hexanucleosomes.
5. In the “Preparation of hexanucleosome arrays” and “Reconstitution of hexanucleosome arrays” sections of the “Methods” part, “6 × 187 bp 601” should be corrected to “6 × 197 bp 601”.

Minor comments:
1. Line 52: (H2A, H2B, H3 and H4)4 should be corrected to (H2A, H2B, H3 and H4)2.
2. The title “Preparation of hexanucleosome array” in the “Methods” part should be replaced by “DNA preparation for hexanucleosome array” or similar. It is preferable to specify that this is a DNA preparation method.

Reviewer #3

(Remarks to the Author)
Rahman Syndrome is linked to frame-shift mutations resulting in truncation of the linker histone H1.4, such that most of the highly positively charged C-terminal tail domain (CTD) is replaced with a sequence that has far fewer positive charges. The authors compare the nucleosome binding characteristics of wild type H1.4 with two truncated proteins. One truncated H1.4 has the additional sequences most commonly found in patients ("RS"); the other truncated H1.4 lacks the additional sequences ("RSdeltaC"). Most of the experiments involve a hexa-nucleosome substrate. The authors find that the RS truncations produce a more extended, flexible hexa-nucleosome array conformation with increased linker DNA accessibility and reduced phase separation ability, resembling arrays lacking H1. FRET analysis reveals that truncated H1.4 interacts with less linker DNA. The authors propose that the H1.4 truncation acts as a dominant negative, competing with wild type H1 molecules for nucleosome binding sites in vivo, resulting in disruption of chromatin folding. The authors suggest that these properties of truncated H1.4 may help to explain the pathology of Rahman Syndrome.

The authors have employed multiple approaches to investigate the biophysical properties of H1.4 and its truncations in hexa-nucleosome arrays. This study is novel, thorough and well-designed. Its major general interest is as a contribution towards an understanding of Rahman Syndrome. The paper is written very clearly. I have the following comments:

1. It seems surprising that a truncation in one H1.4 allele can result in the debilitating symptoms observed in Rahman Syndrome, given the presence of the four other wild type linker histones (H1.1 - H1.5). The authors point out that truncated H1.4 is a dominant negative, which is reasonable. It would be interesting to see what effect an equimolar mixture of wild type H1.4 and truncated H1.4 would have in one of the assays employed here. It might be predicted that wild type H1.4 would dominate, since it appears to bind more tightly to chromatin and may displace truncated H1.4.

2. The truncated H1.4 molecules have lost a large fraction of their positively charged residues and, in the case of RS, they are replaced with a sequence that has many fewer positive charges (Fig. S1). It was not clear to me how the authors are accounting for their observations at the molecular level. I would predict that truncated H1.4 will bind less tightly to DNA (since the CTD binds to DNA much more tightly than the rest of the H1 molecule). I would also predict that truncated H1.4 will only neutralise DNA charge in the nucleosome-proximal portion of the linker, leaving the distal portion of the linker

accessible. Higher Mg concentrations can partially substitute for the loss of linker charge neutralisation in hexa-nucleosomes complexed with truncated H1.4. Truncated H1.4 will give irregular structures because, unlike the full length H1 CTD, Mg ions cannot guide the linker DNA to facilitate stacking in hexa-nucleosomes. Is this what the authors are proposing? A clarifying paragraph would be helpful.

Minor comments;
3. Fig. 1B: The authors could comment on the different mobilities of the hexa-nucleosomes.
4. Line 143: it would be better to use "compaction" instead of "condensation" here, since the latter word is often associated with the phase transitions mentioned later in the paper.
5. Line 149: the scattering profiles are not "strikingly different", but the density distributions are.
6. Line 251: "nucleosomes" should be "mono-nucleosomes" to clarify that this experiment was not done with hexa-nucleosomes.
7. Line 262: "H1 binding to nucleosomes is stabilized by direct contacts with the nucleosomal dyad and both DNA linkers. Conversely, a more open conformation and greater flexibility of the linkers destabilize H1 binding." I found this logic a bit puzzling. The first sentence is fine, but under what circumstances would the second sentence be true, prior to H1 binding?

Reviewer #4

(Remarks to the Author)
I co-reviewed this manuscript with one of the reviewers who provided the listed reports. This is part of the Nature Communications initiative to facilitate training in peer review and to provide appropriate recognition for Early Career Researchers who co-review manuscripts.

Version 1:

Reviewer comments:

Reviewer #1

(Remarks to the Author)
the authors have addressed the comments from this reviewer.

Reviewer #2

(Remarks to the Author)
The authors have appropriately addressed nearly all of my comments, and the revisions appear satisfactory.

Reviewer #3

(Remarks to the Author)
The authors have addressed all my concerns, except for original comment 1:

In the experiments described in new Fig.7A, an equimolar mixture of WT H1.4 and RS H1.4 was mixed with a hexa-nucleosome chromatin template. The sedimentation of this chromatin was compared with that of hexa-nucleosome chromatin formed with WT-H1.4 only and with RS-H1.4 only. In an alternative approach, equal amounts of pre-formed WT-H1.4-chromatin and RS-H1.4-chromatin were mixed. In both cases, an intermediate sedimentation rate was observed relative to WT-chromatin (fast sedimentation) and RS-H1.4-chromatin (slow sedimentation). This experiment shows nicely that RS-H1.4 is less effective than WT H1.4 at compacting hexa-nucleosome arrays (as expected from the loss of positive charges in the C-terminal tail) and that both WT and RS-H1.4 exchange between chromatin fragments. But this result suggests that neither is dominant. If RS-H1.4 is dominant, it should out-compete WT H1. However, there is no competition in this experiment because there are enough nucleosomes to bind all the H1 molecules. Adding excess H1 to chromatin is a tricky experiment due to potential aggregation/precipitation problems. Instead, the authors should discuss the question of whether RS-H1.4 really behaves as a dominant negative.

Reviewer #4

(Remarks to the Author)
I co-reviewed this manuscript with one of the reviewers who provided the listed reports. This is part of the Nature Communications initiative to facilitate training in peer review and to provide appropriate recognition for Early Career Researchers who co-review manuscripts.

Open Access This Peer Review File is licensed under a Creative Commons Attribution 4.0 International License, which permits use, sharing, adaptation, distribution and reproduction in any medium or format, as long as you give appropriate credit to the original author(s) and the source, provide a link to the Creative Commons license, and indicate if changes were made.
In cases where reviewers are anonymous, credit should be given to 'Anonymous Referee' and the source.
The images or other third party material in this Peer Review File are included in the article's Creative Commons license, unless indicated otherwise in a credit line to the material. If material is not included in the article's Creative Commons license and your intended use is not permitted by statutory regulation or exceeds the permitted use, you will need to obtain permission directly from the copyright holder.
To view a copy of this license, visit https://creativecommons.org/licenses/by/4.0/

Response to reviews

We thank the reviewers for their thoughtful and constructive feedback. In revising the manuscript, we undertook substantial additional work to address the technically demanding points raised during review. The revised version now incorporates extensive new experimental work, including additional EMSA, cryo-EM, AUC, phase separation and FRAP data, together with new analyses of our mass photometry results and additional molecular dynamics simulations, amounting to 16 new figure panels and two new movies. We believe that these additions have greatly strengthened the manuscript and comprehensively address the reviewers' concerns, as detailed below.

Reviewer #1:

Boopathi et al investigated the effects of an RS-associated mutation on chromatin structure and dynamics, focusing on H1.4-bound nucleosomes and hexanucleosomal arrays. It was found that RS mutation c.430dupG induces a more extended and flexible array conformation, characterized by enhanced linker DNA accessibility and an inability to form compact, regularly stacked nucleosome structures. Mutant H1.4-bound arrays show a reduced capacity to undergo liquid-liquid and liquid-solid phase separation, closely resembling linker histone-free arrays. Molecular dynamics simulations analysis indicates that the mutated CTD interacts with a shorter linker DNA segment, resulting in a more open nucleosome conformation. The RS mutation significantly enhances H1.4 mobility within cell nuclei, reflecting a weaker chromatin association. Authors concluded that data suggest that RS mutations promote an aberrantly relaxed chromatin state. The include RSΔC mutant is a good design. However the results and comparison of RS and RS ΔC are intriguing and difficulty to interpret or conclude in term of the relevance of the findings to the pathophysiology of RS. As reviewer noted that the complete null of H1-4 mutation in both human and mice show little evidence of functional impairments. The nearly identical changes of chromatin structure for RS and RS ΔC were observed almost all in vitro experiments except Cryo-EM. FRAP experiments using mutant cells also did not detect the difference between RS and RSΔC. However, the functional consequences in human for RS and RSΔC mutant are clearly different. There is no functional evidence that H1-4 mutants missed CTD such as RSΔC in human are discases causing and same in mouse H1-4 null mutant. Thus it raises an important question whether the results from these assays reflect the disease mechanism endogenously or whether these assays are specific enough to detect the potential dominant negative mechanism associated with RS c430dupG mutation.

We thank Reviewer #1 for their constructive comments. We respectfully wish to clarify one aspect of our study to avoid potential confusion. The RSΔC mutant is not a naturally occurring mutation but an engineered truncation used as a mechanistic control. It was included solely to distinguish whether the effects of the RS mutation arise primarily from loss of the native C-terminal sequence or from the gain of the acidic motif created by the frameshift. RSΔC has not been identified in patients and is not a known pathogenic variant, although a comparable truncation could conceivably have clinical consequences. We hope this clarification helps contextualize our responses below.

1. *Could some of these assays distinguish Gain of function vs dominative negative mechanism of RS?*
2. *Could Reconstitution of hexanucleosome arrays be done in a mixture of RS and WT and RSΔC and WT? This experiment may allow to examine whether RS mutation affect the function of normal H1.4 linker histone. Similar questions may be asked for other assay?*

Combined response to points 1 and 2:

We thank the reviewer for these insightful questions, which are closely related. As suggested, we performed the recommended mixing experiments and found that both the RS and RSΔC mutants impair chromatin compaction and liquid-solid phase separation (LSPS) even in the presence of WT

H1.4. Specifically, we reconstituted hexanucleosome arrays in the presence of a 1:1 stoichiometric mixture of WT and either the RS or RSΔC mutant and assessed their sedimentation behavior by analytical ultracentrifugation (AUC) (new **Figure 7A**). In both cases, the mixed arrays displayed intermediate sedimentation profiles relative to WT-only and RS-only arrays. We obtained comparable results when arrays were reconstituted separately with WT or mutant H1.4 and subsequently combined in a 1:1 ratio after reconstitution.

We further characterized these mixed arrays using differential centrifugation assays to assess their LSPS properties (new **Figure 7B**). The mixed WT/RS or WT/RSΔC arrays again displayed intermediate LSPS behavior between that of the WT and mutant arrays. Taken together, these findings demonstrate that the RS and RSΔC mutants attenuate the compaction and phase-separation capacity of WT H1.4, supporting a dominant-negative mechanism in which the mutant protein interferes with the structural function of the WT.

We have incorporated these new data into the revised manuscript as an additional Results subsection, reproduced below:

> **"RS and RSΔC mutants impair chromatin compaction and phase separation in the presence of WT H1.4**
>
> All RS patients identified to date are heterozygous for *H1-4*, expressing both mutant and WT H1.4 variants from different alleles. To model this heterozygous state in vitro, we reconstituted hexanucleosome arrays with an equimolar mixture of WT and either the RS or RSΔC mutant and assessed their degree of compaction by AUC (**Figure 7A**). For both mutants, the mixed arrays displayed sedimentation profiles intermediate between WT-only and mutant-only arrays, indicating partial loss of compaction. Notably, the same intermediate behavior was observed when WT and mutant arrays were reconstituted separately and subsequently mixed in a 1:1 ratio. In the hypothetical case that WT-only and mutant-only arrays persisted as separate species, the post-mixed sample would be expected to exhibit a biphasic sedimentation profile (**Figure 7A**, inset). Instead, a single well-defined boundary was observed, indicating a hydrodynamically homogeneous population consistent with rapid exchange of WT and mutant H1.4 between arrays. Thus, whether mixed during reconstitution or combined post-assembly, the simultaneous presence of WT and mutant H1.4 leads to arrays that exhibit reduced compaction compared to WT-only arrays.
>
> To further characterize these mixed arrays we examined their LSPS behavior using differential centrifugation (**Figure 7B**). The mixed WT/RS arrays displayed intermediate LSPS behavior relative to the WT and RS mutant arrays, with nearly identical results for co-reconstituted and post-assembly mixed arrays. The WT/RSΔC arrays showed a similar trend, although, for reasons not clear, the co-reconstituted sample precipitated at a lower Mg^{2+} concentration than the post-assembly mixed sample. Despite this minor variation, both RS and RSΔC consistently attenuated the ability of WT H1.4 to promote phase separation of nucleosome arrays. Together, these findings demonstrate that the RS and RSΔC mutants antagonize WT H1.4 function, pointing to a dominant negative effect on chromatin compaction and phase separation, likely through competition for nucleosomal binding sites."

3. *The data in Figure 5E and F was generated from H1-4 RS and RSΔC mutant cells in vivo. However it was not clear whether RSΔC cell is also stem cells.*

We thank the reviewer for pointing out this ambiguity. We revised the relevant sentence to clarify that all three H1.4 variants (WT, RS and RSΔC) were expressed in stem cells. The revised text now reads:

"We exploited mouse embryonic stem (mES) cells that we recently developed, in which CRISPR/Cas9-based gene editing was used to replace endogenous H1.4 with photoactivatable GFP-tagged versions of either the WT, RS or RSΔC H1.4 variants[49]".

4. *The images in 5E and 5F seemed drastic different. But the quantification of RS and RSΔC is nearly identical.*

If we have understood correctly, the reviewer's concern appears to stem from the impression that the fluorescence recovery images for the RS and RSΔC mutants in the original **Figure 5E** (now **Figure S13A**) look visually distinct, despite their nearly identical recovery kinetics shown in **Figure 5F** (now **Figure S13B**). However, close inspection shows that the dark circular region corresponding to the bleached spot disappears rapidly in both RS and RSΔC cells (mostly gone by t = 9 s and essentially absent by t = 11 s), whereas in WT cells it persists beyond t = 25 s, consistent with the recovery curves.

We acknowledge, however, that the mouse embryonic stem cell (mES) images are visually difficult to interpret due to the inherently low expression levels of the photoactivatable constructs. To address this limitation, we have now included a second FRAP experiment using U2OS Flp-In cells stably expressing GFP-tagged WT, RS, or RSΔC H1.4 variants (new **Figure 6E** and **6F**). These cells provide stronger fluorescence signals and we have included additional early time points to better illustrate the rapid recovery kinetics of the mutants. In this system, it is easier to visualize that RS and RSΔC behave very similarly, with both recovering markedly faster than WT, fully consistent with the quantitative data and our original mES cell results. Together, the two datasets provide consistent and complementary evidence that the RS and RSΔC mutations substantially increase H1.4 mobility.

5. *If the method could not detect the difference between RS and RSΔC, it again raises whether the method is sensitive or valid because RS and RSΔ are clearly different related the disease mechanism.*

As noted in our initial comment above, RSΔC is an artificial construct used as a mechanistic control, not a naturally occurring mutation. Its role in this study is to dissect the relative contributions of CTD loss versus acquisition of the acidic motif. Accordingly, differences in disease mechanism between RS and RSΔC cannot be meaningfully assessed or inferred. To clarify this explicitly in the manuscript, we have revised line 116 to read as follows (new text underlined):

"We included the RSΔC mutant – an artificial variant not observed in patients – as an investigative tool to assess whether the behavior of the RS variant arises from the loss of C-terminal WT residues or from the acquisition of the acidic C-terminal motif."

6. *Does RS still have the same binding capacity with linker DNA?*

We thank the reviewer for this question. Linker histones interact with multiple DNA surfaces, including the nucleosomal DNA near the pseudodyad and both linker DNA arms. Within compact nucleosome arrays, additional contacts may also form with linker DNA from neighboring nucleosomes. Experimentally, it is extremely difficult to deconvolute the relative contributions of these interfaces to the overal chromatin-binding affinity of H1. However, the question can be addressed computationally. To do so, we performed additional molecular-dynamics (MD) simulations and DNA-binding energy calculations. These analyses show that the RS mutant exhibits a binding affinity for nucleosomal DNA approximately 2 kcal/mol less favorable than that of the WT H1.4 (**new Figure S11**), corresponding to an estimated 26-fold decrease in binding strength. MD simulations performed with the two linker DNA arms and the linker histones show that, whereas WT H1.4 remains stably associated with both linkers, the RS mutant detaches from one arm, underscoring its reduced capacity to bridge and stabilize the linker DNA (new **Movies S3** and **S4**). Together, these findings indicate that the RS mutation weakens the interaction between H1.4 and linker DNA.

We have incorporated these new data into the revised manuscript by modifying the relevant paragraph in the Results section, which is reproduced below (new text underlined):

"Time-dependent trajectories of this distance reveal a substantial loss of compaction of the RS nucleosome, with the mean distance between linker ends increasing from approximately 30 to 38 Å when WT H1.4 is replaced by the RS mutant (**Figure 6C**). In agreement with these observations, the predicted DNA-binding affinity of WT H1.4 was approximately 2 kcal/mol more favorable than that of the RS mutant (–13.10 ± 0.70 vs. –11.09 ± 0.40 kcal/mol; **Figure S11**), corresponding to a 26-fold increase in the dissociation constant (K_D = 0.58 nM vs. 15.2 nM). In complementary DNA pulling simulations involving only the linker histone and nucleosomal linker arms, the WT H1.4 CTD remained stably associated with both DNA arms over a 6 ns trajectory, whereas the RS mutant detached from one of the arms, further underscoring its reduced ability to bridge and stabilize the linker DNA (**Movies S3** and **S4**). Taken together, these findings indicate that the RS mutation weakens CTD-DNA interactions and induces a considerably more open linker arm configuration in H1.4-bound nucleosomes."

7. *Because of these intriguing data, the following conclusion may be premature: "the observed changes in chromatin structure and dynamics are primarily driven by the loss of the WT C terminal sequence caused by the frameshift mutation, rather than by the acquisition of the acidic C terminal motif"*

We thank the reviewer for raising this point. Our conclusion is based on the observation that the RS and RSΔC variants behave similarly across numerous in vitro assays (DNA footprinting, FRET, AUC, SAXS and FRAP). However, we acknowledge that this conclusion applies specifically to the in vitro conditions tested and does not exclude the possibility that the acquisition of the acidic motif may exert additional context-dependent effects in vivo. To clarify this, we have revised the relevant sentence in the manuscript to read as follows (new text underlined):

"These findings indicate that the observed changes in chromatin structure and dynamics, at least under the in vitro conditions tested here, are primarily driven by the loss of the WT C-terminal sequence caused by the frameshift mutation, rather than by the acquisition of the acidic C-terminal motif."

Reviewer #2:

Summary: In this study, the authors analyze the effect of a frameshift mutation in histone H1.4 on chromatin compaction, a mutation found to be associated with a rare developmental disorder known as Rahman Syndrome (RS). Compared with the positively charged C-terminal region of wild-type histone H1.4, the C-terminal region of the RS mutant is enriched in acidic amino acids. They found that the RS mutation resulted in an aberrant state of chromatin relaxation. To better determine whether this observation results from the increased acidity of the C-terminal region or from the loss of the wild-type sequence of the C-terminal region, the authors also included in their analysis the H1.4 mutant, RSdeltaC, lacking the C-terminal region. Using a variety of analytical methods, including analytical ultracentrifugation, SAXS, Cryo-EM, LSPS and LLPS, they found that hexanucleosomes bound to RS and RSdeltaC mutans adopted a more relaxed chromatin conformation than hexanucleosomes bound to WT H1.4. The results of MD simulations and FRAP analyses are also consistent with their conclusion that H1.4 mutants are prevented from forming a compact structure.

The analysis of the effects of the RS mutation on chromatin compaction and dynamics, based on a variety of analytical methods, is highly commendable. The manuscript is well written, with results based on convincing experimental data. The results obtained in this study are not, in themselves, extremely unexpected, but provide important insights that are directly related to a disease, in this case Rahman Syndrome, making this study relevant.

Authors are encouraged to respond to the following comments to improve the quality of the manuscript.

Major comments:

1. In the cryo-EM analysis (Figure 3), the authors report that RS and RSdeltaC mutants show a weaker effect on hexanucleosome compaction than WT H1.4. However, as the number of particles shown is too small, it is not clear for which population size such a tendency is observed. It is crucial that the authors quantify the differences observed between WT H1.4 and the RS and RSdeltaC mutants. To this end, the authors should increase the number of particles to be evaluated.

We thank the reviewer for this constructive comment. To address this point, we performed additional cryo-EM analyses to increase the number of particles evaluated. These experiments are technically highly challenging because H1.4-bound arrays aggregate strongly during grid preparation and very rarely enter grid holes, so acquiring even a single usable micrograph requires extensive sample screening. Given these constraints, we focused our efforts on the WT and RS mutant arrays at the most relevant ionic condition, 0.6 mM $MgCl_2$, which favors the compact twisted state. Because we were unable to obtain sufficient data on RSΔC for statistical analysis, and since this mutant is an investigative tool rather than the primary focus of our study, we omitted it from this section.

The revised manuscript presents new cryo-EM images for WT and RS mutant arrays (shown as overviews in **Fig. S6** and provided in high-resolution in **Supplementary Dataset 1**) and includes analyses of both aggregated and isolated particles. Qualitative inspection of clustered particles revealed that WT clusters frequently contained multiple twisted arrays in juxtaposition, whereas RS clusters rarely contained more than one such particle and were typically composed entirely of irregular, poorly folded arrays (new **Fig. S7**). Statistical analysis of isolated particles (~190 per sample) confirmed this difference: WT arrays were significantly enriched in twisted conformations, whereas RS mutant arrays were enriched in irregular conformations (new **Fig. 4**). Together, these new data provide robust evidence that the RS mutation impairs proper folding of hexanucleosome arrays.

Overall, the revised section includes three new figures (**Figures 4**, **S6** and **S7**) and the text has been rewritten as follows:

> "We next investigated how the RS mutation affects the structure of the hexanucleosome using cryo-electron microscopy (cryo-EM). Although strong aggregation of H1.4-bound arrays during grid preparation precluded 3D reconstruction, the few isolated particles that could be imaged nevertheless yielded valuable insights (**Figure 3A**). Previous work on H1.0b$^{X.lae}$-bound hexanucleosomes showed that arrays imaged at low (5-10 mM) NaCl concentration display a zigzag configuration, whereas those in 0.35 or 0.6 mM $MgCl_2$ exhibit a two-start helical arrangement of stacked nucleosomes, adopting either a flat or twisted conformation, with the twisted form predominating at the higher Mg^{2+} concentration[34] (**Figure 3B**). Arrays bound to WT H1.4 recapitulated this conformational behavior (**Figure 3A).** Arrays bound to the RS mutant also behaved similarly, but appeared qualitatively more extended at low NaCl and less well folded in Mg^{2+}, particularly at the higher concentration, suggesting that the RS mutation hinders arrays from adopting the twisted state.
>
> To confirm this hypothesis, we analysed arrays at 0.6 mM $MgCl_2$ in greater detail, considering both aggregated and isolated particles. Under this condition, both WT and RS mutant samples displayed mixtures of well- and poorly folded particles (high-resolution full-field views in **Supplementary Dataset 1**; overview images in **Figure S6**). However, WT aggregates frequently contained several closely packed, properly twisted particles – most evident in clusters of two or three arrays – whereas RS clusters rarely included more than one such particle and were typically composed entirely of irregular or poorly folded arrays (**Figure S7**). To assess the mutation's effect on isolated arrays, we extracted all clearly discernible, well separated particles (~190 per sample) and classified them by comparison

to simulated views of flat and twisted hexanucleosomes (**Figure 4A**). A technical point is that most WT particles (93%) adopted non-eclipsed orientations, where both nucleosomal stacks of the two-start helix were visible, whereas ~37% of mutant particles adopted eclipsed or partly eclipsed orientations, in which one stack occluded the other (as illustrated in **Figure 3B**). We classified non-eclipsed particles as well-folded (flat or twisted) or poorly folded, with the latter including irregular arrays and a recurrent "mis-twisted" conformation in which one nucleosome is displaced from its stack (**Figure 4A**). We separately scored eclipsed particles as twisted, flat, or irregular, since mis-twisted forms could not be reliably identified in this orientation. Whether considering all particles or only the non-eclipsed subset, the WT and RS samples showed significantly different distributions (**Figure 4B**), with WT arrays containing more twisted conformations and RS arrays enriched in irregular forms (**Figure 4C**). Together, these findings indicate that the RS mutant hinders arrays from adopting the compact twisted state, in both isolated and clustered particles"

2. Line 236, "we designate the DNA linkers facing the alpha3 helix and L1 loop of H1.4 as "proximal" and "distal", respectively.": the authors should illustrate the alpha3 and L1 loops in H1.4, to facilitate the reader's understanding.

The revised version of **Figure 6A** now includes labels indicating the α3 helix and L1 loop, as well as an inset of the H1.4 globular domain with these structural elements labeled.

3. As indicated in the discussion, the RS mutation would induce the RS mutation phenotype despite the presence of H1.4 WT. This strongly suggests that RS mutations may be dominant-negative mutations. The authors also suggest that the RS mutant could occupy the H1.4 WT binding site in the cell, resulting in aberrant chromatin compaction by H1. To better understand this mechanism, in vitro analysis of chromatin compaction and FRAP assays should be performed under conditions where RS mutants and H1.4 WT are present at the same time.

We thank the reviewer for this comment, which relates closely to Reviewer #1's comments concerning potential dominant-negative effects of the RS mutation. As described above, we performed *in vitro* mixing experiments in which WT and mutant H1.4 were present simultaneously. AUC and differential centrifugation assays showed that both RS and RSΔC mutants impair chromatin compaction and liquid-solid phase separation even in the presence of WT H1.4, yielding intermediate sedimentation and phase-separation behavior (new **Figure 7A,B**). These findings support a dominant-negative mechanism in which the mutant protein interferes with the structural function of WT H1.4. (For details, please see response to points 1 and 2 of Reviewer 1 above).

As also suggested by the reviewer, we performed an additional FRAP experiment in U2OS Flp-In cells stably expressing GFP-tagged WT, RS, or RSΔC H1.4 variants integrated at a defined genomic locus. In this system, endogenous H1.4 is retained, allowing us to assess mutant protein dynamics in a WT background. Both RS and RSΔC mutants exhibited markedly increased mobility relative to WT (new **Figure 6E,F**), with recovery half-lives comparable to those measured in the mouse embryonic stem cells lacking endogenous H1.4 (previously **Figure 5E,F**; now **Figure S13A,B**). These results indicate that the high mobility of RS and RSΔC is not substantially mitigated by coexpressed WT H1.4, consistent with an intrinsically reduced chromatin residence time of the mutant proteins.

On the other hand, other histone H1 subtypes are very likely to be present in the cell. It would be interesting to know to what extent the effect induced by the RS mutation on H1.4 function revealed here can be complemented by other H1 histones.

We agree that it would be interesting to investigate whether other H1 subtypes can compensate for the RS mutation in H1.4. However, we consider this question to lie beyond the scope of the present study, which focuses on defining the direct molecular and biophysical effects of the H1.4 RS mutation on chromatin structure and dynamics. This focus represents a key first step toward understanding

Rahman syndrome, which likely arises from disruption of an H1.4-specific function during neurodevelopment, possibly reflecting the distinct spatiotemporal expression or unique interaction profile of H1.4 compared with other subtypes. Determining whether other H1 subtypes can functionally substitute for H1.4 in vivo will be an important future direction for elucidating the disease mechanism.

4. When preparing nucleosome-H1 complexes, the RS and RSdeltaC mutants were mixed with nucleosomes in the same ratio. The authors should present a figure showing that nucleosome binding efficiency between WT and H1.4 mutants is the same. The same applies to hexanucleosomes. For correct interpretation of the results, it is important to show that the same amount of H1.4 (WT, RS and RSΔC) is bound to hexanucleosomes.

We thank the reviewer for this comment. To address this point, we added new EMSA data comparing the incorporation of WT, RS, and RSΔC H1.4 variants into mononucleosomes and hexanucleosome arrays (new **Fig. S3A** and **B**). Samples reconstituted with increasing H1/nucleosome ratios displayed progressive mobility shifts consistent with efficient and comparable incorporation of all three variants under corresponding conditions. Interestingly, in the mononucleosome assays, at H1/nucleosome ratios above 1, the RS and RSΔC mutants produced an additional band, suggesting binding to a secondary site on the nucleosome, whereas the WT sample displayed partial precipitation. Likewise, in the hexanucleosome arrays, WT samples partially precipitated at H1/nucleosome ratios above 1, whereas the mutant arrays showed reduced mobility, consistent with additional H1.4 molecules binding to secondary sites on the array. These distinct behaviours of the WT and mutant H1.4 variants are consistent with our LSPS results showing that WT arrays precipitate under ionic conditions where the mutant arrays remain in solution (**Fig. 5A**).

In addition to the EMSAs, which were performed using newly prepared samples, we reanalyzed our previous mass photometry (MP) data to estimate H1 occupancy of the hexanucleosome arrays used in the original study (new **Fig. S4**). Modeling the mass distribution of the H1-free array as a mixture of species containing between 0 and 6 nucleosomes revealed that the main population comprised arrays with an average of 5.67 nucleosomes (**Fig. S4A**). The mean mass shift observed upon addition of each H1.4 variant yielded the number of H1 molecules bound per array and thus the average occupancy (H1-to-nucleosome ratio), which was approximately 98%, 85%, and 73% for the WT, RS, and RSΔC arrays, respectively (**Fig. S4B**).

Although the RS and RSΔC arrays exhibited slightly lower occupancies than WT, these values likely underestimate true binding under the conditions used in our other assays. Because of intrinsic limitations of the technique, MP measurements were performed at low sample concentration (~15 nM), about 20-fold lower than in the AUC, cryo-EM, and phase-separation assays (0.3 μM). Such dilution promotes partial dissociation, especially for weaker binders. Given the predicted K_d values for WT and RS H1.4 (~0.6 nM and ~15 nM, respectively), the mutant arrays are expected to be more susceptible to dissociation under these conditions. We therefore regard the MP-derived occupancies to represent conservative, lower-bound estimates.

Nevertheless, even assuming that these occupancy values accurately reflect the H1 binding levels in our higher-concentration assays, the modest differences they reveal cannot account for the pronounced biophysical and structural effects observed for the mutant arrays. For example, in the LSPS assay (**Fig. 5A**), the Mg^{2+} concentration required to induce 50% precipitation (C_{50}) is 2.75 mM for the H1-free array and 0.94 mM for the WT-bound array. Based on the relative occupancies, proportional scaling would predict C_{50} values for the RS and RSΔC arrays close to WT (1.19 and 1.44 mM), yet the observed C_{50} values (2.42 and 2.12 mM) are close to those of the H1-free array. Likewise, in cryo-EM analyses, 19.8% of WT-bound arrays exhibit irregular conformations, compared with 52.6% for RS-bound arrays (**Fig. 4B**). The magnitude of this difference far exceeds what could be explained by the small reduction in H1 occupancy.

Together, these results demonstrate that the altered behaviour of the RS and RSΔC arrays reflects a specific functional defect rather than a reduced H1 incorporation. The revised manuscript addresses this point through the inclusion of two new figures (**Fig. S3** and **S4**) and revisions to the first paragraph of the Results section, reproduced below:

> "Electrophoretic mobility shift assays (EMSAs) of mono- and hexanucleosomes reconstituted with increasing amounts of each H1.4 variant confirmed comparable incorporation efficiencies of the WT, RS, and RSΔC forms (**Figure S3**). All hexanucleosome preparations used in subsequent experiments displayed a single band on a native agarose gel, indicating high sample homogeneity (**Figure 1B** and **Figure S2B**). Consistent with these results, mass photometry analysis, which reports on the molecular mass of particles in solution[35, 36], revealed a predominant peak at the expected unbound or H1.4-bound hexanucleosome mass and no detectable aggregates (**Figure 1C**). Modeling of the mass distribution data further indicated similar H1 occupancies across the three H1-bound arrays (**Figure S4**). Together, these results confirm that all three H1.4 variants bind efficiently to nucleosome arrays, yielding homogeneous samples suitable for comparative structural and functional analyses."

The additional explanations given in our comments above are presented in the legends to Figures S3 and S4.

5. In the "Preparation of hexanucleosome arrays" and "Reconstitution of hexanucleosome arrays" sections of the "Methods" part, "6 × 187 bp 601" should be corrected to "6 × 197 bp 601".

We thank the reviewer for alerting us to this potential source of confusion. In fact, all array-based experiments were performed with 6 × 187 bp arrays except for the hydroxyl radical footprinting analysis (Figure 1D), which used 6 × 197 bp arrays because the longer linker DNA improves the resolution of the 10-bp periodicity in the linker stem region. This distinction was not sufficiently clear in the original submission. The revised manuscript now explicitly states the use of two array types in the Results, and all relevant figure legends have been updated to indicate which array was used in each experiment. The Methods section has also been revised to clearly describe the preparation of both array types.

Minor comments:

1. Line 52: (H2A, H2B, H3 and H4)4 should be corrected to (H2A, H2B, H3 and H4)2.

We thank the reviewer for raising this point. In the original sentence, the superscript "4" was not intended to indicate stoichiometry but was a citation referring to reference 4 (Luger et al., *Nature* 1997). However, we recognize that this notation could cause confusion. To improve clarity, we have revised the sentence as follows:

> "The NCP contains ~147 DNA base pairs (bp) wrapped around an octamer of core (H2A, H2B, H3 and H4) histones[4] and connects to adjacent NCPs by a variable length of linker DNA.

2. The title "Preparation of hexanucleosome array" in the "Methods" part should be replaced by "DNA preparation for hexanucleosome array" or similar. It is preferable to specify that this is a DNA preparation method.

Thanks to the reviewer for pointing this out. We have updated the heading accordingly.

Reviewer #3:

Rahman Syndrome is linked to frame-shift mutations resulting in truncation of the linker histone H1.4, such that most of the highly positively charged C-terminal tail domain (CTD) is replaced with a sequence that has far fewer positive charges. The authors compare the nucleosome binding characteristics of wild type H1.4 with two truncated proteins. One truncated H1.4 has the additional sequences most commonly found in patients ("RS"); the other truncated H1.4 lacks the additional sequences ("RSdeltaC"). Most of the experiments involve a hexa-nucleosome substrate. The authors find that the RS truncations produce a more extended, flexible hexa-nucleosome array conformation with increased linker DNA accessibility and reduced phase separation ability, resembling arrays lacking H1. FRET analysis reveals that truncated H1.4 interacts with less linker DNA. The authors propose that the H1.4 truncation acts as a dominant negative, competing with wild type H1 molecules for nucleosome binding sites in vivo, resulting in disruption of chromatin folding. The authors suggest that these properties of truncated H1.4 may help to explain the pathology of Rahman Syndrome.

The authors have employed multiple approaches to investigate the biophysical properties of H1.4 and its truncations in hexa-nucleosome arrays. This study is novel, thorough and well-designed. Its major general interest is as a contribution towards an understanding of Rahman Syndrome. The paper is written very clearly. I have the following comments:

1. It seems surprising that a truncation in one H1.4 allele can result in the debilitating symptoms observed in Rahman Syndrome, given the presence of the four other wild type linker histones (H1.1 - H1.5). The authors point out that truncated H1.4 is a dominant negative, which is reasonable. It would be interesting to see what effect an equimolar mixture of wild type H1.4 and truncated H1.4 would have in one of the assays employed here. It might be predicted that wild type H1.4 would dominate, since it appears to bind more tightly to chromatin and may displace truncated H1.4.

We thank the reviewer for this comment, which relates closely to points 1-2 by Reviewer 1 and point 3 by Reviewer 2. As described above, we reconstituted hexanucleosome arrays in the presence of equimolar WT and either RS or RSΔC H1.4 and analyzed chromatin compaction and phase separation in AUC and LSPS assays (new **Figure 7A** and **B)**. In both assays, the mixed arrays displayed intermediate behaviour between that of WT-only and mutant-only arrays. These results indicate that the RS and RSΔC variants interfere with WT H1.4 function, consistent with a dominant-negative mechanism. For additional details, please see the responses to Reviewer 1 (points 1-2) and Reviewer 2 (point 3) above.

2. The truncated H1.4 molecules have lost a large fraction of their positively charged residues and, in the case of RS, they are replaced with a sequence that has many fewer positive charges (Fig. S1). It was not clear to me how the authors are accounting for their observations at the molecular level. I would predict that truncated H1.4 will bind less tightly to DNA (since the CTD binds to DNA much more tightly than the rest of the H1 molecule). I would also predict that truncated H1.4 will only neutralise DNA charge in the nucleosome-proximal portion of the linker, leaving the distal portion of the linker accessible. Higher Mg concentrations can partially substitute for the loss of linker charge neutralisation in hexa-nucleosomes complexed with truncated H1.4. Truncated H1.4 will give irregular structures because, unlike the full length H1 CTD, Mg ions cannot guide the linker DNA to facilitate stacking in hexa-nucleosomes. Is this what the authors are proposing? A clarifying paragraph would be helpful.

We thank the reviewer for this helpful comment. We agree that the RS and RSΔC truncations would be expected to weaken CTD–DNA interactions, reduce electrostatic neutralization particularly in the distal linker region, and impair the ability of H1.4 to guide and stabilize the two linker arms. To clarify the expected molecular consequences of the RS mutation, we have revised the first two

paragraphs of the Discussion, which now read as follows (new text underlined; red text addresses the reviewer's point regarding charge neutralization restricted to the nucleosome-proximal linker region):

> "Rahman syndrome is associated with C-terminal frameshift mutations in linker histone H1.4, yet the mechanisms linking these mutations to pathogenesis remain unclear. In this study, we used nucleosomes and nucleosome arrays to investigate how a prototypical RS-associated mutation, c.430dupG, affects chromatin structure and dynamics. Hydroxyl radical footprinting revealed that both WT and mutant H1.4 provide similar protection at the nucleosome dyad, confirming correct positioning of the H1.4 globular domain, but that the mutant increases linker DNA accessibility. MD simulations of H1.4-bound nucleosomes rationalized this observation by showing that the RS mutation leads to a more compact CTD, facilitated by electrostatic interactions between its basic residues and the acidic C-terminal motif. Consequently, compared to WT H1.4, the mutant CTD associates with a shorter, core-proximal segment of the distal DNA linker, making it less effective at drawing the two linker arms together. Binding-energy calculations indicated that the RS mutant interacts with nucleosomal DNA approximately 25-fold more weakly than WT H1.4, while DNA-pulling simulations showed that it readily detaches from one linker arm, underscoring its reduced ability to bridge and stabilize the linker DNA. In agreement with the MD and DNA-pulling simulations, FRET assays confirmed that the end-to-end linker DNA distances in RS-bound nucleosomes were increased relative to WT.
>
> The loss of CTD-mediated electrostatic neutralization and the more divergent DNA linker configuration deduced from the MD and FRET analyses are expected to promote a more extended and flexible nucleosome array conformation. Our AUC and SAXS experiments confirmed these structural changes. The convergence of WT and mutant array behavior at higher Mg^{2+} concentrations in these experiments is consistent with Mg^{2+} compensating for the reduced charge screening of the H1.4 mutants through nonspecific electrostatic shielding. Cryo-EM analysis further revealed that arrays bound to the RS mutant are impaired in their ability to form compact structures with regular nucleosome stacking. This deficiency correlates with a reduced propensity to undergo LLPS and LSPS, leading to phase separation behavior resembling that of H1-free arrays. Consistent with a more open, flexible chromatin architecture, our FRAP experiments *in vivo* demonstrated markedly higher mobility for the RS mutant compared to WT H1.4, indicating that RS mutations lead to a more transient and dynamic association of H1.4 with chromatin."

In addition, the fourth paragraph of the Discussion explicitly addresses the RSΔC mutant, clarifying how its behavior compares with that of the RS mutant across our assays:

> "Interestingly, the RSΔC mutant that lacks the acidic C-terminal motif behaved nearly identically to the RS mutant across multiple assays. Specifically, DNA footprinting and FRET experiments showed both mutants to induce a similar increase in linker DNA accessibility and in linker arm divergence; AUC and SAXS experiments revealed the same extended and flexible nucleosome array structures; and FRAP analyses demonstrated a similar elevated mobility within cell nuclei. These findings indicate that the observed changes in chromatin structure and dynamics, at least under the in vitro conditions tested here, are primarily driven by the loss of the WT C-terminal sequence caused by the frameshift mutation, rather than by the acquisition of the acidic C-terminal motif. In contrast, LSPS and LLPS assays revealed distinct behavior between the two mutants, with the RSΔC mutant allowing phase separation at lower $MgCl_2$ concentrations than the RS variant. These findings imply that, while efficient nucleosome array compaction and phase separation rely on the WT C-terminal sequence, the C-terminal acidic motif generated by the RS mutation further hinders the latter process."

Minor comments

We thank the reviewer for the constructive suggestions in points 3-7 and have modified the text accordingly.

3. Fig. 1B: The authors could comment on the different mobilities of the hexa-nucleosomes.
We have now added the following comment to the Figure 1B legend:

> "Despite its reduced net positive charge, the RS variant results in arrays that migrate more slowly than the WT, consistent with a more extended array conformation and possibly also reflecting added hydrodynamic drag from its acidic C-terminal tail, which is predicted to be disordered and not engaged with the DNA. RSΔC-bound arrays migrate faster than those with RS, consistent with loss of the 38-residue (~4 kDa) acidic tail, despite the slightly higher net positive charge of the RSΔC variant (+9 vs. +6)."

4. Line 143: it would be better to use "compaction" instead of "condensation" here, since the latter word is often associated with the phase transitions mentioned later in the paper.
We have made this change.

5. Line 149: the scattering profiles are not "strikingly different", but the density distributions are.
We thank the reviewer for this clarification. Accordingly, we have revised the relevant sentence by changing "strikingly" to "consistently":

> "Across the range of ionic conditions tested, the RS- and RSΔC-bound arrays showed similar scattering profiles that differed consistently from those of WT H1.4-bound arrays (**Figure S5B**)"

6. Line 251: "nucleosomes" should be "mono-nucleosomes" to clarify that this experiment was not done with hexa-nucleosomes.
We have made the suggested change.

7. Line 262: "H1 binding to nucleosomes is stabilized by direct contacts with the nucleosomal dyad and both DNA linkers. Conversely, a more open conformation and greater flexibility of the linkers destabilize H1 binding[47]." I found this logic a bit puzzling. The first sentence is fine, but under what circumstances would the second sentence be true, prior to H1 binding?

The second sentence refers to evidence from ref. 48 (formerly 47), which shows that the presence of the H3 variant CENP-A enhances the flexibility of the two linker arms and consequently impairs histone H1 binding to the CENP-A nucleosome. To clarify the logic and better reflect the evidence, we have revised the sentence to read:

> "Conversely, a more open nucleosome conformation and greater flexibility of the linkers impairs the association with H1 (ref.[48])"

Reviewer #4:

I co-reviewed this manuscript with one of the reviewers who provided the listed reports. This is part of the Nature Communications initiative to facilitate training in peer review and to provide appropriate recognition for Early Career Researchers who co-review manuscripts.

We thank Reviewer #4 for their contribution to the review process.

Response to reviews

We thank the reviewers for their evaluation of the revised manuscript and are pleased that the revisions have addressed most of their concerns. We respond below to the remaining comment.

Reviewer 3.

The authors have addressed all my concerns, except for original comment 1:

In the experiments described in new Fig.7A, an equimolar mixture of WT H1.4 and RS H1.4 was mixed with a hexa-nucleosome chromatin template. The sedimentation of this chromatin was compared with that of hexa-nucleosome chromatin formed with WT-H1.4 only and with RS-H1.4 only. In an alternative approach, equal amounts of pre-formed WT-H1.4-chromatin and RS-H1.4-chromatin were mixed. In both cases, an intermediate sedimentation rate was observed relative to WT-chromatin (fast sedimentation) and RS-H1.4-chromatin (slow sedimentation). This experiment shows nicely that RS-H1.4 is less effective than WT H1.4 at compacting hexa-nucleosome arrays (as expected from the loss of positive charges in the C-terminal tail) and that both WT and RS-H1.4 exchange between chromatin fragments. But this result suggests that neither is dominant. If RS-H1.4 is dominant, it should out-compete WT H1. However, there is no competition in this experiment because there are enough nucleosomes to bind all the H1 molecules. Adding excess H1 to chromatin is a tricky experiment due to potential aggregation/precipitation problems. Instead, the authors should discuss the question of whether RS-H1.4 really behaves as a dominant negative.

We thank the reviewer for raising this important point. Accordingly, we have revised the Discussion to clarify the interpretation of the WT/RS mixing experiments and the use of the term "dominant negative. The revised text is included below (new or modified text is underlined), followed by additional clarifying comments.

> RS is an autosomal dominant disorder in which all affected individuals reported to date are heterozygous at the *H1-4* locus and therefore express both mutant and WT H1.4. In mice, knockout of a single H1 subtype does not cause a pronounced phenotype or significantly affect development, which only becomes compromised when multiple isoforms are inactivated[53-56]. Given this functional redundancy, the inability of the WT H1.4 allele or other H1 isoforms to compensate for the RS mutation has been interpreted as evidence for a dominant-negative or gain-of-function effect[2]. In line with this view, nucleosome arrays reconstituted with equimolar WT and mutant H1.4 exhibited sedimentation and phase-separation properties intermediate between those of WT-only and mutant-only arrays, consistent with a dominant-negative effect in which incorporation of the mutant protein compromises chromatin compaction in mixed WT/mutant assemblies[57]. A canonical example of such behavior is provided by dimeric transcription factors, where dominant-negative mutations preserve dimerization and DNA binding but disrupt the transcriptional activation domain, such that WT/mutant heterodimers bind DNA normally yet are defective in transcriptional activation[58]. Analogously, RS H1.4 retains the ability to bind nucleosomes but forms mixed WT/RS chromatin assemblies that are compromised for chromatin compaction and phase separation. While our data support a dominant-negative mode of action, we cannot exclude the possibility that RS-associated mutations may additionally exert gain-of-function effects *in vivo* that are not captured by the experimental approaches used in this study.
>
> Our data show that the RS mutation contributes to a more relaxed chromatin structure in two ways. First, when bound to nucleosomes, the RS variant promotes an extended and flexible chromatin conformation with phase separation properties resembling that of H1-free chromatin. Second, the RS variant displays increased mobility within the nucleus, indicating a shorter chromatin residence time. While nucleosomal sites transiently made vacant by the dissociation of the RS variant could in principle allow WT H1.4 or other H1 isoforms to rebind and thereby mitigate the effects of the mutation, a nucleosome-bound RS variant causing chromatin to mimic the H1-free state would competitively inhibit such binding, preventing the rescue of chromatin structure and function. Thus, the adverse effect of the RS mutant on chromatin compaction and phase separation under conditions mimicking the heterozygous situation is more likely attributable to the competition between WT and mutant H1.4 for nucleosomal binding sites rather than to the reduced chromatin residence time of the mutant.

Additional Comments

I. Experimental design

As noted by the reviewer, the experiments in Fig. 7 were performed at a total H1:nucleosome ratio of 1, with WT and RS H1.4 present at equal levels (0.5:0.5), such that all H1 molecules could in principle bind available nucleosomal sites. Experiments performed with an excess of total H1 relative to nucleosomes are technically problematic, as they lead to aggregation and/or second-site binding, as shown in Supplementary Fig. 3. Importantly, a total H1:nucleosome ratio close to or below 1 is physiologically relevant (e.g., Woodcock et al., *Chromosome Res* 2006 PMID 16506093). The conditions used in Fig. 7 therefore more closely mimic the heterozygous situation than experiments performed under conditions of H1 excess.

II. Interpretation of intermediate sedimentation behavior

We agree with the reviewer that the intermediate sedimentation behaviour observed upon mixing WT and RS H1.4 indicates that neither variant is dominant in a strict molecular sense, but rather that the two variants behave in a semidominant (codominant) manner. However, a dominant-negative mechanism does not require the mutant protein to out-compete the

WT protein for binding under saturating conditions. Instead, such effects typically arise when mutant and WT proteins co-assemble within the same functional context, such that incorporation of mutant subunits compromises overall functional output. The experiments in Fig.7 show that WT and RS H1.4 coexist within mixed chromatin assemblies whose compaction and phase-separation properties are shifted away from the WT state. Thus, while these experiments do not demonstrate dominance through competitive exclusion, they do reveal functional interference at the level of chromatin organization.

III. Note on terminology

The definition of dominance differs depending on the context. In classical Mendelian genetics, dominance implies that the heterozygous phenotype matches one of the two homozygous states, whereas in medical genetics a pathogenic variant is considered dominant if it manifests clinically in the heterozygous state, regardless of the phenotype in the homozygous state (Zschocke et al., *Nat Rev Genet* 2023, PMID 36806206). In fact, most autosomal dominant conditions in humans are semidominant in the classical sense. Thus, the behavior observed for the RS mutant in Fig. 7 reflects a semidominant effect at the molecular level that may be fully consistent with a dominant effect at the clinical level.

IV. Mechanistic interpretation

To clarify our rationale for describing RS H1.4 as a dominant-negative mutant, we briefly consider the principal mechanisms underlying autosomal dominant disorders: haploinsufficiency, gain-of-function mechanisms and dominant-negative effects.
i) Haploinsufficiency typically reflects insufficient dosage of functional protein, either due to reduced expression or complete loss of function of the mutant allele. This mechanism is not supported by our data, as the RS H1.4 protein is expressed, binds nucleosomes, exchanges dynamically on chromatin and contributes measurably to chromatin organization, albeit less efficiently than WT.
ii) Gain-of-function mechanisms involve enhanced, ectopic or novel activities of the mutant protein. Our experiments revealed that the RS variant displays reduced chromatin-compacting activity rather than increased or novel activity. While we cannot exclude the possibility that RS-associated mutations may exert gain-of-function effects in vivo, such effects are not supported by the data presented in our study.
iii) Dominant-negative effects arise when a mutant protein interferes with the function of the WT protein in the heterozygous context. Such effects classically occur in the context of multimeric or assembly-dependent systems, in which mutant and WT subunits co-assemble within the same functional complexes. A canonical example is provided by dimeric transcription factors such as yeast GCN4, where mutants lacking the transcriptional activation domain retain DNA binding and dimerization but form transcriptionally defective complexes when assembled with the WT protein. In a heterozygous context, stochastic assembly produces a majority of mutant-containing complexes (WT/mutant heterodimers and mutant homodimers), leading to a marked reduction in overall functional output. Such mutants behave semidominantly at the quantitative molecular level (a WT/mutant heterodimer is more active than a mutant homodimer) while exerting a dominant effect on overall function, considered qualitatively (i.e., normal vs. compromised activity).

The same logic applies to our WT/RS mixing experiments in Fig. 7. Under heterozygous-mimicking conditions, stochastic incorporation of WT and RS H1.4 across nucleosomal binding sites predicts that the vast majority of hexanucleosome arrays contain a mixture of WT and RS molecules rather than being exclusively WT-bound and are therefore at least partly compromised for compaction. RS H1.4 retains the ability to bind nucleosomes and co-assemble with WT H1.4, yet its presence shifts chromatin assemblies toward a less compacted state. In this context, functional impairment emerges at the ensemble level rather than through competitive displacement of WT protein at individual binding sites. Taken together, the observed behavior of RS H1.4 in our experiments is most consistent with a dominant-negative mechanism, as the term is conventionally used in the field.
